# Integrated palliative care and oncology: a realist synthesis

**DOI:** 10.1186/s12916-025-04083-1

**Published:** 2025-05-09

**Authors:** Natasha Bradley, Tracey McConnell, Carolyn Blair, Peter O’Halloran, Gillian Prue, Fiona Lynn, Nia Roberts, Geoff Wong, Elspeth Banks, Joanne Reid

**Affiliations:** 1 School of Nursing and Midwifery, Queen’s University Belfast, Belfast, UK; 2 Marie Curie Palliative Care Research Department, University College London, London, UK; 3 Bodleian Health Care Libraries, University of Oxford, Oxford, UK; 4 Nuffield Department of Primary Care Health Sciences, University of Oxford, Oxford, UK; 5 Living With and Beyond Cancer, National Cancer Research Institute, London, UK

**Keywords:** Palliative care, Oncology, Integration, Realist synthesis

## Abstract

**Background:**

Existing evidence demonstrates the benefits of integrated palliative care for people with cancer, for improved symptom burden, quality of life for patient and caregiver, and appropriate healthcare resource use. The integration of palliative care and oncology has the potential to reduce suffering and is recommended by international guidelines. However, it is not yet consistent practice. There are many approaches to integration, but it is unclear what works, for whom, and in what contexts, to achieve the best possible outcomes for patients, families, and healthcare systems.

**Methods:**

Realist review, conducted in accordance with RAMESES quality standards. Evidence was identified through systematic academic databases searches and stakeholder engagement. Data were extracted from included articles and synthesized using realist analysis to develop explanations of how and why integrated palliative care in oncology works, for whom, and in what contexts.

**Results:**

One hundred sixty-four papers were included in the review, from 33 countries, and involving a range of inpatient, outpatient, and home-based care settings.

Integrated palliative care and oncology could improve patient outcomes, increase the goal-concordance of patient care, and support workforce wellbeing. Interventions towards integration should be tailored to the context in which they are delivered. Ensuring the timely delivery of palliative care for people with cancer requires integration that overcomes siloes between oncology, specialist palliative care, and primary and community care.

The motivation to prioritise the integration of palliative care relies upon all stakeholders first understanding its value. Enriched interdisciplinary collaboration involves developing staff skills and confidence, facilitating coordination between care settings, and supporting communication within and between teams. Leadership is needed at all levels to attend to the structural and social norms of care.

**Conclusions:**

The success of integration is influenced by the ways in which palliative care is understood, prioritised, operationalised, and measured within oncology. Through the synthesis of international evidence, this project draws on implementation science to contribute clarity on how integrated palliative care and cancer care can be achieved in practice.

**Supplementary Information:**

The online version contains supplementary material available at 10.1186/s12916-025-04083-1.

## Background

The World Health Organisation (WHO) defines palliative care as an approach that “improves the quality of life of patients and their families who are facing challenges associated with life-threatening illness” [1]. Palliative care aims to prevent and relieve suffering by identifying and meeting physical, psychological, social and spiritual needs, as well as the needs of caregivers or family. Randomised controlled trials and systematic reviews report that palliative care for people with cancer has significant benefits, especially when delivered early: for symptom burden and quality of life [2–9] and for reduced caregiver stress and depression [8–10]. Available evidence suggests that improving access to palliative care would have economic value in its potential to improve patient experience and outcomes whilst lowering healthcare costs [11].

Recommendations from international and professional groups including the European Association of Palliative Care (EAPC) and the American Society for Clinical Oncology (ASCO) [12, 13] provide endorsement for integration of palliative care alongside cancer treatment, based on the strength of evidence supporting concurrent palliative care. Palliative care can be provided by specialist palliative care teams and by other ‘generalist’ professionals (including nurses, oncology teams, primary care, community care, and increasingly allied health professionals and social workers). The introduction of palliative care is usually a process rather than a single event; and may overlap with other related disciplines including supportive care, geriatric medicine, and survivorship care. Palliative care is considered to be most effective when it is provided ‘early’, and before a crisis. It facilitates improved patient outcomes via enhanced communication and decision-making, longitudinal psychosocial support, and timely symptom management [14]. However, integration of palliative care with cancer treatment is not consistent practice [15, 16]. To alleviate unnecessary suffering, attention to the integration of palliative care is necessary on a global scale [17–19].

Integrated care is an important organising principle, enhancing quality, efficiency, and equity of care. Integration of palliative care aims to achieve continuity of care by bringing together the administrative, organisational, and clinical services that comprise each person’s care network [20]. Three levels of integration have been identified as: full integration (combining resources and teams from different systems or organisations); coordination (individuals or structures provide a bridge to coordinate between different systems or organisations); and linkage (relationships between systems are based primarily in referral practices) [21]. The level and extent of integration between palliative care and oncology varies markedly, between and within different healthcare systems [22].

Promising examples of integrated palliative care and oncology are delivered by a heterogenous mix of multidisciplinary teams, and across inpatient, outpatient, and community settings [15]. Successful integration of palliative care and oncology is complex, including both social and structural changes, involving multiple teams, settings, and organisations. There are differences in how different countries and healthcare systems care for people with cancer, shaped by a myriad of social, cultural, and political contexts. In most healthcare systems, integration necessarily involves different organisations working together so that the different settings and teams involved in patient care can be linked, coordinated, or combined to achieve continuity of care. However, the majority of available evidence with which to guide integration originates from within academic hospitals in high-income countries, which might be less applicable to other situations [21, 23].

A range of barriers to integrating palliative care in oncology have been reported, related to education, regulation, clinical culture, public perceptions, and fragmented or silo-ed care [15, 23, 24]. Consensus is lacking on how to achieve optimal implementation and delivery of integrated palliative care in cancer [15, 25]. Successful and consistent integration may require changes to be made at different levels of the healthcare system and society. Yet, there have been limited attempts to draw from implementation science to gain transferable knowledge and offer real-world solutions [26, 27]. To inform progress, work is needed that synthesizes a deep understanding of how integrated palliative care and cancer care may work best, for whom, in what circumstances. This challenge becomes increasingly urgent as the impact of COVID-19 pandemic continues to ricochet through healthcare systems [19], alongside increasing cancer prevalence and finite resources with which to respond.

This review aims to explain what works, for whom, and in what circumstances to develop optimal integrated palliative care and oncology; and to produce guidance towards improved integration of palliative care for people with cancer. Findings will speak to a broad range of audiences, including healthcare professionals, team leaders, managers, education providers, policymakers, and researchers.

## Methods

The review protocol was registered on PROSPERO (CRD42023389791). Full details of methods can be accessed in the published protocol [28].

Realist synthesis is a theory-driven approach to reviews, frequently used in health research for integrating diverse evidence related to complex interventions [29, 30]. A realist approach to understanding causation can accommodate the heterogeneity of existing evidence by exploring common mechanisms, contextual features, and outcomes, and interrogating the explanatory links between them [31]. Findings are articulated using context-mechanism-outcome configurations (CMOCs), which intend to provide parsimonious and practical insights into the phenomena of interest. The relationships between these CMOCs are usually brought together in a programme theory [32].

Normalisation process theory (NPT) is a sociological theory of implementing and integrating complex interventions, explaining the dynamics of collective effort (or ‘work’) required for people to normalise a particular way of working [33, 34]. NPT has been used widely in health and realist research [35–37]. NPT outlines how social and organisational contexts can generate actions corresponding to four different types of effort that are required to implement (normalise) a new way of working. These are: coherence-building: understanding the work that needs to be done, cognitive participation: involving people in doing that work, collective action: doing the work to make it happen, and reflexive monitoring: assessing and responding to that work [37].

### Patient and public involvement

Our patient and public involvement (PPI) partners (EB, PB, SP, CJ) were involved throughout the review. All four members contributed to discussions with the research team based on their lived experience, reviewed materials for stakeholder meetings, and attended and contributed to the stakeholder meetings. Additionally, EB was a co-applicant on the review grant application, shaped the original funding proposal, participated in monthly research team meetings and associated tasks, and co-authored project outputs.

### Stakeholder engagement

An expert stakeholder group was convened, involving 17 people as representatives from National Health Service (NHS) management and leadership, healthcare professionals involved in the delivery of palliative care and cancer care, public health, palliative care policy groups, international researcher-clinicians who work in integrated palliative care and oncology, and including the PPI partners.

The expert stakeholder group contributed to the realist review during five meetings held throughout the project. Stakeholders were asked to: assist in development of the initial programme theory; support in identifying additional relevant evidence; advise on preliminary and developing findings; consider the implications of findings from their varied perspectives; review and contribute to output materials; and provide input and support for the dissemination strategy [28]. During the meetings, discussions were facilitated by the research team, with each perspective seen as equally important. Meeting notes were agreed with meeting attendees at the end of the meeting. All stakeholder group members had opportunity to comment or contribute reflections in-between meetings.

### Study selection

Data sources were identified through formal searches of academic databases (Medline, Embase, PsycINFO, AMED, CINAHL) (Additional file 1) which took place between July 2023 and January 2024. A supplementary search and hand-searching of key journals was conducted in December 2023. Additionally, stakeholders were invited to contribute literature they felt was relevant up to March 2024.

The initial inclusion and exclusion criteria were intentionally broad and evolved to progressively narrow the focus of the review, in accordance with the published protocol (Table 1). The intention of a realist synthesis is to interrogate causation and develop transferable evidence-based theory that explains the mechanisms through which context influences outcomes. To identify the material most likely to contain relevant, rich and robust information for programme theory development, we prioritised peer-reviewed research articles involving qualitative methods, mixed methods, or health economics in their design, and included all types of participants, i.e., patient, caregivers, healthcare professionals, and others. All care settings were included, to incorporate the continuum of early palliative care during disease management and into end-of-life care. Considering the landmark study published in 2010 [4] (and that only 15% of our search results were published prior to that year) publications from 2010 onwards were included.

**Table 1:** Eligibility criteria

Initial inclusion and exclusion criteria
• Documents focused on integrated palliative care for people with cancer.
• Study design: all study designs, including non-empirical data (e.g. from opinion/commentary pieces) which could inform theory development.
• Types of settings: all care settings including inpatient, outpatient or home-based.
• Types of participants: adults (18 years and over) with a diagnosis of cancer, including underserved groups such as those over 75 years, ethnic minority groups, minority gender identity or sexual orientation, people living in remote areas, and all other potentially underserved groups with a diagnosis of cancer; informal caregivers of people with cancer, and all relevant healthcare professionals.
• Types of intervention: any intervention for providers, patients and/or informal carers, where palliative care and oncology services are managed and delivered so that people with cancer receive a continuum of disease-management, rehabilitation and palliative care services, coordinated across the different levels and sites of care within and beyond the health sector, and according to their needs, from cancer diagnosis to end of life, extending to bereavement support.
• Outcome measures: all integrated palliative care and cancer-related outcomes.
• Language: publications in English language only.
Additional inclusion and exclusion criteria
• Publication year: 2010 onwards
• Study design: including only empirical research of qualitative design, mixed methods, or economic evaluations (full or partial).
• Excluding: conference abstracts, protocols, theses, dissertations, commentaries and literature reviews.

### Data analysis

Articles were imported into NVivo 12 for data extraction. A realist logic of analysis was employed in analysing the data from included documents. Data extraction began by coding into conceptual themes, which were informed by the initial programme theory and other topics identified in the included papers. Interpretive cross-case comparison and retroductive reasoning aimed to establish explanations of how and why the context had influenced observed outcomes and the mechanisms by which this took place [38, 39]. Exploring the data in this way enables evidence-based hypotheses to gradually accumulate and for these to be drawn together into context-mechanism-outcome configurations (CMOCs). Using substantive theory as a broad conceptual framework during realist research can provide a structure within which to organise the numerous programme theories arising from the data [40]. Normalisation process theory [35] was used as an overarching framework for this review, to help interpret the CMOCs during theory development and organise the findings reported here.

## Results

**Fig. 1:**
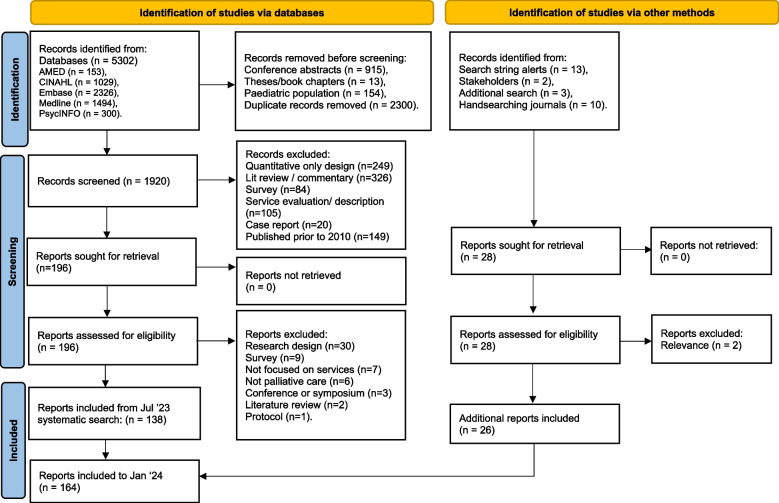
Preferred reporting items for systematic reviews (PRISMA) flow diagram. Adapted from: Page MJ, McKenzie JE, Bossuyt PM, Boutron I, Hoffmann TC, Mulrow CD, et al. The PRISMA 2020 statement: an updated guideline for reporting systematic reviews. BMJ 2021;372:n71. 10.1136/bmj.n71

In total, 164 articles were included in the review (Fig. 1). Information on the characteristics of all included studies is provided in Additional file 2: Table 1 (including research aims, research settings, participants, brief summary of relevance, and a comment on the robustness/limitations of each study).

The majority of documents recruited participants from the USA (*n* = 46), followed by: the UK (*n* = 17), Canada (*n* = 13), Australia (*n* = 11), Norway (*n* = 11), Germany (*n* = 10), the Netherlands (*n* = 7), Belgium (*n* = 7), Switzerland (*n* = 6), Italy (*n* = 6), Japan (*n* = 4), Denmark (*n* = 4), Sweden (*n* = 3), and Iran (*n* = 3). Remaining countries were Brazil, China, France, India, Israel, New Zealand, Nigeria, Singapore, South Africa (*n* = 2 each) and Austria, Czech Republic, Ghana, Hungary, Iceland, Indonesia, Ireland, Jamaica, Jordan, Thailand (*n* = 1 each).

Realist theory development led to the articulation of 18 CMOCs, each of which are explained and accompanied by illustrative quotes in Additional file 3.

**Fig. 2:**
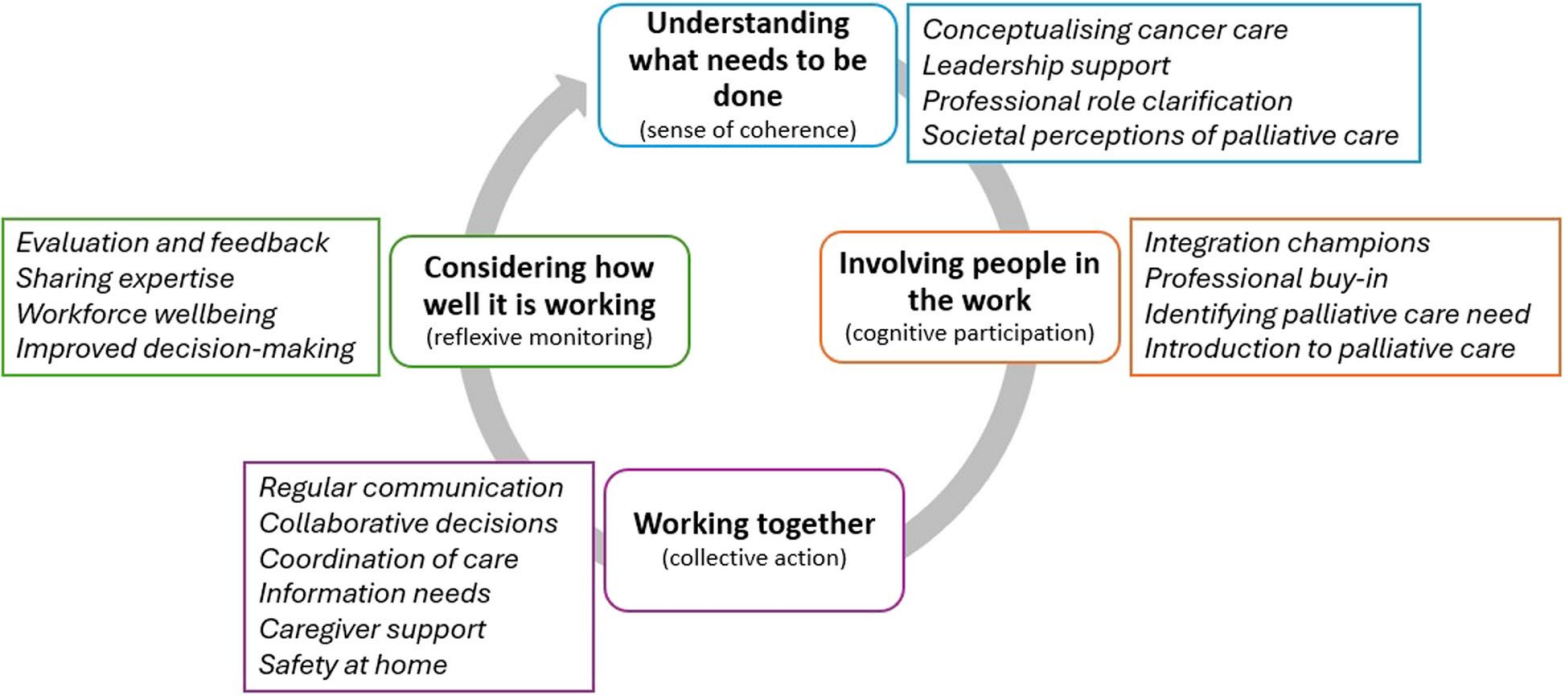
Overview of the structure of review findings

The iterative process of data extraction and synthesis of a large number of articles, alongside stakeholder engagement, led to transferable insights into how and why integrated palliative care in oncology can be successfully implemented and delivered. These findings relate to the contexts in which integrated palliative care comes to be understood, prioritised, operationalised, and measured. Findings are structured in relation to the four primary constructs of normalisation process theory: sense of coherence, cognitive participation, collective action, and reflexive monitoring. An overview of findings is presented in Fig. 2.

### Understanding what needs to be done (sense of coherence)

Sense of coherence (or coherence-building) refers to how people make sense of new ways of working (Additional file 3, box 1, p.1–6). This includes understanding how new ways of working differ from previous practice, the extent to which there is a shared understanding of the aims and anticipated outcomes, how individuals make sense of their roles and responsibilities, and how they understand the potential benefits or value of working in these new ways [35].

Building coherence around integrated palliative care in oncology involves the conceptualisation of cancer care and the ways in which palliative care can be (mis)understood as inapplicable during active treatment or as appropriate only during end-of-life. Oncology and other professionals will be more motivated towards integration if they have experience of palliative care as an essential component of comprehensive cancer care (CMOC1a). These experiences could be provided through the extension of palliative care education, to a wider range of disciplines and embedded throughout professional development.

Where system, organisation, and professional leaders consistently and actively support integration of palliative care, this demonstrates to staff that the endeavour is of collective importance to their institution or profession (CMOC1b). The collective perception that palliative care is worthy of prioritisation may be especially impactful in busy healthcare settings where there are many competing demands. Practical steps for leaders to align organisational structures towards integration include attending to funding streams, incentives, human resources, scheduling, and training, as well as instigating collaboration initiatives between organisations; which facilitate the practical and attitudinal context in which integration can occur.

Effective integration is more likely if individual professionals are clear on their own responsibilities and understand what they can expect from other colleagues, disciplines, or settings (CMOC1c). Providing clarification on roles and expectations would encourage professional confidence and enable more effective collaboration, particularly when real-time communication is more sporadic (e.g. between settings such as primary care and hospital-based oncology teams).

Society’s perceptions of palliative care are also relevant to the public understanding and accepting integrated palliative care. Widespread support is more likely if the value of timely and needs-based palliative care is communicated, conveying the potential benefits and addressing fears or concerns (CMOC1d). This could include public health interventions and positive media campaigns.

### Involving people in the work (cognitive participation)

Cognitive participation refers to the work involved in bringing people together to build and sustain a community of practice around new ways of working (Additional file 3, box 2, p.7–11). This includes how key individuals drive forward new practices, how people organise themselves to contribute to the work involved, the ways in which people are encouraged to see the new ways of working as the right thing to do, and how actions or procedures are defined so that practices can be sustained [35].

Respected oncology clinicians who act to ‘champion’ the integration of palliative care in oncology can inspire other professionals to take part (CMOC2a). However, relying solely on a small number of people to initiate integration could lead to inconsistent or unsustainable implementation. Strategies to achieve wider endorsement include information and involvement activities that are tailored to understand and address the concerns and most meaningful outcomes of relevant clinicians and other professionals (CMOC2b).

Initiatives are required to more consistently identify palliative care needs, which would assist professionals during decisions about when and how to initiate palliative care; for example, through holistic needs assessment being used consistently and accompanied by objective criteria for specialist palliative care referral (CMOC2c). Systematic ‘triggers’ for specialist input should be designed with context-specific knowledge of how different parts of the healthcare system have capacity/competency for different levels of patient complexity. If successful, these initiatives would reduce ambiguity about the appropriate timing of palliative care and allow for more consistent recognition of need.

Health and social care professionals (outside of specialist palliative care, in a range of roles and settings) are generally responsible for introducing the concept of palliative care to people with cancer and their families. As such, they require support and training to become informed, confident, and comfortable in explaining the goals and benefits of palliative care, in order that people with cancer and their families feel reassured, and so more likely to engage (CMOC2 d).

### Working together and becoming a team (collective action)

Collective action refers to the actions people take to enact the new ways of working (Additional file 3, box 3, p.12–20). This includes how people interact, how people build confidence in each other, the division of labour, and the ways in which new ways of working are managed [35].

An important area to consider is the quality and frequency of two-way communication between professionals, which is necessary for them to develop the mutual understanding and trust that underpins effective collaboration (CMOC3a). One-way sharing of written information is less effective in building trust between different disciplines or settings. Strategies to encourage collaborative relationships include providing structure and routine for discussions to take place (e.g., co-location, co-rounding, daily huddles, multidisciplinary clinics, virtual meetings between different locations or settings), and attending to social norms regarding interdisciplinary collaboration.

Collaborative social norms that encourage multidisciplinary team members to contribute and respect different perspectives leads to more holistic decision-making (CMOC3b). This could be influenced by educational and organisational initiatives that provide opportunities for social familiarity and networking. Ensuring that regular communication occurs between different disciplines and teams, and that actions are consistently followed up, indicates the work of coordination. This responsibility needs to be clearly defined and fulfilled by an appropriate team that has the mandate and resource to coordinate care across care settings (CMOC3c).

Working together with people with cancer and their families or informal caregivers requires recognition of their needs; including the need for comprehendible information for them to be enabled to participate in shared decision making (CMOC3d), and for communication and education, and support to help sustain caregivers in their role (CMCO3e). Planning for potential needs while residing at home, and providing straightforward access to responsive support, including out-of-hours, can help people with cancer and their families to trust that help will be available if needed (CMOC3f).

### Considering how well it is working (reflexive monitoring)

Reflexive monitoring refers to the ways in which people appraise their work (additional file 3, box 3, p.21–27). This includes how information is collected that allows people to assess new ways of working, the ways in which groups evaluate their work, and how they do this as individuals, as well as the ways in which these appraisals lead them to redefine or modify their work [35].

It is important to identify and use meaningful metrics for the evaluation of integrated palliative care in oncology, because capturing and providing feedback on progress allows stakeholders to see the benefits of making changes towards more integrated ways of working (CMOC4a). Knowledge of outcomes and patient feedback could help to sustain continued efforts towards integration and the sharing of best practice across the healthcare system.

Increased collaboration between specialist palliative care teams and other professionals provides opportunities for specialist palliative care to share their knowledge and skills, which in turn encourages professionals to collectively feel more confident in providing palliative care (CMOC4b). The extension of specialist palliative care to meet all demand is unlikely, which indicates the need to develop skills and support for generalist palliative care. Resourcing specialist teams to fulfil a training and education remit could therefore strengthen palliative care provision. Without integration, healthcare professionals in oncology or in community settings might feel they are working alone with the emotional challenges of their role. Individual professionals may experience benefits to their wellbeing from integration if improved collaboration corresponds to reduced isolation in ethical and emotional challenges (CMOC4c).

Integration of palliative care in oncology, where characterised by interdisciplinary collaboration and improved communication of needs and preferences, can provide patients, families and professionals with more knowledge and opportunities to reflect during decision-making about goals of care. The care team working together with a shared understanding of the person with cancer’s needs and preferences are thus enabled to make informed decisions, which optimises resource use towards goal-concordant care (CMOC4 d).

## Discussion

Integration of palliative care and oncology is shaped by organisational, clinical, educational and administrative aspects of service delivery [22]. This review extends earlier work by explaining that – despite the financial, political, legal, and cultural nuances between different healthcare systems internationally—there are common challenges, arising from the ways in which integrated palliative care is understood, prioritised, operationalised, and measured within oncology. A summary of recommendations is provided in Table 2.

**Table 2:** Recommendations arising from this review

Audiences	Recommendation
**Section 1: Understanding what needs to be done**	
Education providers and regulatory bodies *For example: universities, medical schools, nursing colleges, allied health professional training programmes, Royal Colleges, other organisations overseeing continuous professional development, General Medical Council, Nursing and Midwifery Council, and other healthcare regulators. *	Integrate *palliative care training and experiential learning* within the education curriculum and professional development for health and social care professionals.
Funders/commissioners *For example: government departments, Health and Social Care Boards, Integrated Care Boards (ICBs).*	Provide appropriate *funding routes and resources *to facilitate integrated education opportunities.
Researchers and knowledge mobilisers *For example: academic researchers and institutions, public health research agencies, research funders (such as National Institute for Health Research, The Health Foundation).*	Encourage organisation and/or system leaders to become *more aware of the benefits *of integrating palliative care, for example by providing evidence of high-value care/return-on-investment.
Organisation and system leadership *For example: NHS leadership and executives, Integrated Care System leads, directors or leads of oncology/cancer care. *	Take actions that promote the integration of palliative care by considering how *funding and incentives, personnel, physical space, performance indicators, and training *could be aligned to prioritise integration.
Organisation and professional leaders *For example: national health organisations and professional bodies such as Royal College of General Practitioners, Royal Pharmaceutical College, Allied Health Professions Federation.*	Provide *clarity on professional responsibilities *for palliative care, especially detailing the role of non-specialists, for example by developing guidelines and supporting the implementation of these guidelines across different care settings.
Public health agencies *For example: public health organisations and devolved public health departments, government health ministries, relevant communication partnerships.*	Research and invest in public health and mass media campaigns to achieve *widespread understanding amongst the public of the benefits *of integration of palliative care for patients and their families (i.e., “what’s in it for you and your family”).
**Section 2: Involving people in the work**	
Organisation/system leaders and managers *For example: NHS leadership and executives, Integrated Care System leads, directors or leads of oncology/cancer care. *	Identify and *support key individuals who act as champions *for integration of palliative care, at different levels of seniority, and in different disciplines/professional groups, including forward planning in case of staff turnover.
Organisation/system leaders and managers *For example: NHS leadership and executives, Integrated Care System leads, directors or leads of oncology/cancer care. *	Plan information and involvement strategies to actively seek out, understand, and *address any concerns of healthcare professionals involved *and to identify outcomes that would be most meaningful to them.
Organisation/system leaders and managers *For example: NHS leadership and executives, Integrated Care System leads, directors or leads of oncology/cancer care. *	Collaborate with oncology and palliative care professionals to introduce and evaluate approaches to *identify palliative care needs more consistently *and across different care settings.
Education providers and regulatory bodies *For example: universities, medical schools, nursing colleges, allied health professional training programmes, Royal Colleges, other organisations overseeing continuous professional development, General Medical Council, Nursing and Midwifery Council, and other healthcare regulators. *	Provide and endorse training that enables (all) healthcare professionals to be *knowledgeable and confident in explaining the goals of palliative care *to patients and families.
**Section 3: Working together and becoming a team**	
Organisation/system leaders and managers *For example: NHS leadership and executives, Integrated Care System leads, directors or leads of oncology/cancer care. *	Ensure appropriate systems, scheduling, and accountability are in place that *enable healthcare professionals to have effective communication *(two-way, respectful discussions) about person-centred care. This could include a code of conduct for communication across care settings, as a core responsibility.
Organisation leaders, managers, and team/service leads *For example: NHS leaders, directors of oncology/cancer care, clinical service managers, senior clinicians and healthcare professionals. *	Encourage *a collaborative working environment* within a team culture of respect, understanding, and communication. This could include education or professional development initiatives towards collaboration and advocacy on behalf of the patient.
Commissioners and leadership across different care settings *Including: acute trusts, primary care, community care, third sector providers. *	Develop and resource *a process that enables coordination of patient care.* For example, supporting a named person or team of people with the necessary skills, credibility and influence to take the lead in coordinating care.
Healthcare professionals *For example: multidisciplinary professionals within oncology teams, palliative care teams, primary care, care navigators. *	Actively seek to understand and *meet the changing informational needs of patients and carers. *This includes ensuring that they are made familiar with community services and routes to further support available to them, ideally prior to a crisis occurring.
Healthcare system information providers *For example: equality departments within hospitals, acute trusts, or integrated care systems, and charity information hubs (such as Macmillan, Marie Curie). *	*Develop informational resources* for people with cancer and their families, tailoring for different needs, including levels of health literacy and for disadvantaged groups.
Healthcare professionals *For example: primary care (General Practitioners, nurses, pharmacists), social workers, allied health professionals.*	Explicitly support the needs of carers, including *sensitive education and honest guidance on what to expect.*
Funders or commissioners, organisation and system leadership *For example: government departments, Health and Social Care Boards, Integrated Care Boards (ICBs), NHS leadership and executives, Integrated Care System leads, directors or leads of oncology/cancer care.*	Invest in *community care, including for out-of-hours,* such as 24/7 helplines and/or rapid response services.
Healthcare professionals *For example: multidisciplinary professionals within oncology teams, palliative care teams, primary care (General Practitioners, nurses, pharmacists), social workers, allied health professionals.*	Recognise that people who live alone or in rural or deprived areas may have additional challenges as patient or carer. Understand and *respond to factors that may prevent or threaten safety at home* (e.g. domestic abuse, neglect, addiction, housing instability).
**Section 4: Considering how well it is working**	
Organisation leaders, managers, and team/service leads *For example: NHS leaders, directors of oncology/cancer care, clinical service managers, senior clinicians and healthcare professionals.*	Develop appropriate and meaningful *strategies for the evaluation of integrated palliative care and for providing feedback *to teams on their progress.
Organisation leaders, managers, and team/service leads *For example: NHS leaders, directors of oncology/cancer care, clinical service managers, senior clinicians and healthcare professionals. *	Facilitate and encourage *collaboration between specialist palliative care and generalist palliative care providers*, including formal training, collaborative meetings, and informal learning-by-doing.
Team/service leads and healthcare professionals *For example: directors of oncology/cancer care, clinical service managers, senior clinicians, multidisciplinary professionals within oncology teams, and palliative care teams. *	Provide and encourage *opportunities for reflection, peer support, and emotional support* between oncology and palliative care team members.
Healthcare professionals *For example: multidisciplinary professionals within oncology teams, palliative care teams, primary care (General Practitioners, nurses, pharmacists), social workers, allied health professionals.*	Foster *communication and reflection about the person with cancer’s needs, goals and preferences*, in relation to the options that are available to them. Where decision-making is routinely informed by communication, collaboration, and reflection on person-centred needs, this helps to achieve high-value care.

The benefits of early palliative care within oncology need to be more widely understood and accepted by all stakeholders, including senior leaders and decision-makers, health and social care professionals, and members of the public. For integration to be optimal, strategies are necessary that improve understanding of the potential benefits of timely integrated palliative care. The individuals enacting change need to recognise integration as relevant to them and an appropriate priority for patient care and the healthcare system.

Efforts to destigmatise palliative care amongst the public would contribute to acceptance of integration by reducing apprehension towards its early involvement in the cancer journey [41]. However, questions around the most appropriate terminology remain unresolved, for example it is suggested that people with cancer and their families might be more receptive to “supportive care” [41] or “symptom/pain specialists” [42]. However, avoiding the term “palliative care” could exacerbate misunderstandings and reinforce its stigmatising connotations with death and dying [43]. A combination of public health and professional education is therefore necessary to build a shared understanding of what palliative care is (and is not).

To improve the conceptual clarity of palliative care and the recognition that it is part of everyone’s role, organisational and discipline leaders should work to clarify the responsibilities of health and social care professionals, for example detailing roles and competencies at the specialist and general level [25]. As general palliative care is an integral part of practice for several professions (e.g. for General Practitioners and District Nurses in the UK), it is difficult to measure [44].

Key findings of this review relate to the need for effective communication and constructive collaboration within and between multidisciplinary teams. Commitment and investment over time are necessary to develop the palliative care capacity of health and social care professionals who do not specialise in palliative care, and to establish social norms of multidisciplinary collaboration within and between teams, which includes (specialist) palliative care. Despite the evidence consensus in favour of integrated palliative care within oncology, consistent implementation can be impeded by competing priorities, social norms, and mismatched resources [45].

Health and social care professionals require focused strategies and time to identify the needs and preferences of people with cancer and their families more effectively [42, 46]. Where needs and preferences are identified, within reason, service organisation and infrastructure should be in place so that this information can be shared appropriately between professional teams and then incorporated into decision-making.

Action is needed from organisation and system leadership, beyond oncology and specialist palliative care. This could include aligning funding streams and key performance indicators with the goals of integration, to encourage service reconfiguration that reduces inefficient duplication and fragmentation of care. For example, National Health Service England (NHSE) improvement funding levers have enabled examples of high-value integrated care that appear to be cost-saving [47, 48].

Since the extension of specialist palliative care teams to meet all demand is unlikely, close attention to the responsibility and skillset for non-specialist palliative care is necessary, including members of the oncology team, primary care, and community nursing. Coordination and effective planning between different settings is essential but does require time, especially when multiple organisations or locations are involved in patient care [49]. Clarification of roles for integration includes recognising the teaching and research remit of specialist palliative care, to enable them in supporting the skills and confidence of other palliative care providers.

Procedural changes that are introduced without a sense of participation among stakeholders may not gain sufficient traction—a ‘hearts and minds’ approach to winning professionals’ endorsement and engagement is important. Interdisciplinary collaboration allows for different disciplines to contribute their perspectives and expertise in identifying unmet need. Trust emerges gradually as a sense of connectedness through commonly shared goals and professional solidarity [50, 51]. Through working together and becoming a team (or a team of teams), professionals learn about each other’s strengths and build trust in each other.

Furthering the spirit of collaborative working to overcome clinical hierarchies and historical silos in health and social care is a cross-cutting challenge for integration [20]. Establishing a culture of collaboration could be supported by strategies such as interdisciplinary education and debrief meetings after sup-optimal experiences [51, 52]. Improved perceptions of team cohesion and increased awareness of the informational needs of other settings can be proximal outcomes in achieving integration by overcoming the fragmentation of care [53, 54].

Integration should seek to achieve collaborative and interdisciplinary decision-making about care that involves the person with cancer as much as possible. The most appropriate way to involve people with cancer and their families in decision-making will be determined by a range of contextual factors, including health literacy [55]. More work is required to acknowledge the breadth of patient situations, such as comorbidities, frailty, and socioeconomic context. Family members or unpaid caregivers provide crucial input and explicit consideration of their physical and mental health is vital [56].

Team culture will influence the success of efforts to include the person with cancer and their families in decision-making. Some oncologists experience discomfort in communicating about prognosis and non-cure focused treatment [57, 58]. Lack of training or personal/professional uneasiness towards the end-of-life may cause them to avoid or obfuscate communication when treatments are no longer likely to be effective [59, 60]. Consequently, in some circumstances, people with cancer undergo unwanted or unhelpful treatments or medical interventions when they do not understand their prognosis and options [61]. Clinical decision-making therefore relies in part on the team’s capacity for reflection and intuition, as resources to engage with the patient [62–64].

Having an appropriate and standardised set of metrics for early integration of palliative care could allow for increased accountability. Progress is required to identify and introduce meaningful evaluation strategies, using metrics that correspond accurately to the goals of those involved. Evaluations of quality of life, patient or family satisfaction, or surveys to measure team functioning/team interdependence may all provide useful insights [65]. It is important that feedback tools can be developed to incorporate patient experience without becoming burdensome. Conducting evaluations of professional experiences could involve group reflective sessions to capture learning and facilitate refinement of quality improvement initiatives [66]. Where the goal of integration is to improve equity of access to palliative care, then this should be articulated and evaluated [67].

### Strengths and limitations

We conducted a substantive review of recent evidence, with extensive stakeholder engagement. The research team was multidisciplinary, and our international expert stakeholder group was broad, but did not include every possible perspective. We did not intend to include all possible evidence sources, and instead prioritised empirical evidence using research designs most likely to contribute to theory development. This may have led to us overlooking quantitative reports on interventions aimed at integration.

The search strategy was designed with input from an information specialist with relevant expertise to identify evidence on integrated palliative care for people with cancer. The search strategy would not have included aspects of healthcare that can be considered to be a part of palliative care but are not described as such in the associated reports. Relying on evidence from peer-reviewed publications introduces an academic bias and this is partially offset by the stakeholder involvement in this review. Research-intensive settings may have additional resources, such as study coordinators, that enable the integration of care.

Professional participants in the included evidence were most commonly nurses or medics. Where relevant, future research should include other roles, such as social workers, paramedics, physiotherapists, administrators, care home staff, and voluntary and community sector organisations. For example, coordination and signposting might be carried out by social workers or patient navigators and it is important not to overlook this contribution [68].

Research locations in the included evidence were predominantly hospital, outpatient, primary care and community settings. There may be other areas that are relevant to achieving integration—e.g. emergency departments, nursing homes, hospice settings. We are therefore less certain about the transferability of our findings to these other settings. In particular, the role of emergency departments in meeting urgent palliative care needs could be rising in line with increasing late presentations and thus requires further consideration [48]. Although the international nature of included evidence reflects several different healthcare systems, low-income countries remain under-represented, and this should be an area for further development.

## Conclusions

This review contributes to the evidence base in integrated palliative care and oncology. Ensuring the timely delivery of palliative care for people with cancer requires practice to overcome historical siloes between different care settings and providers involved in oncology, specialist palliative care, and primary and community care. The present and growing issues of workforce capacity are a substantial impediment to the healthcare system, and decision-makers should recognise the impact that collaboration versus silo-ed working can have on the professionals involved.

There is a need to release resources for building and sustaining multidisciplinary teams, facilitating the development of staff skills and confidence, and the practicalities of communication across care settings and levels. Evidence suggests that this investment would be worthwhile by increasing the goal-concordance of patient care, improving patient experience and outcomes, and supporting workforce wellbeing.

The motivation to prioritise the integration of palliative care relies upon all stakeholders understanding its value. In addition to addressing the structural and social norms of care, we recommend a multi-pronged approach involving mobilisation of existing research evidence to influence system leaders, interdisciplinary education throughout professional curricula to develop skills and a collaborative culture, and the development of public health campaigns tailored to the needs of different communities.

## Supplementary Information

Additional file 1. Search strategy.

Additional file 2. Included articles

Additional file 3. Detailed context-mechanism-outcome configurations

## Data Availability

Data sharing is not applicable to this article as no datasets were generated. Detailed summaries of the analysis are included in the additional files.

## Abbreviations

AMED: The Allied and Complementary Medicine Database
ASCO: American Society for Clinical Oncology
CINAHL: Cumulative Index to Nursing and Allied Health Literature
CMOC: Context-Mechanism-Outcome Configurations
EAPC: European Association of Palliative Care
NHS: National Health Service
NHSE: National Health Service England
NPT: Normalisation process theory
PPI: Patient and public involvement
WHO: World Health Organisation
