# Supplementary material for: Integrated palliative care and oncology: a realist synthesis
Source: BMC Med. 2025 May 9;23:272. doi: 10.1186/s12916-025-04083-1 (PMC12065255; doi:10.1186/s12916-025-04083-1)
Supplement: Supplementary file 1 — Additional file 1. Search strategy. [file 12916_2025_4083_MOESM1_ESM.docx]

Additional File 1: Search Strategy (MEDLINE Example)

|  | [Medline (Ovid MEDLINE® Epub Ahead of Print, In-Process & Other Non-Indexed Citations, Ovid MEDLINE® Daily and Ovid MEDLINE®) 1946 to present](https://ovidsp.ovid.com/ovidweb.cgi?T=JS&NEWS=N&PAGE=main&SHAREDSEARCHID=50tP2u58H8aICuDkgcRRyXABnKRg7M2Al5pDqvWCnowr9r5tyQx6URaUIdr0uisOG) |  |
| --- | --- | --- |
| 1 | exp *Neoplasms/ | 3416944 |
| 2 | Medical Oncology/ | 23631 |
| 3 | (cancer? or oncol*).ti,kf. | 1461934 |
| 4 | 1 or 2 or 3 | 3746602 |
| 5 | palliative care/ or terminal care/ or hospice care/ | 90012 |
| 6 | Hospice and Palliative Care Nursing/ | 2329 |
| 7 | Palliative Medicine/ | 526 |
| 8 | Terminally Ill/ | 6809 |
| 9 | (((palliative or "end of life" or eol or terminal* or hospice* or supportive) and (care or service?)) or eolc).ti,kf. | 44574 |
| 10 | 5 or 6 or 7 or 8 or 9 | [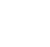](https://ezproxy-prd.bodleian.ox.ac.uk:2483/ovid-a/ovidweb.cgi?&S=DGLOFPGFPGEBIFGIJPPJBHBFJPFIAA00&R=31&Search+Annotations+Options=SA)106833 |
| 11 | Delivery of Health Care, Integrated/ | 14279 |
| 12 | (integrat* adj5 (service? or care or healthcare)).ti,ab,kf. | 45380 |
| 13 | (collaborat* adj5 (service? or care or healthcare)).ti,ab,kf. | 19397 |
| 14 | (collaborat* adj3 (team? or relationship? or multidisciplin* or multi-disciplin* or multiprofessional? or multi-professional? or interdisciplin* or inter-disciplin* or interprofessional? or inter-professional?)).ti,ab,kf. | 16924 |
| 15 | 11 or 12 or 13 or 14 | 84106 |
| 16 | 4 and 10 and 15 | 1468 |
| 17 | ((integrat* or collaborat*) adj (palliative or terminal or hospice or "end of life" or eol or eolc)).ti,ab,kf. | 991 |
| 18 | 4 and 17 | 305 |
| 19 | 16 or 18 | 1494 |
