# Supplementary material for: Integrated palliative care and oncology: a realist synthesis
Source: BMC Med. 2025 May 9;23:272. doi: 10.1186/s12916-025-04083-1 (PMC12065255; doi:10.1186/s12916-025-04083-1)
Supplement: Supplementary file 2 — Additional file 2. Included articles [file 12916_2025_4083_MOESM2_ESM.docx]

Table 1: Included articles

| First Author | Year | Country | Study Design | Aims / Objectives | Setting | Participants | (n) | Relevance to programme theory | Limitations of method / robustness of claims |
| --- | --- | --- | --- | --- | --- | --- | --- | --- | --- |
| Abunasser [1] | 2023 | Jordan | Retrospective cohort study | Assess service utilisation and the impact of palliative care, comparing early (>30 days before death), late (<30 days), and no referral. | Tertiary cancer institution | Breast cancer patients who died between 2014 and 2018 | 433 | Earlier palliative care referral (>30 days before death) associated with less aggressive treatment towards the end of life, and fewer emergency department visits, hospital admissions, intensive care unit (ICU) admissions, without impairing overall survival. Small survival benefit for earlier palliative care group. | Single centre study (largest centre in the country). Did not include length of hospital stay as a relevant variable. |
| Adelson [2] | 2017 | USA | Prospective cohort study | Study the impact of standardized criteria for PC consultation on health service resource use | Inpatient oncology service | Patients (quant) | 113 | Reports that standardised triggers for palliative care consultation during hospitalisation was associated with reduced rates of readmission, reduced chemotherapy following discharge, and suggested reduction in ICU use. Argues that integration should begin in the ambulatory setting to have a greater impact. | Single centre, urban academic context with a mature palliative care service, only included English-speaking patients. |
| Ahmed [3] | 2020 | Canada | Qualitative interviews | Identify experiences of patients with advanced colorectal cancer, and their caregivers, to inform an early palliative care pathway | Two metropolitan tertiary cancer centres | Patients and caregivers | 22 | Identifies gaps in care, including information provision, ambiguity about roles of different professionals, and poor care coordination. Suggests how an early palliative approach would be beneficial. | Two locations. Limited detail on participants given. |
| Alizadeh [4] | 2023 | Iran | Qualitative interviews | Explore barriers to integration from hospital to home and suggest how to resolve them | Focus on home-based palliative care | Healthcare professionals (HCPs), patients, caregivers and others (policy makers, clergy, researcher) | 25 | Recommends implementation of a coordinating nurse, outpatient palliative care clinics, telemedicine, and development of a flexible home-based palliative care model. | Data collected in March-June 2020. Participants had limited experience of home care. Low detail on the participants professions. |
| Back [5] | 2014 | USA | Qualitative focus groups | To understand how clinicians with experience of (early, outpatient) integrated palliative care understand their roles | Cancer centre with experience of randomised controlled trial (RCT) | Healthcare professionals (6 palliative care physicians and 2 advanced practice nurses) | 8 | Over time, palliative care physicians’ expertise in symptom management builds patients trust for them to broach more difficult topics inc. end-of-life (EOL). palliative care (PC) input helps patients to do the emotional work necessary for planning ahead. Additionally, they help to 'interpret' between the patient and the oncology. | A single site with well-established relationships between palliative care and oncology (experience of 2 RCTs) with a 'high dose' intervention of median 10 visits per patient. |
| Bakitas [6] | 2015 | USA | Randomised controlled trial including resource use | Compare effect of early vs delayed initiation of concurrent palliative care intervention (ENABLE) on patient reported outcome measures (PROMs), survival, and resource use | National cancer institute centre, Veterans Affairs medical centre, and community outreach clinic | Patients (quant) | 207 | Reports survival benefit for early PC, without statistically significant increase in resource use despite early enrolment in the intervention. | Contamination of control group was likely. Homogeneity of sample. |
| Bakitas [7] | 2013 | USA | Qualitative interviews | To understand oncologists’ perspectives about concurrent oncology and palliative care in the ENABLE II RCT. | Norris Cotton Cancer Center and VA Medical Center | Healthcare professionals (21 physicians, 14 nurse practitioners, from oncology, haematology, and radiation) | 35 | Early palliative care enhances patient care and complements oncologists practice by 'sharing the load' of complex patient needs. Oncologists engaged with palliative care services as consultants (providing ‘curbside’ advice or limited assistance) and as co-managers or partners on the patient's care team. | Physicians and nurses worked in a team, in a rural, academic cancer centre with a history of integration. |
| Bakken [8] | 2023 | Norway | Qualitative interviews | Explore experiences of how hospital structures influence the recognition and acknowledgement of end-of-life. | One medical and two surgical wards of a Norwegian hospital | Nurses (6) and doctors (6) | 12 | Fast-paced curative-focused medical culture, plus decision-making hierarchies, limit EOL conversations and increase the likelihood of futile treatment. More time, continuity, and collaboration are required for improved care. | Single centre study aligns with findings from elsewhere. Not stated how many individuals were invited to participate - possible that those who did not participate had different views. |
| Beernaert [9] | 2016 | Belgium | Qualitative interviews | Explore palliative care needs (generalist) throughout illness stages for cancer and non-cancer patients. | Recruitment via newsletter, hospital physicians, and palliative home care nurses. | Patients (18, of which with cancer =6) | 18 | Shows how palliative care needs may occur through illness trajectory, indicates role for family physician in identifying these needs, to facilitate needs-based palliative care (which is preferable to prognosis-based). | Applicability from Belgium to UK is unclear (euthanasia). Not all cancer patients. |
| Beernaert [10] | 2015 | Belgium | Qualitative interviews and focus groups | Explore views of patients, community nurses, and family physicians on the role of the family physician (primary care) in providing palliative care. | Recruitment via newsletter, palliative home care teams, and established peer groups of family physicians. | Family physicians (20), community nurses (12), patients (18) | 50 | Identifies tasks for the family physician at different stages of illness including clarifying diagnosis and implications, discussing prognosis, and collaborating with other professionals. | Defines a palliative care approach for primary care, not UK context and not clear how transferable but useful framework to consider. Includes non-cancer as well. |
| Behl [11] | 2010 | USA | Qualitative survey | Elicit oncologists’ views into published statistics of frequency of chemotherapy towards the end of life. | Upper midwestern region of US (Iowa, Michigan, Minnesota, Nebraska, North Dakota, South Dakota, Wisconsin). | Healthcare professionals (Medical oncology and haematology) | 61 | Documents different views on the complexity of decisions about chemotherapy in advanced cancer patients including the potential for over-treatment to be motivated by 'peer pressure' and the availability of new treatment agents. | Response rate of 14%. Most of the findings appear potentially relevant but some contexts may be US specific. |
| Beiranvand [12] | 2022 | Iran | Qualitative interviews | Explain requirements for a hospice system from the perspective of stakeholders | Palliative care (outpatient and inpatient), home care centres, and cancer research centre. | HCPs (physicians, nurses, spiritual counsellors, psychologists and social workers), patients, family caregivers and policymakers | 21 | Outlines requirements for establishing comprehensive hospice care, including clear accountability and coordination/integration across care settings. | Note that this work was carried out prior to introducing hospice centres. |
| Bennardi [13] | 2022 | Switzerland | Qualitative interviews and focus groups | Explore how interprofessional collaboration is experienced between primary care, oncology, and palliative care settings, to identify barriers and facilitators to successful interprofessional collaboration. | Mixture of primary, oncology and palliative care settings. | Healthcare professionals (40 = General practitioners, specialized practitioners, psychologists, nurses, social workers, spiritual assistants, volunteers), 10 key informants (heads of oncology and PC organisations), 10 bereaved family members. | 60 | Interactional and organisational aspects are relevant to interprofessional collaboration. Suggests a key aspect is connectedness between professions (perceiving similar goals, understanding each other’s roles, having regular contact over a period of time). | Does not claim to be generalisable but sampling may be biased towards interest in PC. |
| Bennardi [14] | 2021 | Switzerland | Qualitative interviews and focus groups | Explore how interprofessional collaboration is experienced between primary care, oncology, and palliative care settings, focusing on systemic barriers and facilitators. | Mixture of primary, oncology and palliative care settings. | Healthcare professionals (40 = General practitioners, specialized practitioners, psychologists, nurses, social workers, spiritual assistants, volunteers), 10 key informants (heads of oncology and PC organisations), 10 bereaved family members. | 60 | Identifies issues at the educational, sociocultural, and professional level that influence success of collaboration. | Does not claim to be generalisable but sampling may be biased towards interest in PC. |
| Bergqvist [15] | 2020 | Sweden | Retrospective analysis of health care resource use | Investigate whether expansion of home-based palliative care impacted on resource use or place of death | Home palliative care in Stockholm County Council | Patients (quant) | 2780 | Home based palliative care (HPC) appeared to reduce hospital admissions and emergency department resource use for cancer patients. Lung and GI cancers had more frequent admissions. Comments on the relevance of having support in the home from family and friends. | Retrospective study comparing before and after HPC - no control group. Includes cancer and non-cancer. |
| Bischoff [16] | 2020 | USA | Mixed methods | Explore feasibility and impact of integrated palliative care pilot for metastatic colorectal cancer. | Comprehensive cancer centre and its outpatient palliative care programme: Symptom Management Service | HCPs, patients and caregivers | 30 in intervention group | Offered palliative care to patients routinely and improved collaboration between teams. Positive feedback and indicators of feasibility. Pilot was useful in enabling symptom management and care coordination during transfers of care. | Doesn't seem to mention that they are not calling it palliative care. Data collection strategy less able to reach those with negative experiences of the pilot. |
| Blackhall [17] | 2016 | USA | Retrospective cohort study | Measure timing of referral to outpatient palliative care and the programme's impact on resource use | Academic cancer centre | Patients (quant) | 178 | Reports that outpatient palliative care improved EOL care and reduced healthcare costs, inpatient consultation alone might not be impactful. Discretionary referral by oncologists meant around half of eligible patients were referred. | Referral remained at discretion of oncologist. Not a randomised design. |
| Boyd [18] | 2010 | UK | Qualitative interviews | Understand the feasibility of advanced care planning (ACP) in primary care | Four GP practices in South-East Scotland | HCPs in primary care: 20 general practitioners (GPs), 2 district nurses, 6 practice nurses. | 37 | Suggests multiple barriers to ACP, including limited collaboration with and conflicting information from secondary care. GPs wanted to avoid an overly prescriptive or tick-box approach to advanced care planning, fearing it could compromise their relationship. In most cases waited for a trigger to initiate these conversations, such as discharge from oncology after treatment had stopped, uncontrolled pain, or recognition of dying process. | Data collection occurred when new policies regarding end-of-life-care were being implemented, so it is an evolving situation - although structural and professional barriers to ACP don't seem to have resolved considering more recent papers. |
| Brazil [19] | 2010 | Canada | Qualitative interviews | Examine the stressors associated with caring for a palliative care patient | Home-based family caregivers | Caregivers | 12 | Sources of stress included the formal care system, particularly the number of providers involved, lack of involvement in decisions, and insufficient availability of care. Concludes that an integrated and well-funded system could provide better responsiveness and relational care. | Limited detail on the participants and the existing healthcare system for the people they cared for. Data collected c. 2005. |
| Brenne [20] | 2021 | Norway | Mixed methods | Assess the implementation of a standardised care pathway integrating palliative and oncology services | Specialist and community care | Interviews held with 60 HCPs (specialist physicians, nurses, nurse assistances, community care staff) and 15 patients. Questionnaires sent to 1320 HCPs exposed to the intervention. Analysed data for 129 patient participants. | 75 (qual) | Standardised care pathway appeared to improve quality of palliative care by improving the documentation and transfer of relevant information between specialist and community care. Extensive implementation strategy was required and could have been improved further. Funding limitations and concerns about prioritising this group of patients over another may have been barriers.  Participants endorsed focus on patient needs (not diagnosis/prognosis). Patient held record was useful for medication lists, but only if kept up-to-date. | Good detail on intervention and its implementation. Response rate to survey varied between settings. Large proportion of the patients approach to participate declined (101 out of 231 approached). |
| Bull [21] | 2012 | USA | Quality improvement | Describe organisational aspects relevant to the sustainability of outpatient palliative care | Four Seasons' hospice and palliative care organisation in North Carolina | N/A |  | Used plan-do-study-act cycles in five domains to improve sustainability of outpatient palliative care. Argues that this 'bridging' model is highly cost-effective by preventing gaps in care and rehospitalisation. | Quality improvement cycle in the US – likely to have transferable learning although not mechanistically 'deep'. |
| Capodanno [22] | 2020 | Italy | Qualitative interviews | Explore experience of caregivers with haematological malignancies in palliative home care | The Reggio Emilia Haematological Home Care programme | Caregivers | 17 | Specifies the value of collaborative relationships between caregivers and professionals. | Narrow focus for good insight into what made this home care programme work, especially collaboration with informal caregivers. |
| Cerni [23] | 2023 | Australia | Qualitative focus groups | Identify challenges in rural EOL cancer care, and opportunities for improvement | Rural health district in Australia | Healthcare professionals (10 GPs, 5 palliative care specialists, 5 medical oncologists, 1 respiratory physician, 1 nurse practitioner) | 22 | Identifies specific resource limitations and commissioning frameworks as challenges to EOLC. Difficult access to medication might result in hospitalisation. Continuity of care identified as a key facilitator, also recommends care navigators, especially for socially disadvantaged patients. Suggests use of technology-enabled training and specialist contact. | Multiple disciplines included. Useful detail - some themes are likely to specific to Australian context, but others appear applicable to rural regions elsewhere. |
| Chang [24] | 2018 | USA | Retrospective cost analysis | Compared total costs and length of stay pre-post establishment of Palliative Radiation Oncology Consult service (PROC) | Mount Sinai Hospital Department of Radiation Oncology | Patients (quant) | 181 | Introducing the integrated PROC service was associated with cost-savings, shorter treatment, and increased access to palliative care. | Report focuses on outcomes of implementation of a new service model at a single site. |
| Collins [25] | 2022a | Australia | Qualitative interviews | Explore oncologists' perspectives on communication when introducing palliative care | Metropolitan hospital in Melbourne | Healthcare professionals (medical oncology, surgical oncology, haematology) | 12 | Highlights communication strategies to facilitate referral to palliative care, such as careful use of language to avoid negative associations, drip-feeding information, and framing PC involvement as additive, beneficial, and altruistic. | Single centre study. Considers self-reported and informal communication strategies. Did not capture extent of any previous communication skills training and any impact that may have had. |
| Collins [26] | 2022b | Australia | Mixed methods | Test feasibility of using standardised triggers for introduction of palliative care (in brain, breast, and prostate cancers). | Four metropolitan cancer hospitals | HCPs (n=19 including palliative care nurses, palliative care consultants, oncology nurses, oncology consultants) and descriptive data on patients (n=65) | 84 | Considers the benefits and drawbacks of standardised referral. Automatic identification was more straightforward and thus feasible for the trigger for brain cancer. Triggers chosen for breast and prostate cancer appeared to be too late and added resource demands to the process of identifying patients. | Large cancer centres may not be representative of other hospitals. Only English-speaking patients were eligible. Those participating in clinical trials were also excluded. |
| Collins [27] | 2017 | Australia | Qualitative interviews | Explore patient and caregivers’ initial perceptions of palliative care (before direct experience of PC involvement) | Metropolitan hospital in Melbourne | Patients (mixture of breast, lung, prostate cancers and non-Hodgkin’s lymphoma) and caregivers (19 current, 6 bereaved) | 55 | Palliative care was perceived as a lesser alternative which diminished hope, care, and choice. Euthanasia debate seems to have polarised perceptions so that palliative care is associated with a slow and disempowered death, likely to take place in a hospital. Public health campaign is necessary to communicate rhetoric of choice, accomplishment, and possibility within palliative care, including having hope for meeting personal goals. | Single site study with English-speaking participants. Legal differences to UK around the status of euthanasia. |
| Collins [28] | 2013 | Australia | Qualitative interviews | Explore needs of caregivers in malignant glioma during palliative and bereavement phase | Caregivers, current and bereaved, mostly partners | Caregivers | 23 | Emphasises need for care coordination and continuity. Lack of coordination and incontinuity increases suffering of caregivers, through lack of information, preparation, and consistency. | Findings may be relevant to other cancers, although malignant glioma causes cognitive changes which exaggerate the informational needs and care burden experienced by caregivers. |
| Costantini [29] | 2018 | Italy | Mixed methods | Phase 2 study to assess feasibility and acceptability of early integration of palliative care for respiratory and GI cancer patients. | Outpatient oncology clinics | HCPs (6 oncologists), patients (40, of whom 6 were interviewed), relatives (6 interviewed) | 52 | Availability and responsiveness of the palliative care team was valuable, including as a reassuring presence for expertise if needed. Stigma of 'palliative care' means participants need to be convinced of the added value. | Small number of participants in qualitative aspect. |
| Cushen-Brewster [30] | 2023 | UK | Mixed methods including qualitative interviews | Evaluate a collaborative specialist palliative care service. | Community and hospital setting | Qual: Cancer patients (8), carers (2), staff (12), commissioners (3) | 25 | Co-produced a specialist palliative care service, which enabled upskilling of generalist staff, and positive feedback from patients and caregivers. Covid-19 may have disrupted some of the learning opportunities. | Retrospective study with a small qualitative sample, possible bias in that participants who perceived high-quality of care might have been more likely to take part in the study. |
| Dahlhaus [31] | 2013 | Germany | Qualitative interviews | Explore GPs’ perceptions of palliative cancer care and their constraints | Primary care | Healthcare professionals (13 GPs from seven regions of Germany, mixture of urban/rural, with average 16.6 years’ experience) | 13 | GPs seek collaboration and cooperation with trusted care provider, especially when their expertise or time was limited to respond to the patient's situation or needs. | Approx half of the GPs sampled had specialised in palliative care – might not be representative of the whole profession. |
| Davies [32] | 2014 | UK, Germany, Italy, Netherlands, Norway | Qualitative focus group and interviews | Explore common challenges in providing palliative care for patients with either cancer or dementia | Mixture of organisations across primary, secondary, and tertiary healthcare levels | Purposeful sampling sought experts in policy, service organisation, service delivery, patient groups and palliative care research | 74 | Common challenges are faced across the five countries related to: communication (between services, and with patients/families), infrastructure for integration, insufficient funding, problematic care processes (definitions, knowledge, etc), and time constraints. | Interviews took place in 2012 - possibility of progress since then. |
| Delisle [33] | 2019 | Canada | Retrospective cohort study | Characterise association between palliative care timing, hospital death, and healthcare costs | "On demand" palliative care program in Manitoba | Patients (quant) | 1607 | Strict referral criteria may be a limitation on access to timely PC (e.g., since older patients were likely to die during cancer surgery). Evidence towards early involvement of palliative care - results indicate that late involvement was not as impactful, whereas patients with early or very early palliative care were significantly less likely to visit emergency department (ED), be admitted to hospital, or die in hospital. | Appears focused on specialist palliative care. Does not consider role of or impact on informal caregiving. |
| Desai [34] | 2021 | USA | Qualitative interviews | Examine perceptions of 'palliative care' and 'supportive care' before and after receipt of integrated PC | Cancer Centre in New York State | Patients (21) and caregivers (13) - 48 interviews in total, before and after receiving IPC | 34 | Discerns that terminology of 'palliative care' elicited more apprehension/alarm in this sample than 'supportive care'. Contributes to debate on nomenclature. | Small sample at follow up, does seem to be in line with other findings but not clear if this has been raised in UK context. |
| Dhollander [35] | 2020 | Belgium | Mixed methods | Determine feasibility, acceptability and perceived effectiveness of early palliative home care intervention as a phase 2 pre-post | Palliative home care teams | Patients (16), caregivers (11), oncologists and GPs (11), and a focus group with PHC team (8) | 35 | Reports insufficient interprofessional collaboration and not enough caregiver focus. Argues need to move beyond linkage/coordination of different teams towards collaboration, in which professionals from different settings and disciplines discuss future care. | Useful reporting of strengths and weaknesses of the palliative home care intervention. Not clear which aspects of qual findings came from which source. |
| Dhollander [36] | 2019 | Belgium | Qualitative focus groups | Explore barriers to early integration of palliative care in the home setting from the perspective of palliative home care teams | Palliative home care teams | Healthcare professionals (six palliative home care teams involving 42 palliative nurses, 7 palliative care physicians, 2 psychologists) | 51 | Reports barriers, particularly transmural discontinuity of care. Improving communication between care settings could require better information sharing systems, more contact between professionals (e.g. transmural multidisciplinary meetings) and clearly defined roles and responsibilities (including for communication itself). | Focus groups with insights from different professional perspectives with pre-existing teams, which might have influenced discussions via social desirability. |
| Dhollander [37] | 2018 | Belgium | Qualitative focus groups | Gain insight into how the working practices of palliative home care teams can be affected by early versus late involvement. | Palliative home care teams | Healthcare professionals (six palliative home care teams involving 42 palliative nurses, 7 palliative care physicians, 2 psychologists) | 51 | Key finding was that early involvement (i.e., alongside treatment) creates the need for transmural collaboration and adds coordination tasks to the working practices of home care teams. Being involved early remains advantageous because it allows for planning and a structured approach to holistic care, including empowerment of the patient to participate in shared decision-making (e.g. with their oncologist). | Focus groups with insights from different professional perspectives with pre-existing teams. Possible this format influenced discussions via social desirability. |
| Dhollander [38] | 2022 | Belgium | Qualitative focus groups | Intervention development of the 'Early Palliative Homecare Embedded in Cancer Treatment' (EPHECT) intervention | Palliative home care teams | Healthcare professionals (six palliative home care teams involving 42 palliative nurses, 7 palliative care physicians, 2 psychologists) | 51 | Intervention involves: Education for involved professionals, GP acting as coordinator of care, regular home consultations by palliative homecare teams, semi-structured conversation guide for these consultations, and structured interprofessional and transmural collaboration via telephone. | Useful detail on intervention development, does not seek to evaluate success of the intervention or its components. |
| Economos [39] | 2023 | France | Qualitative interviews | Explore perceptions of oncologists regarding referral to specialist palliative care | University hospitals and a regional cancer centre | Healthcare professionals (medical oncologists or medic working mostly in oncology) | 18 | Barriers include confusion and avoidance of the terminology - that palliative care is associated with imminent death - and need for a referral trigger (such as uncontrollable pain). | These large highly resourced hospitals might not be representative of non-academic hospitals. |
| Ellen [40] | 2021 | Israel | Qualitative interviews | Explore how oncology nurses perceive unnecessary use of health services (such as cancer treatment and diagnostic tests) | Oncology wards at 5 locations | Healthcare professionals (oncology nurses) | 20 | Explores causes and solutions to unnecessary use of healthcare resources. Nurses are aware of unnecessary use but disempowered to challenge physician-centric decision-making. Proposes that strengthening nurses’ role in shared decision making and their authority within the multidisciplinary team could reduce unnecessary use. | Appears to confirm findings from elsewhere - multiple locations here but fairly homogenous sample. |
| Emiloju [41] | 2019 | USA | Retrospective analysis of medical records | Examine relationship between goals of care (GOC) discussions, length of hospital stay, and readmission to hospital within 90 days | Hospital | Hospitalised patients with stage IV solid tumours (aged 26-92, 40.6% female, 55.6% African American). | 241 | Indicates that GOC discussion substantially reduced risk of readmission. Most patients had not had a GOC discussion prior to hospitalisation, but this process enables goal-congruent care. | Analysis of 12 months of admissions within a single hospital system (2 sites). Observational - unable to infer what caused a GOC discussion to occur or not occur. |
| Ervik [42] | 2021 | Norway | Qualitative focus groups and interviews | Explore how to provide equality of care for palliative patients in rural areas | Northern Norway (involved 25 out of the 87 local authorities in the region) | Healthcare professionals (15 cancer nurses, 15 district nurses, 17 GPs, 5 allied healthcare professionals) | 52 | Reports variation in access to palliative care and high caregiver burden. Indicates improving competence to be a key issue, which relies on regional networks and collaborations. | Two (or more) publications from this data collection (2015-2016). Not clear how focus groups vs interviews may have influenced findings. Broad sampling strategy, authors suggest might be generalisable to other rural areas. |
| Ervik [43] | 2023 | Norway | Qualitative focus groups (10) and interviews (6) | Explore experiences of providing palliative care in a rural setting to patients at the end of life. | Staff from a mixture of rural settings | Healthcare professionals - 15 district nurses, 15 oncology nurses, 17 general practitioners, 5 physiotherapists/occupational therapists | 52 | Provides detail on the nuances of providing end-of-life care in the home environment, including the challenges for professional and informal (family) caregivers. | Data collected in 2016-16. Comments that work overload and staff shortages have worsened since then. |
| Evans [44] | 2019 | Canada | Mixed methods | Assesses feasibility, experiences, and impact of the INTEGRATE project | Four cancer centres in Ontario | Descriptive quant data on patients (n=760). Interviews conducted with HCPs (7 oncologists, 3 nurse practitioners, 4 registered nurses, 2 social workers, 2 CCAC care coordinators, 3 project managers, 4 cancer centre directors, 3 CCAC directors) | 28 | Identifies enablers and barriers to the success of the model, including ways the intervention fitted into existing working patterns and the extent of perceived leadership support. Different tumour types were selected by patient need and presence of a clinical champion. | Useful implementation lens on the intervention but does not report on intervention effectiveness or sustainability. |
| Ferrell [45] | 2021 | USA | Qualitative evaluation | One-year follow-up of NCI-funded training program for advanced practice registered nurses (APRNs). | Training programme | Healthcare professionals (oncology nurses) | 276 | The training programme appeared to be effective in extending the role of APRN to include generalist palliative care and the training of other healthcare professionals (train the trainer). Recognises the need for administrative support to attend and to make changes to practice. | One-year evaluation of goals, indicates efforts made by the nurses to influence practice which are useful although design doesn't include whether these changes occurred/whether it impacted on patients. |
| Fox [46] | 2020 | Australia | Qualitative interviews | Explore caregiver experiences with emerging new treatments in metastatic melanoma | Bereaved caregivers, mostly partners to patients who died at home | Caregivers | 20 | Details confusion of caregivers towards goals of newer treatment options, related to increasing prognostic uncertainty and discourse of hope, which has implications for necessary communication competency from clinicians. | Detailed look at processes within metastatic melanoma, could be less applicable to other cancer types. |
| Frissen [47] | 2021 | Netherlands | Qualitative interviews | Assess perspectives and experiences of psychosocial support and palliative care | Specialised hospital oncology, primary care, cancer research/policy institute, university hospital, general hospital, patient organisation | Range of healthcare professionals (inc. oncology) and other professional stakeholders | 16 | Four key themes of opportunities to improve psychosocial support and palliative care for patients with mesothelioma, advises early integration of primary and secondary care, providing tailored and repeated information on availability of support, the usefulness of a case manager, and additional training in psychosocial support and palliative care to reduce inconsistencies in practice. Comment on peer support as a source of information, and potential sources of inequity in informational needs related to socioeconomic status. | Included different occupations but a relatively small number of each. Conclusions on mesothelioma could be applicable to other rare cancers. |
| Garcia [48] | 2023 | USA | Qualitative interviews | Evaluate the Team-based Serious Illness Care Program | Two implementation groups of team-based SICP at Standford | Healthcare professionals (administrative and executive staff, physicians, nurses, rehab staff, chaplains, dieticians, social workers, case managers, middle managers, students and trainees). | 25 | Specifies attributes of small and bounded teams that enable teamwork. Role ambiguity, miscommunication, and ethical conflicts can arise, particularly when teams change frequently, and physician's maintain overall control. More frequent communication enables the 'live' negotiation of roles, which may be especially crucial with staff shortages/turnover. | Detailed account of 'teaming' around a particular intervention, findings may have transferability but unclear whether negotiating roles like this would work within NHS. |
| Gardiner [49] | 2022 | UK | Qualitative focus groups and interviews | Explore perspectives of mesothelioma clinical nurse specialists (CNSs) on their role in providing generalist and specialist palliative care | Mesothelioma CNSs are based in NHS hospitals and funded by Mesothelioma UK | Healthcare professionals (clinical nurse specialists) | 16 | Mesothelioma CNSs provide generalist palliative care from diagnosis to death, educate GPs about the condition, and engage with specialist palliative care services were necessary (but sometimes this is ineffective due to referral pathways). Late diagnosis can mean patients don't meet the MCNS due to not being eligible for treatment and then miss out on this support. | Detailed look at processes within mesothelioma, unclear applicability across cancer types. |
| Gerlach [50] | 2019 | Germany | Qualitative interviews | Explore experiences and views of haemato-oncologists on the surprise question (generally used in MDTs, occasionally with the patient) | Oncology outpatient department of university hospital in German | Healthcare professionals (haematologists with 2-30 years’ experience) | 9 | Surprise question could be valuable tool to shift the Drs perspective towards being patient centred, helpful for them to communicate their intuition to the patient - but impact requires the time and communication skills to have difficult conversations and for the resources of palliative care to be available, including alongside treatment. | Single centre study. Used individual interviews so useful to have this alongside other focus group studies. |
| Gonzalez [51] | 2023 | USA | Retrospective cohort study | Compare healthcare utilisation for patients receiving early or late palliative care referral (PCR), defined as more or less than 30 days after diagnosis. | Academic cancer institution (quaternary) | Pancreatic cancer patients between 2014 and 2020 | 1458 (419 received PCR and inc. in analysis) | Earlier palliative care referral (<30 days after diagnosis) associated with fewer emergency department visits and possibly fewer hospital admissions compared to those who received PCR >30 days after diagnosis. | Compares early and late PCR, although only 28.7% of patients in the study period had a PCR. Smaller sample in the early PCR group (137). Did not examine those who did not receive PCR. Notable that uses time scales forward from diagnosis rather than backwards from death. |
| Gott [52] | 2012 | UK and New Zealand | Qualitative interviews and focus groups | Explore understandings of and roles in the provision of generalist and specialist palliative care | Mixture of primary, secondary, tertiary and residential care settings. | Healthcare professionals. 68 in UK, 77 in NZ. (Job titles: consultant, junior doctor, GP, practice nurse, clinical nurse specialist, other nurse, allied health professional, 'other'; working in acute hospitals, general practice, specialist palliative care units, aged residential care (NZ only) and education/research (NZ only). | 138 | Explains issues related to terminology, perceived roles/ responsibilities, legitimacy of PC within the generalist workload, challenges with partnership working, negative consequences of specialising. | Useful comparison of countries but published in 2011 so situation may have changed especially in relation to trickle-down of policy rhetoric. However, other related studies appear to support the 2012 findings. |
| Greer [53] | 2016 | USA | Randomised controlled trial and cost description | Compare costs of care in intervention and control group, during study period and in last 30 days before death. | Academic cancer centre | Patients (quant) | 138 | Early palliative care associated with lower cost per day on average. Suggests more appropriate resource use because the intervention group were more likely to access hospice and less likely to receive chemotherapy towards the end of life. | Includes different care settings, but single centre study with fairly homogenous sample. |
| Gross [54] | 2019 | USA | Qualitative focus groups | Describe how medical oncologists perceives the role of radiation oncologists, especially towards participation in end-of-life planning | Medical oncologists (almost all practiced in Academic/University hospital settings, mostly in the Midwest region) | Healthcare professionals (medical oncologists) | 31 | Details problematic dynamics between disciplines, professional identities that inhibit teamwork. | May not be generalisable especially outside of the USA - medic motivations informed by financial system. Sampling strategy reached younger, more academic participants which might not be representative. Might expect older generation to be even less inclined towards collaboration. |
| Hahne [55] | 2022 | China | Qualitative interviews | Explore clinician's perspectives on integrating palliative care for advanced cancer patients | Tertiary hospital with no specialised palliative care unit | Healthcare professionals (haematologists - 14 and oncologists - 10) | 24 | Two main conceptualisations of palliative care - i.e., end of life care not compatible with treatment vs an essential component of comprehensive care. Ethical tensions in communicating with family members and collusion may impede integration of palliative care. Recommends education, guidelines, and increased use of family meetings. | Single-site study.  Indicates there are cultural nuances on commonly reported barriers to integration, regarding misunderstandings of its purpose and obstruction from family members. |
| Halling [56] | 2020 | Denmark | Economic evaluation from societal perspective | Evaluate cost-effectiveness of fast-track SPC including an enriched psychological intervention | ‘DOMUS’ trial of home-based SPC | Patients (321) and caregivers (235) | 556 | The intervention appears to improve quality of life and had a mild benefit for caregivers, but at a greater cost than usual care. | Broader perspective on costs measured than other included studies. Possible contamination of control group as could not rule out additional sources of psychological support. |
| Hannon [57] | 2016 | Canada | Qualitative interviews | Solicit opinions of patients and caregivers who participated in RCT of EPC, considering the roles of oncology and palliative care physicians | Recruited from cancer centre in Toronto | Patients and caregivers | 71 | Findings on the focus of care, the model of care, and complementarity between teams involved. Role perception influenced by being in the intervention group (other than control group). | Embedded qual study within an RCT. Some aspects may be trial specific but appears in alignment with similar work. |
| Hasegawa [58] | 2022 | Japan | Qualitative interviews and focus groups | Explore barriers and facilitators to integrating home based palliative care and oncology, from the perspective of HCPs. | Cancer hospitals and at home palliative care services | Healthcare professionals (nurses, physicians, social workers) in palliative care or medical oncology. | 27 | Professional relationships and palliative care experience/ knowledge were facilitators because they enabled an understanding of what was being offered. Lack of referral criteria and unclear eligibility alongside treatment were barriers. | Data collection within one region of Japan, limited detail on the services themselves, but findings and recommendations are similar to those made elsewhere. |
| Hayden [59] | 2022 | Ireland | Qualitative interviews | Examine attitudes and perceptions on choice of care in event of terminal illness from the perspective of people who have cared for a loved one with terminal cancer | Recruitment via social media and with known contacts | Bereaved caregivers | 10 | Argues in favour for integration of palliative care in terminal cancer to reduce burden on caregivers. | Participants sampled via the existing network of the authors and social media so may not have representative experiences. |
| Henson [60] | 2016 | UK | Qualitative interviews | Explore decisions of advanced cancer patients and caregivers on their decision to attend the Emergency Department (ED) | University teaching hospital in South-East London | Patients (18) and caregivers (6) | 24 | Explains influences on the decision to attend ED in advanced cancer. Anxiety on symptoms and not feeling able to cope at home can motivate ED attendance - so addressing these areas could help. Earlier integration / more contact with community PC and GP could reduce the perception as the hospital being the most trusted option or only available place for help in an urgent situation. | Participants were based in London so they might have perceived ED as more straightforward to access than those in rural areas. |
| Hoek [61] | 2022 | Netherlands | Qualitative interviews | Explore GP perspectives on their role in shared decision making (SDM) in advanced cancer | General practitioners in a mixture of group / solo practices | Healthcare professionals (GPs with 4-30 years’ experience - mean 17.4). | 15 | GP’s role and requirements for shared decision making include collaboration with oncologist, information about disease and treatment, time, trusting relationship with patient, patient-centred communication. GPs perceived SDM to be responsibility of oncologist, but that they facilitated the process. | Selection of case studies discussed might have influenced findings / limited generalisability. GPs were mostly urban with an interest in palliative care. |
| Hojjat-Assari [62] | 2022 | Iran | Qualitative interviews and a focus group | Explain HCPs perception of the integration of palliative home care into the primary care system. | Professionals primarily involved in home care | Healthcare professionals (oncologists, palliative care specialists, general practitioners, nurses, psychologists, social workers, palliative home care system experts). | 21 | Health system structure provides opportunity for integration, including information sharing and practice of reverse referral from specialists to family physicians. Clarification of roles and investment in infrastructure are necessary to overcome workforce limitations. | There are similarities between the described healthcare system and the UK but there may be significant differences not discussed here. |
| Horlait [63] | 2016 | Belgium | Qualitative interviews | Identify barriers experienced by medical oncologists in introducing palliative care | Academic and non-academic hospitals in Flanders | Healthcare professionals (medical oncologists) | 15 | Shortcomings in medical education do not prepare oncologists for the communication skills required. | Invited 82 medical oncologists, but only 15 responded. Those who did not respond might experience greater or different barriers than those reported here. |
| Hoverman [64] | 2020 | USA | Retrospective analysis of patient characteristics, outcomes, and costs | Measure and characterise total cost of care for patients who died while managed in US oncology. Compared total costs of care for those receiving <3 days hospice care with those receiving >3 | Centres for Medicare and Medicaid services | Patients (quant) | 7329 | Information on EOL costs for people who received cancer treatment within the last 6 months of life. Those receiving hospice care for more than 3 days had lower cost in the last 30 days (than those with no hospice or <3 days). Patients who were black or who had lymphoma had lower use of hospice. | Only observational, doesn't tell us how to achieve integration, but large sample and useful discussion. |
| Howell [65] | 2011 | Canada | Retrospective analysis of clinical and administrative data | Examine predictors for home care use and home death within "gold standard" palliative home care programme | Patients with cancer (number of types including lung, GI, breast, genitourinary, skin, haematological, prostate, head and neck, pancreas, brain). | Patients (quant) | 418 | Reports that access to palliative home care has potential to enable home death and shift care resource use away from acute care. Identifies household income as an influence on home death. | Uses postcode proxy for household income. Does not include measure of care experience or quality of life/death. |
| Huo [66] | 2020 | USA | Retrospective analysis of patient characteristics, outcomes, and costs | Examine utilisation of speciality palliative care over a 14-year period, including association of specialist palliative care (SPC) with demographics and survival. | Medicare beneficiaries, across settings. | Patients (quant) | 79,252 | Timely SPC utilisation demonstrated to be cost-saving for metastatic non-small-cell lung cancer. Paper reports both longer survival and lower costs in outpatient PC. | Patients who had their first contact with SPC in the outpatient setting appeared to have better outcomes - but this is observational and study design did not account for subsequent service settings. |
| Johansen [67] | 2018 | Norway | Qualitative focus group and interviews | Explore experiences of collaboration between GPs and oncology nurses | Northern Norway (involved 25 out of the 87 local authorities in the region) | Healthcare professionals (15 cancer nurses, 15 district nurses, 17 GPs, 5 allied healthcare professionals) | 52 | Explains the value of complementary competencies, but found barriers related to organisation, funding, remuneration, limited time, different cultures/strategies for learning. | Two (or more) publications from this data collection (2015-2016). Broad sampling strategy, authors suggest might be generalisable to other rural areas. |
| Johansen [68] | 2022 | Norway | Qualitative focus groups (10) and interviews (6) | Explore experiences of local and regional collaboration in meeting the needs of patients who need palliative care. | Staff from a mixture of rural settings | Healthcare professionals - 15 district nurses, 15 oncology nurses, 17 general practitioners, 5 physiotherapists/occupational therapists | 52 | Explores "talking together" as an optimal form of collaboration between primary care and specialist palliative care and the factors promoting or inhibiting collaboration between disciplines. | Useful detail. Data collected in 2016-16. Rural settings, primary care providers with long-term relationships with patients. |
| Johnston [69] | 2013 | UK (Scotland) | Mixed methods | Evaluate feasibility of effectiveness trial of early access to palliative care | Hospital based lung cancer clinic | Patients (lung cancer) | 3 | Palliative consultant attended the oncology clinic for the study period. Suggests valuable aspects of the intervention in information-provision, which helped in planning for the future and being aware of what support was available. | Recruitment was low, partly due to participants being unwilling to travel across a rural area for the extra clinic visit. |
| Kaye [70] | 2018 | USA | Retrospective cost analysis | Examine association between system-level integration and phase-specific costs in ten most common cancer types | National data set, across settings | Patients (quant) | 428,300 | Compared integrated to less integrated systems, found lower costs in some types of cancer. | Used a binary measure of integration that might not be relevant to other countries. Comments only on costs - not on composition of expenditures, or on outcomes for patient or caregiver - so not enough evidence to interpret the overall null result. |
| Keim-Malpass [71] | 2015 | USA | Qualitative interviews | Identify barriers to accessing palliative care for cancer patients in a rural catchment area | NCI-designated academic cancer centre with a rural catchment area | Healthcare professionals (nurses, nurse practitioners, cancer physicians, primary care physicians, administrators, allied health professionals). | 42 | Fragmentation of electronic systems makes it more difficult to coordinate care. Important that scheduling is considered in rural areas. Highlights other opportunities to enhance integration of PC from a process, education, and systems perspective. | Multistakeholder approach, including community professionals, but focused on a single catchment area. |
| Kitta [72] | 2021 | Austria | Qualitative interviews | Examine how patients perceive end-of-life discussions and their transition to palliative care after treatment | Palliative care unit at a university hospital, patients admitted or transferred from oncologists | Patients | 12 | Emphasises how end-of-life conversations were vague and brief, impeding opportunities to ask questions. Patients ambivalent in their desire for information, coping or defence mechanisms are relevant. Coming to terms with their situation is a process, not a one-off event. | Ethical concerns around speaking to people at most distress, so not the full picture, but useful insights from the participants that were involved. Single centre study. |
| Kleiner [73] | 2021 | Switzerland | Content analysis of EPC consultations | Understand the content of EPC consultations (physician-patient) as part of a trial | University hospital of Bern | Consultations between patients (10) and HCPs (4) | 10 consultations | EPC consultations include a variety of topics, establishing rapport, developing patient relationship, providing reassurance and positive emotional talk. Recommends predefined structures to guarantee important aspects are addressed. The consultations were a one-off meeting, and patients rarely asked their own questions. | Detailed investigation using RIAS framework of a small number of consultations. |
| Krause [74] | 2024 | South Africa | Mixed methods including qualitative interviews | To understand palliative care integration in hospitals and how it can be achieved. | Academic teaching hospital | Hospital management staff (5 doctor executives, 1 nursing manager, 1 social worker manager) | 7 (qualitative interviews) | The service was instigated by a small number of 'champions' with support from the university. Findings show that alignment of leadership, governance and education did not follow-on from this. Foregrounding commitment to PC excellence is needed – beyond just a few individuals. | Mixed methods study of one vertical integration service at an academic teaching hospital, small sample of interviews, possible selection bias so that unfavourable viewpoints less likely to be sampled. |
| Krause [75] | 2024 | South Africa | Mixed methods including qualitative interviews | To understand contextual factors influencing palliative care integration in hospitals. | Academic teaching hospital | Hospital management staff (5 doctor executives, 1 nursing manager, 1 social worker manager) | 7 (qualitative interviews) | Provides detail on interactions between micro, meso, and macro in the functional and normative integration of PC. | Mixed methods study of one vertical integration service at an academic teaching hospital, small sample of interviews, possible selection bias so that unfavourable viewpoints less likely to be sampled. |
| Kremenova [76] | 2022 | Czech Republic | Retrospective cohort study | Determine difference in costs and hospital resource use with and without support of the hospital palliative care team. | University hospital | Patients who died in the hospital between January 2019 and April 2020 (81.2% cancer) | 426 | Daily costs were three times lower in group receiving palliative care. Significant differences between groups in the identification of end-of-life phase, time spent in ICU, and length of hospitalisation episode. | Included terminal (EOL) hospitalisation costs only (no earlier hospitalisations or costs within other settings). |
| Kruser [77] | 2020 | USA | Qualitative focus groups (conducted during ASCO annual meeting.) | Characterise medical oncologists' perceptions about palliative care referral | Medical oncologists primarily practicing in academic settings and in Midwestern US. | Healthcare professionals (medical oncologists) | 31 | Identifies that the integration of palliative care is impeded by medical oncologists' perceptions of the aims of palliative care (as counter to treatment, or not informed enough about treatments) and their sense of authority over their patients (/aversion to conflicting messages). | Primarily university-based practice, possible that barriers identified would be greater or different in less academic settings. |
| Kubendran [78] | 2021 | USA | Retrospective analysis of patient data and resource use | Examine trends and factors associated with inpatient palliative care use in primary brain malignancies | Nationwide inpatient sample | Patients (quant) | 510,238 | Palliative care associated with decreased cost of admission. Identified factors associated with increased odds of receiving palliative care. | Descriptive study of costs and resource use, does not comment on how the groups were formed (why some people received PC) |
| Le [79] | 2014 | Australia | Qualitative focus groups (22 participants) and interviews (6 participants) | Explore lung cancer clinicians’ perceptions of early palliative care | Three metropolitan university hospitals in Melbourne | Healthcare professionals (medical oncologists, respiratory physicians, radiation oncologists, lung nurse coordinators, social workers, nuclear medicine physicians, thoracic surgeon, respiratory registrar, radiation oncologists, occupational therapist, oncology nurse). | 28 | Four themes that influence engagement with palliative care - perceived competence of the palliative care service, care coordination to mitigate fragmentation, ease of referrals including responsiveness and a physical presence, anticipated patient/family reactions. | Includes several disciplines but is limited to urban university hospitals. |
| LeBlanc [80] | 2015 | USA | Mixed methods using surveys and interviews | Understand and contrast perceptions of palliative care among oncologists | Three academic cancer centres in the US with well-established palliative care clinics | Healthcare professionals (haematologists = 23, solid tumour oncologists = 43) | 66 | Contrasts referral patterns for solid tumour and haematological oncologists, reports philosophical and practical barriers to referral that differ between groups. Haematology appears to be behind other areas in adopting a modern understanding of palliative care (as a partner in co-management, rather than antithesis to treatment). | Likely response bias, and not clear how academic setting influenced attitudes reported. Haematologists in this sample were older than the oncologists, which may have also influenced their perceptions of PC. |
| Lee [81] | 2022 | Australia | Qualitative interviews | Explore experience of caregivers of people with mesothelioma towards end-of-life | Bereaved caregivers, mostly partners, urban, recruited via disease-specific community organisations | Caregivers | 14 | Challenges arising from unmet informational needs that limited shared decision making and preparations for end of life. Care coordination may help reduce the burden on caregivers. | Specific cancer group but might be common issues regarding impact of unmet informational needs. |
| Liu [82] | 2022 | China | Randomised controlled trial | Assess interdisciplinary hospice care provided to terminal geriatric cancer patients | Ganzhou Cancer Hospital | Patients (quant) | 166 | Cost of drugs lower in the intervention group. Satisfaction and cooperation reported to be higher. Comments on how collaborative team model leads to positive outcomes. | Single site and fairly small sample but used randomisation and demonstrated results. Limited information on patient participants. |
| Lundeby [83] | 2020 | Norway | Qualitative focus groups and interviews | Explore physicians' and nurses' challenges of integrated oncology and palliative care | Recruitment from Oslo university hospital, 1 of the 12 oncology departments in PALLiON project | Healthcare professionals (5 oncologists, 5 palliative care physicians, 7 oncology residents, 6 palliative care nurses, 12 oncology nurses) | 35 | Specifies cultural and organisational factors, including how HCPs perceive themselves and other HCPs, and few formalised arenas in which collaboration can take place. | Single-centre study, used to inform development of PALLiON implementation. Focus group setting might obscure reflections on the shortcomings of own practice (i.e., participants noted as discussing the perceived inadequacies of other professionals rather than their own learning needs). |
| Lundeby [84] | 2023 | Norway | Qualitative focus groups, needs questionnaire, follow-up survey evaluation of the educational intervention | Develop, implement and evaluate training programme in communication skills for integrated oncology and palliative care | Oslo University Hospital (primarily) | Healthcare professionals (oncology residents, oncologists, nurses in oncology and palliative care, physicians) | 254 | Reports several lessons learnt regarding the implementation of a training programme. | Reports on one aspect of the PALLiON intervention as a whole - focus on the educational programme. Not designed to capture impact on practice. |
| Lundereng [85] | 2020 | Norway | Qualitative interviews | Explore nurse's experiences on discharge collaboration when patients go home from hospital. | Nurses from oncology wards and home care services | Healthcare professionals (5 hospital nurses, 5 home care nurses) | 10 | Reports distrust, misunderstandings, and misconceptions of each other that led to inefficient communication and poorly planned discharges. Better structured collaboration could clarity roles and build trust. Information sharing was a mixture of telephone and electronic. | Focus on one region of Norway but the general implications of the findings seem to resound with what is found elsewhere e.g., different ways of working between organisations brings uncertainty and contributes to mistrust. |
| Maessen [86] | 2024 | Switzerland | Cost-consequence analysis alongside multicentre trial | Determine impact on resource use and costs of health care in last month of life from an early PC intervention | Multicentre trial of early PC intervention (one appointment with follow-up visits if requested) | Patients with advanced cancer in an intervention study who died within the study period | 58 | Median/absolute cost was lower in intervention group on average (600 CHF - increasing to 1204 CHF in per-protocol analyses) but no significant overall difference identified between groups in costs. Lower emergency department use and fewer deaths in hospital in intervention group. | Alongside an intervention trial. Participants included only where bereaved caregivers had responded to study invitation (58 of 150). Focused analysis only on last month of life and excluded any ongoing treatment that had started more than one month before death. Control group could access the intervention resource on demand. |
| Mashiro [87] | 2023 | Japan | Qualitative interviews | Identify barriers to collaboration from the perspectives of professionals supporting cancer patients. | Hospitals and community care institutions | Healthcare and long-term care providers (24 doctors, 17 nurses, 8 social workers, 8 physio/occupational therapists, 8 care managers, 5 public health nurses, 5 dentists, 2 care workers, 4 others inc adminstrative workers) | 88 | Adds detail on how collaboration across settings requires opportunities to establish relationships, share information, and coordinate roles. | One medical region in Japan, using snowball sampling from the researcher's network. Acknowledges data collection occurred before Covid-19 pandemic which will have a relevant impact on the findings. |
| Mayland [88] | 2021 | UK (England) | Qualitative interviews | Report on experiences from advanced head and neck cancer on integrating specialist palliative care (mixed perspectives) | Regional HNC MDT meetings, outpatient clinics, and SPC services in hospitals and hospices | HCPs (8 - H&NC surgery, oncology, specialist PC, GP, community nursing, H&NC CNS), patients (9) and caregivers (4) | 21 | Unpredictable disease trajectory makes treatment outcomes less certain so context might differ from other forms of cancer - clear and effective communication of the goals of care are required. | Participants only included who were identified by clinical team as being aware of their diagnosis - aspect of gatekeeping that meant study didn’t capture those who were not already tapped into SPC. |
| McCaffrey [89] | 2012 | Australia | Prospective economic evaluation | Evaluate cost-effectiveness of a home-based palliative care intervention. | PEACH home-based intervention | Advanced cancer patient who had complex or unstable care needs | 32 | Small pilot study indicating potential for home-based palliative care to reduce time spent in hospital and the costs of inpatient care, but not necessarily shift the place of death. | Trial follow-up was only 28 days, at which time 68% of participants had died. Possible for benefits to continue to accrue beyond the 28 days study period. |
| McCaughan [90] | 2019 | UK | Qualitative interviews | Explore haematology nurses’ perspectives of patient care and death | Sampled via UK Haematology Malignancy Research Network, most participants worked in haematology wards. | HCPs - haematology specialist nurses, either ward/area managers or clinical nurse specialists. | 8 | Continuity of care with the ward nurses is valuable and their relationships with patients might allow them to detect subtle changes relevant to prognostication. Recommends earlier, frank conversations on realistic outcomes. Better communication between secondary/primary care and increase in out-of-hours support could help to reduce chance of hospital death (although this is unavoidable/preferable in some situations). | The study has a narrow focus, but its findings cover a range of areas and appear to have broad applicability. Some nurses were interested in participating but unable to do so due to workload commitments. Their perspective has not been included. This matters because the nurses who are included seemed to value their patient relationships and didn’t want to 'let go' of patients to separate palliative care teams, and it might be that those with higher workload challenges are keener to do so. |
| McPherson [91] | 2023 | Norway | Qualitative interviews | Explore haematology nurses’ experiences of palliative care trajectories | Local, regional, and national hospitals in Norway | Healthcare professionals (haematology nurses on inpatient wards) | 12 | Reports that a focus on cure delays integration of PC, dialogue with patients facilitates PC, and there is a need for enhanced interdisciplinary understanding. | Mostly experienced nurses, recruited by the ward managers, with training in palliative care. |
| Mensah [92] | 2023 | Ghana | Qualitative interviews | Explore expectations of palliative care and barriers to palliative care utilisation from the perspective of people living with cancer. | Oncology outpatient department (teaching hospital) | Cancer patients who have been receiving treatment for more than 6 months (range of cancer types) | 15 | Insight into perceptions of palliative care from a resource-constrained setting. | Limited information provided on the interview participants. Most of the interviews were conducted in Twi (local language). Sampled from one of three hospitals providing palliative care in Ghana |
| Mollica [93] | 2018 | USA | Qualitative interviews | Gather perspectives on how palliative care is delivered in the context of clinical trials | Two multidisciplinary clinical trial teams at a US teaching hospital | Healthcare professionals (principal investigator/attending physician, oncology fellow, research nurse, physician assistant, nurse practitioner, social worker, clinical nurses, palliative care fellows, chaplain, pharmacist). | 19 | Varied personal meanings of PC and conflation of PC with EOL impede perceived appropriateness and timing of PC delivery. Clinical trial context means focus on curative-intent is magnified. | Looks at two teams within one institution, although similar to findings that are reported elsewhere. |
| Monnery [94] | 2023 | UK | Prospective observation of clinical outcomes (n=4594) | Determine the impact of enhanced supportive care on symptom burden and secondary care use for patients with incurable (but treatable) cancer | Enhanced supportive care centres in England - mixture of outpatient, ambulatory, inpatient, domiciliary service models. | Patients (quant, mixed tumour types) | 4594 | Reports varied service models and staffing structures across 8 centres. In general, IPOS scores improved and there was a reduction in secondary care usage, but these outcomes varied by tumour type. | IPOS scores provided by only 4 centres, not given detail on (n) by tumour type for clinical outcomes. Unclear how research locations were selected. Comparison data was pre-covid which might impact the validity of these results. |
| Montiel [95] | 2023 | Canada | Qualitative interviews and focus groups | Identify barriers and facilitators to supportive care access among men with cancer. | Recruited via hospitals, universities, community organisations and social media | Adult men with cancer (aged 26-82) | 31 | Considers how sociocultural norms, awareness and trust influence the perception of services, argues in favour for tailoring services and for informed decision-making. | Reporting is unclear on stage of illness - approx. 60% of participants were 'post-treatment' or had no treatment available. Largely urban and white sample, high proportion with university or college education. |
| Morikawa [96] | 2016 | Japan | Qualitative interviews | Assess haematologists and palliative care specialists’ perception on the roles and barriers to the hospital based palliative care team (HPCT) | Hospital providing haematology medicine or home palliative care. | Healthcare professionals (11 haematologists and 10 palliative care specialists). | 21 | Specifies barriers to collaboration, including timing, negative perception of the team, lack of two-way communication, not perceiving the need to refer to HPCT, lack of proactive suggestions for PC involvement. | Details contextual challenges to the relationships between professions. Not clear if this one or across multiple locations. |
| Ndiok [97] | 2021 | Nigeria | Qualitative interviews and focus groups | Identify barriers to and potential benefits of integrated palliative care for cancer patients in tertiary hospitals | Two tertiary health institutions | Healthcare professionals (19 nurse managers, 2 nursing directors, 2 medical chief/chairman) | 23 | Benefits could be to the hospital and to patients/family. Barriers include lack of local policy, cultural wariness of hospital, attitudes of HCPs, patients not attending appointments, and financial implications (especially requirement for investment to resource teams). | Captures the perspectives of senior staff at two teaching hospitals - may be different views or barriers in other settings. |
| Ndiok [98] | 2019 | Nigeria | Qualitative interviews and focus groups | Examine home visiting for follow-up after hospitalisation as a strategy to improve palliative care. | Two tertiary health institutions | HCPs (19 nurse managers) and patients (11 cancer inpatients) | 30 | Identified benefits of home visits for meeting informational needs and for overcoming the barriers to patient attendance at hospital. Need resources for home visits to be implemented: including policies, staffing, and funding. | Key benefit was having time to explain and answer questions, which enabled involvement of patients in SDM and for informal caregivers to be involved in care. This appears to be transferable across different settings. |
| Okyere [99] | 2023 | Ghana | Qualitative interviews | Explore barriers to successful integration of PC in cancer | Korle Bu Teaching Hospital | HCPs (7: geriatric nurse, community nurse, palliative care nurse specialist, pharmacist, family physician, general nurse, clinical psychologist, social worker), patients (4) and caregivers (2) | 13 | Reports barriers to integration related to misunderstandings of palliative care, lack of service availability, financial resources, access to medication, and low staff strength. Suggests that leadership not prioritising PC has impact on staff wellbeing. | Single site study, palliative care unit established in recent years. |
| Olafsdottir [100] | 2018 | Iceland | Qualitative interviews | Explore patient and family experiences of engaging in a structured ACP discussion facilitated by palliative care nurses with accompanying booklet 'Thinking ahead - what's important to me'. | University hospital palliative care team | Patients (recently diagnosed with advanced lung cancer and referred to palliative support service) | 7 | Findings suggest the ACP discussion was appropriate and helpful, but also difficult and sensitive. A routine approach with flexible structure helped to normalise the process. Booklet may be helpful in instigating discussions with family members. | Small pilot study, some hints about 'what works', focus on experienced nurse facilitator, using but not relying on informative booklet. |
| Payne [101] | 2017 | UK | Qualitative interviews | Investigate hospice integration with local healthcare providers using the supplement/support/supplant model. | Hospices in the North of England who had multidisciplinary collaboration with other organisations. | HCPs (23), patients (34) and family caregivers (13). Most (14) HCPs were qualified nurses from community, day hospice, and inpatient settings. There were also 5 physicians (3 GPs, 2 palliative medicine consultants), 1 occupational therapist, 1 physiotherapist, 1 social worker, 1 chaplain. | 70 | The conceptual model of hospice integration being supplement/support/supplant needs further development, as the findings suggest a blurring of these boundaries. Patient satisfaction with care was associated with information sharing between providers and continuity of care relationships. Funding constraints and unclear responsibilities lead to issues with service delivery. | Includes four hospices in northern England that worked collaboratively with other organisations, so not a comprehensive overview. Data collected from July 2014 to October 2015. Useful insights although doesn't explain how these ways of working were established/ implemented. |
| Perry [102] | 2021 | USA | Intervention development and pilot testing | Develop and evaluate an intervention explaining palliative care to patients and families, tailored to different levels of health literacy | Tulane cancer centre | 21 stakeholders in intervention development (HCPs, patients and caregivers), pilot tested on 10 patients. | 31 | Tailoring information about palliative care for different levels of health literacy might be effective in improving patient readiness for palliative care, addressing misconceptions. Could also promote conversations about primary palliative care between patient and oncologist. Format was video alongside written handouts. | Small pilot sample – useful example of informational intervention. |
| Porto [103] | 2012 | Brazil | Qualitative observations and focus groups | Study interdisciplinary practice in oncology health team | Oncology interdisciplinary home hospitalization program | Healthcare professionals (nurse, nursing technician, nutritionist, team coordinator, physician, surgeon, social worker, theologian, administrative aid, graduate psychology student). | 9 | The interdisciplinary programme works to improve care quality and professionals’ satisfaction via the horizontality of power relations and shared learning within the team. | Details integrated interdisciplinary palliative care practice. Limited information on the programme itself. Seems to tie together some of the nuggets from elsewhere on optimum team functioning. |
| Preisler [104] | 2019 | Germany | Qualitative interviews and focus groups | Identify information needs and contributors to unmet information needs of caregivers during patient hospital stay | Oncology unit of university hospital in Berlin | Caregivers | 17 | Identifies unmet information needs including on what to expect from treatment, what to expect from the healthcare system, and how to manage when they get home. Uncertainty and stress made it harder to ask questions. | Single site study. Included only native German speakers. |
| Preisler [105] | 2018 | Germany | Qualitative interviews | Investigate if needs are phase-specific or patient-centred, considering perceptions throughout the cancer trajectory | Hospital oncology department (inpatients and outpatients) | Patients (11) and caregivers (9) | 20 | Subjective illness understandings influence experiences, requirements, and preferences of EPC. Described healthcare access to be based on chance - recommended structures are improved so that success is no longer relying on being lucky. | Single site study. Only included native German speakers. Possible that participants might have been selective in their criticism. |
| Prod'homme [106] | 2018 | France and Belgium | Qualitative interviews | Determine haematologists’ barriers to end of life discussions | Four haematology units in two private university hospitals and two (public) general hospitals | Healthcare professionals (haematologists with 5-25 years’ experience) | 10 | Haematologists fear that end-of-life discussions may undermine patient trust in them and in treatment, so they may perceive a moral dilemma in opening up to their patient's end of life wishes. | Almost half of participants had specific training in medical ethics which might be relevant to their participation / experience. |
| Rao [107] | 2022 | India | Participatory action research (PAR) | Develop, implement and evaluate palliative care capacity building programme | 31 cancer treatment institutes | Healthcare professionals (mixture of doctors and nurses across three PAR cycles). | 73 | Emphasises the importance of change champions and reflective cycles in achieving action. Training helped to initiate partnerships between professionals. Organisational buy-in is relevant for capacity building and may also facilitate change beyond the organisation. | The paper focuses on the capacity building process. Notes that the programme was developed without the 'end user' i.e. patients and families. |
| Raunkiaer [108] | 2020 | Denmark | Qualitative focus groups and interviews | Consider the perspectives of HCPs involved in a randomised clinical trial (DOMUS intervention accelerating access to specialist PC) | RCT involved oncology department at Copenhagen University Hospital, regional specialist palliative care teams, and municipal home care nurses. | Healthcare professionals in the DOMUS RCT (7 oncology nurses, 3 oncology nurse leaders, 1 oncology doctor; 8 specialist palliative care nurses,1 nurse leader, 2 doctors; 12 home care nurses; 4 psychologists) | 38 | Findings show how the trial disrupted existing practice, had organisational significance, increased collaboration, and resulted in a broader range of patients being referred to PC. | Sample was predominantly nurses (31 total), research did not include social workers, who carried out many tasks in the intervention, and could not identify any of the GPs involved to explore their views. |
| Raunkiaer [109] | 2023 | Denmark | Qualitative interviews | Explore barriers and facilitators in organising palliative care, in relation to the process of cessation of antineoplastic treatment. | Oncology clinics, specialist palliative care teams and home care nursing, participating in the DOMUS RCT | Nurses (31) and physicians (3) | 34 | Managers and healthcare professionals explain processes related to delivering palliative care and identify opportunities for improvement. | Secondary report from Raunkiaer 2020 (data collection 2016-2017). Focuses on the trial of one intervention - findings seem to resonate with reports from elsewhere. |
| Ribi [110] | 2022 | Switzerland | Qualitative focus groups and Delphi survey | Explore decisional factors for the use of systemic anticancer treatment (SACT) in advanced cancer patients | Tertiary cancer centre, primary care, home care within the region | HCPs (Invited oncologists and senior clinical fellows at the tertiary cancer centre, general oncologists in the region, oncology nurses, palliative care nurses, home care nurses, and GPs. Of these, 17 physicians and 11 nurses took part) and advanced cancer patients (15) | 43 | Develops a SACT decisional framework integrating subjective patient factors, interpersonal factors, and palliative care issues in a decision process. | Acknowledges decisional processes may be influenced by local cultures and there is low likelihood of experience of poverty in the participants, so framework would need to be adapted. Used focus groups and Delphi - may have had different findings in smaller groups/interviews (more emotive). |
| Roberson [111] | 2023 | USA | Qualitative interviews | Explore patient perspectives on the delivery of supportive and palliative care services | Project Life' virtual wellness community | Women with metastatic breast cancer | 36 | Centres the patient voice in 're-imagining' cancer care, particularly emphasizes the need for tailored information sharing, shared decision-making, and long-term planning. | Study conducted within an online wellness community – possibly more likely to educated/empowered patients. Reports no info on socioeconomic status or home environments, but range of institutions, ages and 77.8% white ethnicity. |
| Rohrmoser [112] | 2017 | Germany | Qualitative focus groups | Explore perspectives of HCPs on desirable standards of support for patients and caregivers throughout the cancer trajectory, in relation to early palliative/supportive care | Oncology department at a university hospital | Healthcare professionals - oncologists | 9 | Suggests support needs occur throughout trajectory. Many relate to having a sense of control. Continued relationships / consistent information builds trust and reduces uncertainty. Recommends regular needs screening (especially at times of change) and team meeting with multiple professions to better identify support needs. | Small sample and focus group format could have influenced findings. Oncologists’ perspectives only. |
| Rossi [113] | 2021 | Italy | Retrospective analysis | Investigate costs of radiotherapy in the last month of life, considering those that did not start, did not complete, or did complete their course of RT. | Radiotherapy unit of specialist cancer centre (which also has a palliative care unit) | Patients (quant) most commonly lung, but also gastrointestinal, urology, breast, head and neck. | 160 | Concludes that improved prognostication and integration of palliative care could reduce overall costs by making inappropriate invasive treatment less likely in the last month of life. | Retrospective observational study, single centre, no data on treatment intent or out of pocket costs. |
| Sadang [114] | 2023 | USA | Qualitative survey | Consider barriers, facilitators and impact of telehealth for early integrated palliative care (EIPC) from the perspective of providers | Multisite randomised controlled trial comparing EIPC via video or in-person. | Palliative care and oncology clinicians who participated in an RCT of EIPC | 48 | Participants agreed that telehealth could increase access to EIPC, but mixed views on whether the telehealth format would be accepted by patients or cost-saving for the healthcare system. Some patients more likely to dislike or avoid technology. | Trial context confers extra resources that are not available in usual practice (study coordinators, devices to loan to patients). Qualitative survey questions, all academic institutions, and response rate at 51.1% suggests there might be respondent bias. |
| Satija [115] | 2022 | India | Qualitative interviews | Evaluate experiences of teams in implementing quality improvement (QI) methods to improve access and quality of palliative care | Cohort of sites - 4 tertiary hospitals, 3 palliative care centres | Healthcare professionals (8 organisational leaders, 12 clinical leaders, 24 team members). | 44 | QI was able to engage and empower multiple stakeholders in a systematic process to identity opportunities for the improvement of palliative and cancer care. | Details use of QI tools in a resource constrained setting. Useful on this aspect, doesn't provide detail of QI outcomes or the initiatives themselves. |
| Schenker [116] | 2018 | USA | Mixed methods | Assess a model of early palliative care for advanced pancreatic cancer, considering feasibility, acceptability, and perceived effectiveness. | National Cancer Institute-designated comprehensive cancer centre in Pennsylvania | HCPs (oncologists and palliative care = 7), patients and caregivers (30 pairs) | 67 | Highlights the practical burdens of additional hospital visits and the need to tailor frequency and content of palliative care input. HCPs noted that email contact was insufficient - rather, in-person communication between oncologists and palliative care physicians could improve patient care. | Significant proportion of those approached declined to participate. Single centre research with predominantly white patients that have high educational and socioeconomic status. |
| Schenker [117] | 2014 | USA | Qualitative interviews | Explore factors that influence oncologist referrals to outpatient palliative care | Three academic cancer centres with well-established outpatient palliative care clinics. | Healthcare professionals (oncologists - both haematology and solid tumours). | 74 | Service availability and awareness of the service are both necessary for referrals. Increasing service availability alone might be insufficient to improve access because generalist/specialist roles are poorly defined, and modern definitions of palliative care have not penetrated oncology. | Multiple sites with oncologists from a range of specialities. Doesn't acknowledge that 2 or the 3 PC clinics in question are not called 'palliative care'. |
| Schifferdecker [118] | 2023 | USA | Mixed methods: interviews, observations, document analysis. | Compares organisational, sociocultural, and clinical factors that support or hinder PC integration | Three US cancer centres (organisational case study, primarily inpatient and outpatient care, ED mentioned) | Clinicians (62) and leaders (27), 22 days observation | 89 interviews | Emphasizes significance of social norms for integration. Team-based orientation is facilitated by organisational policies, sufficient staffing, and perceived agency to discuss EOL. Emphasises that structural change is insufficient without also giving attention to favourable social norms for palliative care. | Useful contribution from analysis of three sites. Only small indicators for intervention but does have a partial theory of 'what works'. |
| Senior [119] | 2010 | UK | Qualitative focus groups | Discuss with community nurses how palliative care of Scotland islanders may best be met | Community nursing on the Scottish Islands | Healthcare professionals (community nurses) | 10 | Collaborative model of care could be useful, involving a shift in caseload responsibility and improved education opportunities to empower nurses and home care staff and enable better continuity of care. Nurses fear burgeoning workload, need time to learn and to share this knowledge. | Reports (only) community nurses’ perceptions of a best model of palliative care, suggested a shift in caseload responsibility to improve continuity of care provider and communication between HCPs. |
| Seow [120] | 2021 | Canada | Retrospective population-based cohort study | Investigate the impact of early palliative care | Hospital or community palliative care | Cancer decedents (most commonly lung, colorectal, breast and prostate) | 79,648 | Early palliative care appears to be cost saving (more than 6 months before death). | Included palliative care across different settings, used 'real-world' data. Matched for key demographics but not all possibly relevant variables. |
| Siegle [121] | 2022 | Germany | Mixed methods | Evaluate "Milestone Communication Approach" intervention, to understand why ACP was infrequently discussed | Outpatient oncology department in comprehensive cancer centre at a university hospital | Patients and caregivers, plus content analysis of consultations | 171 | Patient readiness for prognostic information varies. Recommendations for clinicians breaking bad news: to name their predicament in the clinical encounter, i.e. of being responsible for giving information while respecting patient wishes to avoid it. | Related to an RCT within one university hospital. |
| Siler [122] | 2018 | USA | Qualitative interviews and focus groups | Explore palliative care and oncology clinicians’ perspectives on improving QOL in community-based settings | Three outpatient clinics in the Southwestern US | Healthcare professionals (both palliative care and oncology: physicians, nurses, social workers, chaplains, administrators) | 19 | Staff outside of the SPC team need education to understand the role and remit of SPC. Coordination of care was seen as including social work and support groups but constrained by lack of availability of these services. | Included three locations, but relevant organisational aspects are only briefly mentioned (particularly around time constraints). |
| Skorpen [123] | 2020 | Norway | Qualitative focus groups | Explore how nurses experience compassionate care for patients and caregivers - conceptualises compassion as an action | Primary care and nursing home nurses | Healthcare professionals (nurses) | 21 | Compassionate care requires dialogue, relationship building, and information provision. Hospital based clinicians should be responsible for giving information about the value of early involvement of nurses at home. Barriers to compassionate care included lack of time and poor collaboration with physicians resulting in inadequate symptom relief due to lack of access to medication (e.g., out-of-hours). | Focused study with broad implications - no word for compassion in Norway so they have detailed what it means. Suggestions from other studies that findings agree with work elsewhere. |
| Sommer [124] | 2021 | Switzerland | Qualitative focus groups and interviews | Develop, pilot, and evaluate an intervention to train primary care physicians on communication skills and palliative care competencies | Primary care in French-speaking Switzerland | HCPs (primary care medical teachers) | 8 | HCPs found the intervention acceptable, but patient recruitment was a challenge. HCPs reported a change in how they saw their role within palliative care. | Suggests that having a "plan B" if treatment works could be reassuring for patients. However, this study doesn’t capture the patient's perspective. |
| Spelten [125] | 2021 | Australia | Qualitative interviews and focus groups | Identify components of the community-based palliative care service that would require adaptability to ensure sustainability of the service. | Community palliative and end of life service in a rural area 0 | Healthcare professionals in the PEOL team (6) and externally (10), plus family members (9) (Paper also includes quant summary of patient demographics and contacts with service - 121 individuals). | 25 | Patient uniqueness, workforce issues, collaboration with other services, and symptom/pain management all require adaptability of the service to maintain sustainability. Details ways of working between different providers. Delays in obtaining medications was a key reason for hospitalisation. | The service was started as a pilot and has since grown. Fairly small sample. Some issues reported might be specific to Australian legal context. |
| Stern [126] | 2012 | Canada | Qualitative interviews | Explore perceptions and experiences of telehealth as part of an RCT (which took place 2004-2006). | Community based palliative care team connected to a large teaching hospital | HCPs (multiple disciplines - 14), patients and caregivers (5 patient/carer dyads, 7 bereaved caregivers) | 31 | Telehealth was valued for the ready access to professionals, and video links were reassuring. Concerns include lack of integration with other community services and the portability/reliability of the technology involved. | Older data and less able to include those with negative experiences of the intervention. |
| Stewart [127] | 2022 | UK | Retrospective analysis of secondary care use | Describe and evaluate enhanced supportive care service | Royal Sussex County Hospital (Brighton) | Cancer patients presenting in the Emergency Department | 260 | Indicates that the service was feasible and cost-effective. Highlights the potential benefit for supportive/palliative care for cancer patients within emergency settings. | Real-world' data, using a national dataset as comparator. Unclear if this might impact on validity of conclusions. Overall useful paper. |
| Sullivan [128] | 2023 | USA | Qualitative interviews | Understand barriers and facilitators to palliative care integration in lung cancer from the perspectives of palliative care and lung cancer clinicians | 21 medical centres or hospitals, mostly urban, across 19 states. | Healthcare professionals (21 physicians, 2 nurse practitioners) | 23 | Trusting relationships between HCPs are crucial to integration. Relationships are fostered through joint management of patients and encouraged by organisational leadership and financial support, co-location of services. Enables 'warm introduction' to PC. | Broad sampling strategy and good detail on relationships within integration. Fairly small sample size considering the high number of settings. |
| Tartaglione [129] | 2018 | USA | Qualitative interviews | Assess oncologists and palliative care clinicians’ perceptions about integrating oncology and palliative care using a nurse-delivered intervention | 6 medical centres (Veterans Affairs) over large geographical area | Healthcare professionals (7 oncologists, 12 palliative care clinicians) | 19 | Visibility of the nurse, establishing strong relationships, and having a clear and credible role could allow this model to overcome perceived differences and mistrust between the two professions | Nurse-led integrated palliative care model appears promising in overcoming the issues identified elsewhere.  Participants could have been those most willing to make integration work and possible that Veterans Affairs settings vary from others. |
| Tate [130] | 2022 | USA | Ethnographic observations | Examine the way decisions are made about treatment in the oncologist-patient interaction | Cancer clinics in southwest US | 90 interactions, between 5 oncologists and 82 patients | 87 | Unpacks 'the treatment imperative' that drives interactional hesitancy among doctors towards treatment cessation or palliative care. Suggests cultural lag despite large-scale policy changes. | Detailed conversation analysis on one piece of the puzzle - useful in that but limited information on variation between research locations, only included 5 oncologists. |
| Taylor [131] | 2017 | UK | Qualitative interviews | Inform development and implementation of an electronic pain monitoring system | Mixture of settings in West Yorkshire (England), sampled via an online pain management survey | Healthcare professionals (palliative care doctor, clinical nurse specialist, general practitioner, community/district nurse) | 15 | Specifies requirements/challenges to overcome in electronic pain monitoring, including the variability in the use of electronic health records between settings and professions, and the ambiguity over who is most responsible for coordinating care (palliative care team or the GP). | Narrow focus on 'PainCheck' tool but refers to broader issues of information sharing systems and ambiguous responsibilities between GPs and specialist palliative care teams. |
| Thelen [132] | 2023 | USA | Qualitative interviews | Understand the interactions between specialist and generalist palliative care teams | Inpatient specialist PC and outpatient generalist PC | 10 specialists and 11 generalist palliative care providers. | 21 | Theory of interdependence explaining how teams vary in their self-perception, communication, and collaboration; and how 'acting as one team' benefits patient outcomes in contrast to 'acting independently'. | All participants from mid-West US and only one oncologist in the sample. Theory appears to be potentially transferable to other settings - similarities w van Gurp paper. |
| Thomas [133] | 2019 | USA | Analysis of patient-clinician consultations | Examine patient-clinician discussions within a randomized trial, comparing content between oncologists and palliative care clinicians | Outpatient clinic, Dana-Farber Cancer Institute | HCPs (inc. 7 oncologists and 10 PC clinicians) and patients (21) | 38 | The two professions play distinct but complementary roles within a co-management model, which benefits patients' understanding of their illness, involvement in treatment decisions, and reinforced coping skills. | Within a trial context – although the co-management model helped to build relationships, which might in some situations facilitate an influence towards integration during usual practice. |
| Toguri [134] | 2020 | Canada | Qualitative interviews | Explore understanding and experience of ACP from patient, caregiver, and provider perspectives | Recruited via hospitals/cancer centres in Nova Scotia. | HCPs (medical and radiation oncologists = 10), patients (4) and caregivers (4) | 18 | Findings on ACP importance and requirements for effective ACP discussions, including informational and training needs. | Small sample and issues with selection bias towards those more willing to talk about ACP ("would you like to talk about health care planning needs and preferences?" used as title on poster). |
| Traeger [135] | 2020 | USA | Analysis of recorded conversations | Explore discussions about treatment discontinuation | Outpatient hospital visits as part of an RCT | Palliative care clinicians, patients and families. | 18 visits, 9 patients | Characterises treatment and hospice decision-making as a process that takes place within a longitudinal clinician-patient-family relationship. | Trial context - allowing resource for palliative care teams to follow patients from soon after diagnosis with incurable cancer. Subsample of larger study. |
| Ullgren [136] | 2022 | Sweden | Qualitative focus groups based on patient cases | To explore how HCPs from acute cancer care and specialist palliative care perceive clinical decision-making when caring for patients receiving treatment and parallel palliative care | Acute hospitals providing cancer treatment and specialist palliative care home teams | Healthcare professionals (14 nurses and 8 physicians) | 22 | The lack of systematic communication between the two teams brings about uncertainty in clinical decision-making, due to unclear responsibilities between the two teams, poor planning or agreement on goals of care, which can increase likelihood of unnecessary transitions of care. | Small focus groups in the region of Stockholm. Some specific aspects (e.g., strict referral criteria for SPC so the person loses their spot if admitted to hospital) might not be relevant. |
| Ullrich [137] | 2022 | Germany | Mixed methods: uptake, PROMs and content analysis | Investigate two different strategies to integrate specialist palliative care into routine oncology practice | Outpatient cancer care department | Patients plus content analysis of consultation with PC | 70 | Integration strategies were well accepted but patients seem not to benefit regarding access from an informational brochure, in that very few sought the PC input after receiving the brochure. | Eligibility criteria could be interpreted as excluding many of those who might perceive their own imminent/current need for extra support. |
| van Dusseldorp [138] | 2019 | Netherlands | Qualitative interviews | Explore what patients associate with their experience of a nurse practitioner in oncology or palliative care | Outpatients recruited from urban hospitals | Patients (mixture of cancer types, ages 45-74) | 17 | Nurse practitioners can play valuable role in providing expert integrated care (leading the battle / putting puzzle pieces in place) that helped patients feel in safe hands. | Participants were recruited by the nurse practitioners, which could have led to selection bias in that those with positive experiences more likely to take part. |
| van Gurp [139] | 2016 | Netherlands | Qualitative interviews and observations | Describe how teleconsultation supports integration of specialist palliative care and primary care | Home-based palliative care | Patients (18), caregivers (17), specialist palliative care team clinicians (12), primary care physicians (15) | 62 | Teleconsultation could support integration by helping professionals define responsibilities for care. Backstage work is still required to build trust and prevent confusion. | Could only include patients living with a caregiver, who might have been more open to this type of care. The researcher occasionally helped with using the technology. |
| van Gurp [140] | 2015 | Netherlands | Qualitative interviews and observations | Explain the impact of teleconsultation on the relationships between home-based patients and hospital-based specialist palliative care teams | Home-based palliative care | Patients (18), caregivers (17), specialist palliative care team clinicians (12), primary care physicians (15) | 62 | Proposes routes by which regular teleconsultation with a consistent professional could build insight into the home situation and trusting relationships - described as transcending the institutional walls of the hospital. | Could only include patients living with a caregiver, who might have been more open to this type of care. The researcher occasionally helped with using the technology, which has implications for feasibility. |
| van Gurp [141] | 2022 | Germany, UK, Belgium, Netherlands, Holland | Qualitative group interviews | Explore the necessary roles and actions to integrate palliative care in Europe | 23 established collaborative PC initiatives | Mixture of roles including medical oncologists, palliative medicine consultants, other physicians, clinical nurse specialists, district nurses, transfer nurses, hospice nurses, GPs, social workers, physiotherapists, pharmacist, pastoral workers, coordinators, administrators. | 136 | Provides detail on the process of building trust and normative integration of palliative care. | Participants had experiences integration and were positive towards it - not necessarily representative of the experiences of those who may feel negatively towards integration. Broad sample and multiple locations. |
| Vater [142] | 2018 | USA | Content analysis | Describe the palliative care content on NCI-designated cancer centre websites. | 62 cancer centre websites | N/A | N/A | Cancer centre webpages rarely mention palliative care services or the benefits of palliative care (or supportive care), including applicability alongside treatment. Acknowledges external motivators towards treatment focus on webpages. Recommends improving information provision to enable integration. | Websites accessed in 2016. |
| Walshe [143] | 2023 | UK | Mixed methods including qualitative interviews | Evaluate an enhanced 7-day specialist palliative care service | Two UK localities, including acute hospital and community-based services | Patients (19), caregivers (23), staff - SPC nurses (24), SPC doctors (5), service manager (2), support worker (1), administrator (1) | 75 (qual) | Perspectives of stakeholders involved in a relevant SPC service indicates improved care standards, reassurance for patients and families, better working environment, and improved staff retention. Extended hours and staff roles conferred relational benefits. Reduced hospital use for cancer patients during the study period (les impactful on resource use for non-cancer patients). | Possible underestimated effect, due to an indistinct 'before' and 'after' during the gradual extension an existing service. Purposive sampling for whether or not a person lived alone, age and gender. Both sites were in economically deprived areas. |
| Walton [144] | 2010 | New Zealand | Qualitative interviews | Identify needs for supportive care in gynaecologic cancer throughout the illness | Recruited from teaching hospital in Auckland | Patients | 28 | Findings suggest lack of continuity and coordination of care results in need for help with navigating the system. Information was important but paper also highlights need for sense of control and experiences to be validated. Additional psychological needs of this type of cancer. | Only 9 of the sample had advanced recurrent disease. |
| Weinstein [145] | 2022 | USA | Retrospective analysis of EOL resource use and costs | Evaluate impact of supportive care programme for advanced cancer patients, on quality of care, quality of life, and costs. | University Hospital and network - LINCC program (Learning Individual Needs and Coordinating Care) involved routine psychosocial screening, early access to SPC, nurse care coordination, implementation of new electronic medical record. | Patients (quant) | 1340 | Healthcare costs in the last 30 days and admissions were significantly lower in the intervention group. Average cost 'per member per month' was higher in intervention group. Intervention appeared effective in improving EOL resource use and likely EOL experience. Notes that implementing the program requires deliberate alignment with strategy plans, leadership support, investment of resources, and commitment to learning. Development of new electronic medical record took place in response to needs observed during implementation. | Intervention group had higher proportion at stage 4, more likely to be Medicaid only, compared to concurrent control. Very small sample for FACT-G scores (n=26) |
| Wind [146] | 2018 | Netherlands | Qualitative interviews | Ascertain the perspectives of GPs and oncologists about who should provide different aspects of care for patients receiving palliative chemotherapy, using scenarios to trigger discussion | Purposefully sampled GPs and oncologists in and around Amsterdam | Healthcare professionals - 12 GPs and 10 oncologists | 22 | Shows differences in reasoning and communication deficits between the professions involved. Contrasting opinions in which both sides said at times they were the most appropriate providers of palliative care. | Scenarios provided may not have included all relevant situations (e.g., urgent care crisis). |
| Wright [147] | 2023 | Canada | Qualitative focus groups and written reflections | Understand nurses perceptions of the appropriateness of palliative care in different cases in relation to their role in 'brokering' palliative care in oncology | Local community hospital with oncology and palliative care in the same clinic/ward | Healthcare professionals (nurses) | 18 | Opinions on the appropriateness of palliative care varied in less straightforward cases. Nurses described hybrid professional identity in which "cancer care" did not distinguish between oncology and palliative aspects, however institutional policies constrained their autonomy to enact this role. | Insights into how these particular nurses understood palliative care and constructed their professional identity - generalisability unclear but potentially promising to other settings. |
| Yang [148] | 2021 | Singapore | Cluster randomised trial | Compare a consult model to a co-rounding model for cancer inpatients | Singapore General Hospital (largest public provider of cancer care in Singapore) | Hospitalised cancer patients | 3167 | Reports that a co-rounding reduces hospital length of stay, especially for patients in need of specialist palliative care. Co-rounding may also be facilitating generalist palliative care and improving relationships between professionals. | Pragmatic trial using cluster design. Did not look at impact on different cancer types (but did look at stages). This quant work seems to go alongside other qual work on the co-rounding model. |
| Yang [149] | 2018 | Singapore | Qualitative interviews | Explore views on a pilot of an inpatient co-rounding model compared to traditional consult model. | Singapore General Hospital (largest public provider of cancer care in Singapore) | Healthcare professionals (physicians and nurses) | 11 | Reports potential benefits of co-rounding model, for efficiency of care delivery, quality of holistic care, and education of oncology team. | Possible selection bias in that those with negative experiences of the pilot less likely to participate. |
| Yoong [150] | 2013 | USA | Content analysis of notes from patient-clinician interactions | Identify key elements of early palliative care and explore their timing, comparing content of palliative and oncologic notes | RCT of early PC, at thoracic oncology clinic at Massachusetts General Hospital | Patients (20) and their palliative care or oncology clinicians | 20 | Distinct features of palliative care and oncology at critical time points, argues that palliative care involvement concurrently to treatment could save oncologists time, demonstrates that the initial visits do not unnecessarily confront mortality. | RCT context, limited on the implementation of the intervention as focuses on content of interactions only. |
| Zemplenyi [151] | 2021 | Hungary | Retrospective analysis | Measure association of palliative care consult with healthcare costs | Clinical Centre at the University Pecs (providing hospital palliative care) | Patients (quant) | 197 | Intervention associated with reduced likelihood of dying in hospital and cost savings for the healthcare system. | Unclear how the intervention group ended up getting the intervention, whether they differed from those receiving usual care. |
| Zimmermann [152] | 2016 | Canada | Qualitative interviews | Assess attitudes and perceptions about palliative care as part of an RCT of EPC | Recruited from cancer centre in Toronto | Patients and caregivers | 71 | Findings that the stigma of palliative care might persist even after positive PC experience. Recommends education of public, patients, and healthcare providers. Discusses the possible implications of changing PC terminology. | Embedded qual study within an RCT. Some aspects may be trial specific but appears in alignment with similar work. |
| Zimmermann [153] | 2023 | Canada | Mixed methods including qualitative interviews | RCT of symptom screening with targeted early palliative care (STEP) | Outpatient oncology and palliative care | Cancer patients | 69 (16 qual) | Evaluates one approach to identifying patient need to facilitate communication with oncology nurses, with high scores triggering referral for specialist palliative care. | RCT was disrupted by the Covid-19 pandemic and associated shift to virtual consultations. Further research needed to test virtual STEP model. |

1. Abunasser, M., et al., *Aggressiveness of Cancer Care at End of Life in Patients with Metastatic Breast Cancer in Jordan.* J Multidiscip Healthc, 2023. **16**: p. 2873-2881.

2. Adelson, K., et al., *Standardized Criteria for Palliative Care Consultation on a Solid Tumor Oncology Service Reduces Downstream Health Care Use.* J Oncol Pract, 2017. **13**(5): p. e431-e440.

3. Ahmed, S., et al., *Patient and caregiver experiences with advanced cancer care: a qualitative study informing the development of an early palliative care pathway.* BMJ Support Palliat Care, 2020.

4. Alizadeh, Z., et al., *Challenges of Integrated Home-Based Palliative Care Services for Cancer Patients during the COVID-19 Pandemic: A Qualitative Content Analysis.* Home Health Care Management & Practice, 2022. **35**(3): p. 180-189.

5. Back, A.L., et al., *Clinician roles in early integrated palliative care for patients with advanced cancer: a qualitative study.* J Palliat Med, 2014. **17**(11): p. 1244-8.

6. Bakitas, M.A., et al., *Early Versus Delayed Initiation of Concurrent Palliative Oncology Care: Patient Outcomes in the ENABLE III Randomized Controlled Trial.* J Clin Oncol, 2015. **33**(13): p. 1438-45.

7. Bakitas, M., K.D. Lyons, M.T. Hegel, and T. Ahles, *Oncologists' perspectives on concurrent palliative care in a National Cancer Institute-designated comprehensive cancer center.* Palliat Support Care, 2013. **11**(5): p. 415-23.

8. Bakken, J., et al., *Organizational structures influencing timely recognition and acknowledgment of end-of-life in hospitals - A qualitative study of nurses' and doctors' experiences.* Eur J Oncol Nurs, 2023. **67**: p. 102420.

9. Beernaert, K., et al., *Is There a Need for Early Palliative Care in Patients With Life-Limiting Illnesses? Interview Study With Patients About Experienced Care Needs From Diagnosis Onward.* Am J Hosp Palliat Care, 2016. **33**(5): p. 489-97.

10. Beernaert, K., et al., *Family physicians' role in palliative care throughout the care continuum: stakeholder perspectives.* Fam Pract, 2015. **32**(6): p. 694-700.

11. Behl, D. and A. Jatoi, *What do oncologists say about chemotherapy at the very end of life? Results from a semiqualitative survey.* J Palliat Med, 2010. **13**(7): p. 831-5.

12. Beiranvand, S., et al., *Hospice care delivery system requirements.* Int J Palliat Nurs, 2022. **28**(12): p. 562-574.

13. Bennardi, M., et al., *A qualitative analysis of educational, professional and socio-cultural issues affecting interprofessional collaboration in oncology palliative care.* Patient Educ Couns, 2022. **105**(9): p. 2976-2983.

14. Bennardi, M., et al., *A qualitative exploration of interactional and organizational determinants of collaboration in cancer palliative care settings: Family members', health care professionals' and key informants' perspectives.* PLoS One, 2021. **16**(10): p. e0256965.

15. Bergqvist, J. and G. Ljunggren, *The Impact of Integrated Home Palliative Care Services on Resource Use and Place of Death.* J Palliat Med, 2020. **23**(1): p. 67-73.

16. Bischoff, K.E., et al., *Embedded palliative care for patients with metastatic colorectal cancer: a mixed-methods pilot study.* Support Care Cancer, 2020. **28**(12): p. 5995-6010.

17. Blackhall, L.J., et al., *CARE Track for Advanced Cancer: Impact and Timing of an Outpatient Palliative Care Clinic.* J Palliat Med, 2016. **19**(1): p. 57-63.

18. Boyd, K., et al., *Advance care planning for cancer patients in primary care: a feasibility study.* Br J Gen Pract, 2010. **60**(581): p. e449-58.

19. Brazil, K., D. Bainbridge, and C. Rodriguez, *The stress process in palliative cancer care: a qualitative study on informal caregiving and its implication for the delivery of care.* Am J Hosp Palliat Care, 2010. **27**(2): p. 111-6.

20. Brenne, A.T., et al., *Implementing a Standardized Care Pathway Integrating Oncology, Palliative Care and Community Care in a Rural Region of Mid-Norway.* Oncol Ther, 2021. **9**(2): p. 671-693.

21. Bull, J.H., et al., *Demonstration of a sustainable community-based model of care across the palliative care continuum.* J Pain Symptom Manage, 2012. **44**(6): p. 797-809.

22. Capodanno, I., et al., *Caregivers of Patients with Hematological Malignancies within Home Care: A Phenomenological Study.* Int J Environ Res Public Health, 2020. **17**(11).

23. Cerni, J., J. Rhee, and H. Hosseinzadeh, *Challenges and strategies to improve the provision of end-of-life cancer care in rural and regional communities: Perspectives from Australian rural health professionals.* Aust J Rural Health, 2023.

24. Chang, S., et al., *A Palliative Radiation Oncology Consult Service Reduces Total Costs During Hospitalization.* J Pain Symptom Manage, 2018. **55**(6): p. 1452-1458.

25. Collins, A., et al., *Communication about early palliative care: A qualitative study of oncology providers' perspectives of navigating the artful introduction to the palliative care team.* Front Oncol, 2022. **12**: p. 1003357.

26. Collins, A., et al., *The feasibility of triggers for the integration of Standardised, Early Palliative (STEP) Care in advanced cancer: A phase II trial.* Front Oncol, 2022. **12**: p. 991843.

27. Collins, A., S.A. McLachlan, and J. Philip, *Initial perceptions of palliative care: An exploratory qualitative study of patients with advanced cancer and their family caregivers.* Palliat Med, 2017. **31**(9): p. 825-832.

28. Collins, A., et al., *The challenges and suffering of caring for people with primary malignant glioma: qualitative perspectives on improving current supportive and palliative care practices.* BMJ Support Palliat Care, 2013. **4**(1): p. 68-76.

29. Costantini, M., et al., *Is early integration of palliative care feasible and acceptable for advanced respiratory and gastrointestinal cancer patients? A phase 2 mixed-methods study.* Palliat Med, 2018. **32**(1): p. 46-58.

30. Cushen-Brewster, N., et al., *Evaluating a specialist palliative care service in a community setting.* British Journal of Healthcare Management, 2023. **29**(10): p. 1-13.

31. Dahlhaus, A., et al., *Involvement of general practitioners in palliative cancer care: a qualitative study.* Support Care Cancer, 2013. **21**(12): p. 3293-300.

32. Davies, N., et al., *Quality palliative care for cancer and dementia in five European countries: some common challenges.* Aging Ment Health, 2014. **18**(4): p. 400-10.

33. Delisle, M.E., et al., *Timing of Palliative Care in Colorectal Cancer Patients: Does It Matter?* J Surg Res, 2019. **241**: p. 285-293.

34. Desai, A.V., et al., *Palliative Medicine in Myelodysplastic Syndromes: Patients and Caregivers - A Qualitative Study.* BMJ Support Palliat Care, 2021.

35. Dhollander, N., et al., *Is early integration of palliative home care in oncology treatment feasible and acceptable for advanced cancer patients and their health care providers? A phase 2 mixed-methods study.* BMC Palliat Care, 2020. **19**(1): p. 174.

36. Dhollander, N., et al., *Barriers to the early integration of palliative home care into the disease trajectory of advanced cancer patients: A focus group study with palliative home care teams.* Eur J Cancer Care (Engl), 2019. **28**(4): p. e13024.

37. Dhollander, N., et al., *Differences between early and late involvement of palliative home care in oncology care: A focus group study with palliative home care teams.* Palliat Med, 2018. **32**(7): p. 1275-1282.

38. Dhollander, N., et al., *Phase 0-1 early palliative home care cancer treatment intervention study.* BMJ Support Palliat Care, 2022. **12**(e1): p. e103-e111.

39. Economos, G., et al., *Palliative care from the perspective of cancer physicians: a qualitative semistructured interviews study.* BMJ Support Palliat Care, 2023. **13**(1): p. 95-101.

40. Ellen, M.E., S. Perlman, and R. Shach, *Too Much Cancer Care?: Nurses' Perspectives on the Unnecessary Use of Oncology Services.* Cancer Nurs, 2021. **44**(4): p. E236-E243.

41. Emiloju, O.E., D.A.M. Djibo, and J.G. Ford, *Association Between the Timing of Goals-of-Care Discussion and Hospitalization Outcomes in Patients With Metastatic Cancer.* Am J Hosp Palliat Care, 2020. **37**(6): p. 433-438.

42. Ervik, B., B. Brondbo, and M.L. Johansen, *Adapting and Going the Extra Mile: A Qualitative Study of Palliative Care in Rural Northern Norway From the Perspective of Healthcare Providers.* Cancer Nurs, 2021. **44**(4): p. E229-E235.

43. Ervik, B., T. Donnem, and M.L. Johansen, *Dying at "home" - a qualitative study of end-of-life care in rural Northern Norway from the perspective of health care professionals.* BMC Health Serv Res, 2023. **23**(1): p. 1359.

44. Evans, J.M., et al., *Integrating early palliative care into routine practice for patients with cancer: A mixed methods evaluation of the INTEGRATE Project.* Psychooncology, 2019. **28**(6): p. 1261-1268.

45. Ferrell, B.R., R. Virani, E. Han, and P. Mazanec, *Integration of Palliative Care in the Role of the Oncology Advanced Practice Nurse.* J Adv Pract Oncol, 2021. **12**(2): p. 165-172.

46. Fox, J.A., J. Rosenberg, S. Ekberg, and D. Langbecker, *Palliative care in the context of immune and targeted therapies: A qualitative study of bereaved carers' experiences in metastatic melanoma.* Palliat Med, 2020. **34**(10): p. 1351-1360.

47. Frissen, A.R., et al., *Experiences of healthcare professionals with support for mesothelioma patients and their relatives: Identified gaps and improvements for care.* Eur J Cancer Care (Engl), 2021. **30**(6): p. e13509.

48. Garcia, R., et al., *The Team-Based Serious Illness Care Program, A Qualitative Evaluation of Implementation and Teaming.* J Pain Symptom Manage, 2023. **65**(6): p. 521-531.

49. Gardiner, C., M. Harrison, S. Hargreaves, and B. Taylor, *Clinical nurse specialist role in providing generalist and specialist palliative care: A qualitative study of mesothelioma clinical nurse specialists.* J Adv Nurs, 2022. **78**(9): p. 2973-2982.

50. Gerlach, C., et al., *Space for intuition - the 'Surprise'-Question in haemato-oncology: Qualitative analysis of experiences and perceptions of haemato-oncologists.* Palliat Med, 2019. **33**(5): p. 531-540.

51. Gonzalez, R., et al., *Impact of early vs late palliative care referrals on healthcare utilization in patients with pancreatic cancer.* J Cancer Res Clin Oncol, 2023. **149**(16): p. 14997-15002.

52. Gott, M., et al., *'That's part of everybody's job': the perspectives of health care staff in England and New Zealand on the meaning and remit of palliative care.* Palliat Med, 2012. **26**(3): p. 232-41.

53. Greer, J.A., et al., *Cost Analysis of a Randomized Trial of Early Palliative Care in Patients with Metastatic Nonsmall-Cell Lung Cancer.* J Palliat Med, 2016. **19**(8): p. 842-8.

54. Gross, J.P., et al., *Radiation Oncologists' Role in End-of-Life Care: A Perspective From Medical Oncologists.* Pract Radiat Oncol, 2019. **9**(5): p. 362-370.

55. Hahne, J., et al., *Chinese physicians' perceptions of palliative care integration for advanced cancer patients: a qualitative analysis at a tertiary hospital in Changsha, China.* BMC Med Ethics, 2022. **23**(1): p. 17.

56. Halling, C.M.B., et al., *Cost-effectiveness analysis of systematic fast-track transition from oncological treatment to specialised palliative care at home for patients and their caregivers: the DOMUS trial.* BMC Palliat Care, 2020. **19**(1): p. 142.

57. Hannon, B., et al., *Early Palliative Care and Its Role in Oncology: A Qualitative Study.* Oncologist, 2016. **21**(11): p. 1387-1395.

58. Hasegawa, T., et al., *Integrating home palliative care in oncology: a qualitative study to identify barriers and facilitators.* Support Care Cancer, 2022. **30**(6): p. 5211-5219.

59. Hayden, L. and S. Dunne, *"Dying With Dignity": A Qualitative Study With Caregivers on the Care of Individuals With Terminal Cancer.* Omega (Westport), 2022. **84**(4): p. 1122-1145.

60. Henson, L.A., et al., *'I'll be in a safe place': a qualitative study of the decisions taken by people with advanced cancer to seek emergency department care.* BMJ Open, 2016. **6**(11): p. e012134.

61. Hoek, D., et al., *Role of GPs in shared decision making with patients about palliative cancer treatment: a qualitative study in the Netherlands.* Br J Gen Pract, 2022. **72**(717): p. e276-e284.

62. Hojjat-Assari, S., M. Rassouli, V. Kaveh, and H. Heydari, *Explaining health care providers' perceptions about the integration of palliative care with primary health care; a qualitative study.* BMC Prim Care, 2022. **23**(1): p. 226.

63. Horlait, M., S. Van Belle, and M. Leys, *The need for adequate communication training programs for palliative care in multidisciplinary teams in oncology settings.* International Journal of Integrated Care, 2016. **16**(6): p. 1-2.

64. Hoverman, J.R., et al., *Hospice or Hospital: The Costs of Dying of Cancer in the Oncology Care Model.* Palliat Med Rep, 2020. **1**(1): p. 92-96.

65. Howell, D.M., et al., *Predictors of home care expenditures and death at home for cancer patients in an integrated comprehensive palliative home care pilot program.* Healthcare policy = Politiques de sante, 2011. **6**(3): p. e73-92.

66. Huo, J., et al., *Timing, Costs, and Survival Outcome of Specialty Palliative Care in Medicare Beneficiaries With Metastatic Non-Small-Cell Lung Cancer.* JCO Oncol Pract, 2020. **16**(12): p. e1532-e1542.

67. Johansen, M.L. and B. Ervik, *Teamwork in primary palliative care: general practitioners' and specialised oncology nurses' complementary competencies.* BMC Health Serv Res, 2018. **18**(1): p. 159.

68. Johansen, M.L. and B. Ervik, *Talking together in rural palliative care: a qualitative study of interprofessional collaboration in Norway.* BMC Health Serv Res, 2022. **22**(1): p. 314.

69. Johnston, B., et al., *Integrating palliative care in lung cancer: an early feasibility study.* Int J Palliat Nurs, 2013. **19**(9): p. 433-7.

70. Kaye, D.R., et al., *System-Level Health-Care Integration and the Costs of Cancer Care Across the Disease Continuum.* J Oncol Pract, 2018. **14**(3): p. e149-e157.

71. Keim-Malpass, J., E.M. Mitchell, L. Blackhall, and P.B. DeGuzman, *Evaluating Stakeholder-Identified Barriers in Accessing Palliative Care at an NCI-Designated Cancer Center with a Rural Catchment Area.* J Palliat Med, 2015. **18**(7): p. 634-7.

72. Kitta, A., et al., *The silent transition from curative to palliative treatment: a qualitative study about cancer patients' perceptions of end-of-life discussions with oncologists.* Support Care Cancer, 2021. **29**(5): p. 2405-2413.

73. Kleiner, N., S.C. Zambrano, S. Eychmuller, and S. Zwahlen, *Early palliative care integration trial: consultation content and interaction dynamics.* BMJ Support Palliat Care, 2021.

74. Krause, R., L. Gwyther, and J. Olivier, *Evaluating a vertical nurse-led service in the integration of palliative care in a tertiary academic hospital.* Palliat Care Soc Pract, 2024. **18**: p. 26323524231224806.

75. Krause, R., L. Gwyther, and J. Olivier, *The influence of context on the implementation of integrated palliative care in an academic teaching hospital in South Africa.* Palliat Care Soc Pract, 2024. **18**: p. 26323524231219510.

76. Kremenova, Z., et al., *Does a Hospital Palliative Care Team Have the Potential to Reduce the Cost of a Terminal Hospitalization? A Retrospective Case-Control Study in a Czech Tertiary University Hospital.* J Palliat Med, 2022. **25**(7): p. 1088-1094.

77. Kruser, T.J., et al., *Medical oncologist perspectives on palliative care reveal physician-centered barriers to early integration.* Ann Palliat Med, 2020. **9**(5): p. 2800-2808.

78. Kubendran, S., et al., *Trends in inpatient palliative care use for primary brain malignancies.* Support Care Cancer, 2021. **29**(11): p. 6625-6632.

79. Le, B.H., et al., *Acceptability of early integration of palliative care in patients with incurable lung cancer.* J Palliat Med, 2014. **17**(5): p. 553-8.

80. LeBlanc, T.W., et al., *Perceptions of palliative care among hematologic malignancy specialists: a mixed-methods study.* J Oncol Pract, 2015. **11**(2): p. e230-8.

81. Lee, J.T., et al., *Dying of mesothelioma: A qualitative exploration of caregiver experiences.* Eur J Cancer Care (Engl), 2022. **31**(5): p. e13627.

82. Liu, Y., et al., *Application of interdisciplinary collaborative hospice care for terminal geriatric cancer patients: a prospective randomized controlled study.* Support Care Cancer, 2022. **30**(4): p. 3553-3561.

83. Lundeby, T., et al., *Challenges and Learning Needs for Providers of Advanced Cancer Care: Focus Group Interviews with Physicians and Nurses.* Palliat Med Rep, 2020. **1**(1): p. 208-215.

84. Lundeby, T., et al., *A complex communication skills training program for physicians providing advanced cancer care - content development and barriers and solutions for implementation.* J Commun Healthc, 2023. **16**(1): p. 46-57.

85. Lundereng, E.D., A. Dihle, and S.A. Steindal, *Nurses' experiences and perspectives on collaborative discharge planning when patients receiving palliative care for cancer are discharged home from hospitals.* J Clin Nurs, 2020. **29**(17-18): p. 3382-3391.

86. Maessen, M., et al., *An economic evaluation of an early palliative care intervention among patients with advanced cancer.* Swiss Med Wkly, 2024. **154**: p. 3591.

87. Mashiro, E., H. Arao, M. Aoki, and Y. Matsumoto, *What are the barriers to medical collaboration in community-based integrated care supporting cancer patients? A qualitative analysis of healthcare and long-term care providers' perceptions.* Jpn J Clin Oncol, 2023. **53**(12): p. 1162-1169.

88. Mayland, C.R., et al., *The palliative care needs and experiences of people with advanced head and neck cancer: A scoping review.* Palliat Med, 2021. **35**(1): p. 27-44.

89. McCaffrey, N., et al., *Is home-based palliative care cost-effective? An economic evaluation of the Palliative Care Extended Packages at Home (PEACH) pilot.* BMJ Support Palliat Care, 2013. **3**(4): p. 431-5.

90. McCaughan, D., et al., *Haematology nurses' perspectives of their patients' places of care and death: A UK qualitative interview study.* Eur J Oncol Nurs, 2019. **39**: p. 70-80.

91. McPherson, S., et al., *Haematological nurses' experiences about palliative care trajectories of patients with life-threatening haematological malignancies: A qualitative study.* Nurs Open, 2023. **10**(5): p. 3094-3103.

92. Mensah, A.B.B., et al., *Expectations and barriers to the utilization of specialist palliative care services among persons living with cancer in Ghana: an exploratory qualitative study.* Palliat Care Soc Pract, 2023. **17**: p. 26323524231193042.

93. Mollica, M.A., et al., *Perspectives on Palliative Care in Cancer Clinical Trials: Diverse Meanings from Multidisciplinary Cancer Care Providers.* J Palliat Med, 2018. **21**(5): p. 616-621.

94. Monnery, D., et al., *Delivery Models and Health Economics of Supportive Care Services in England: A Multicentre Analysis.* Clin Oncol (R Coll Radiol), 2023. **35**(6): p. e395-e403.

95. Montiel, C., et al., *Barriers and facilitators of supportive care access and use among men with cancer: a qualitative study.* J Cancer Surviv, 2023.

96. Morikawa, M., Y. Shirai, R. Ochiai, and K. Miyagawa, *Barriers to the Collaboration Between Hematologists and Palliative Care Teams on Relapse or Refractory Leukemia and Malignant Lymphoma Patients' Care: A Qualitative Study.* American Journal of Hospice & Palliative Medicine, 2016. **33**(10): p. 977-984.

97. Ndiok, A. and B. Ncama, *Barriers and benefits of model development for integration of palliative care for cancer patients in a developing country: A qualitative study.* Int J Nurs Pract, 2021. **27**(3): p. e12884.

98. Ndiok, A. and B. Ncama, *A qualitative study of home visiting as a palliative care strategy to follow-up cancer patients by nurses in clinical setting in a developing country.* Scand J Caring Sci, 2019. **33**(1): p. 185-196.

99. Okyere, J. and K. Kissah-Korsah, *Barriers to the integration of palliative care in Ghana: evidence from a tertiary health facility.* Palliat Care Soc Pract, 2023. **17**: p. 26323524231179980.

100. Olafsdottir, K.L., et al., *Integrating nurse-facilitated advance care planning for patients newly diagnosed with advanced lung cancer.* Int J Palliat Nurs, 2018. **24**(4): p. 170-177.

101. Payne, S., et al., *Enhancing integrated palliative care: what models are appropriate? A cross-case analysis.* BMC Palliat Care, 2017. **16**(1): p. 64.

102. Perry, L.M., et al., *Increasing Readiness for Early Integrated Palliative Oncology Care: Development and Initial Evaluation of the EMPOWER 2 Intervention.* J Pain Symptom Manage, 2021. **62**(5): p. 987-996.

103. Porto, A.R., et al., *The essence of interdisciplinary practice in palliative care delivery to cancer patients.* Investigacion & Educacion en Enfermeria, 2012. **30**(2): p. 231-239.

104. Preisler, M., et al., *Early palliative care for those who care: A qualitative exploration of cancer caregivers' information needs during hospital stays.* Eur J Cancer Care (Engl), 2019. **28**(2): p. e12990.

105. Preisler, M., et al., *Early integration of palliative cancer care: patients' and caregivers' challenges, treatment preferences, and knowledge of illness and treatment throughout the cancer trajectory.* Support Care Cancer, 2018. **26**(3): p. 921-931.

106. Prod'homme, C., et al., *Barriers to end-of-life discussions among hematologists: A qualitative study.* Palliat Med, 2018. **32**(5): p. 1021-1029.

107. Rao, S.R., N. Salins, C.R. Goh, and S. Bhatnagar, *"Building palliative care capacity in cancer treatment centres: a participatory action research".* BMC Palliat Care, 2022. **21**(1): p. 101.

108. Raunkiaer, M., M.S. Buch, C. Holm-Petersen, and H. Timm, *Professionals' experiences with palliative care and collaboration in relation to a randomised clinical trial: a qualitative interview study.* Scand J Caring Sci, 2020. **34**(2): p. 305-313.

109. Raunkiaer, M., et al., *When and how to stop palliative antineoplastic treatment and to organise palliative care for patients with incurable cancer.* Int J Palliat Nurs, 2023. **29**(10): p. 499-506.

110. Ribi, K., N. Kalbermatten, M. Eicher, and F. Strasser, *Towards a novel approach guiding the decision-making process for anticancer treatment in patients with advanced cancer: framework for systemic anticancer treatment with palliative intent.* ESMO Open, 2022. **7**(3): p. 100496.

111. Roberson, M.L., et al., *Re-imagining metastatic breast cancer care delivery: a patient-partnered qualitative study.* Support Care Cancer, 2023. **31**(12): p. 735.

112. Rohrmoser, A., et al., *Early integration of palliative/supportive cancer care-healthcare professionals' perspectives on the support needs of cancer patients and their caregivers across the cancer treatment trajectory.* Support Care Cancer, 2017. **25**(5): p. 1621-1627.

113. Rossi, R., et al., *The challenge of sustainability in healthcare systems: cost of radiotherapy in the last month of life in an Italian cancer center.* Support Care Cancer, 2021. **29**(5): p. 2735-2742.

114. Sadang, K.G., et al., *Clinician Perceptions of Barriers and Facilitators for Delivering Early Integrated Palliative Care via Telehealth.* Cancers (Basel), 2023. **15**(22).

115. Satija, A., et al., *Quality Improvement in Itself Changes Your Thinking: Lessons From Disseminating Quality Improvement Methods Through a Multisite International Collaborative Palliative Care Project in India.* JCO Glob Oncol, 2022. **8**: p. e2200147.

116. Schenker, Y., et al., *A Pilot Trial of Early Specialty Palliative Care for Patients with Advanced Pancreatic Cancer: Challenges Encountered and Lessons Learned.* J Palliat Med, 2018. **21**(1): p. 28-36.

117. Schenker, Y., et al., *Oncologist factors that influence referrals to subspecialty palliative care clinics.* J Oncol Pract, 2014. **10**(2): p. e37-44.

118. Schifferdecker, K.E., et al., *Structure and integration of specialty palliative care in three NCI-designated cancer centers: a mixed methods case study.* BMC Palliat Care, 2023. **22**(1): p. 59.

119. Senior, L. and G. Hubbard, *Integrated malignant and non-malignant palliative care in Scotland.* Br J Community Nurs, 2010. **15**(6): p. 284-91.

120. Seow, H., et al., *Effect of Early Palliative Care on End-of-Life Health Care Costs: A Population-Based, Propensity Score-Matched Cohort Study.* JCO Oncol Pract, 2022. **18**(1): p. e183-e192.

121. Siegle, A., et al., *Communication with patients with limited prognosis-an integrative mixed-methods evaluation study.* Support Care Cancer, 2022. **31**(1): p. 77.

122. Siler, S., I. Mamier, and B. Winslow, *The Perceived Facilitators and Challenges of Translating a Lung Cancer Palliative Care Intervention Into Community-Based Settings.* J Hosp Palliat Nurs, 2018. **20**(4): p. 407-415.

123. Skorpen Tarberg, A., et al., *Nurses' experiences of compassionate care in the palliative pathway.* J Clin Nurs, 2020. **29**(23-24): p. 4818-4826.

124. Sommer, J., C. Chung, D.M. Haller, and S. Pautex, *Shifting palliative care paradigm in primary care from better death to better end-of-life: a Swiss pilot study.* BMC Health Serv Res, 2021. **21**(1): p. 629.

125. Spelten, E.R., et al., *Making community palliative and end-of-life care sustainable; investigating the adaptability of rural Australian service provision.* Health Soc Care Community, 2021. **29**(6): p. 1998-2007.

126. Stern, A., R. Valaitis, R. Weir, and A.R. Jadad, *Use of home telehealth in palliative cancer care: a case study.* J Telemed Telecare, 2012. **18**(5): p. 297-300.

127. Stewart, E., et al., *Cancer centre supportive oncology service: health economic evaluation.* BMJ Support Palliat Care, 2022.

128. Sullivan, D.R., et al., *Relationships among clinicians are crucial to successful palliative care integration: a qualitative study in lung cancer.* Future Oncol, 2023. **19**(3): p. 245-257.

129. Tartaglione, E.V., E.K. Vig, and L.F. Reinke, *Bridging the Cultural Divide Between Oncology and Palliative Care Subspecialties: Clinicians' Perceptions on Team Integration.* Am J Hosp Palliat Care, 2018. **35**(7): p. 978-984.

130. Tate, A., *Death and the treatment imperative: Decision-making in late-stage cancer.* Soc Sci Med, 2022. **306**: p. 115129.

131. Taylor, S., et al., *Identifying professionals' needs in integrating electronic pain monitoring in community palliative care services: An interview study.* Palliat Med, 2017. **31**(7): p. 661-670.

132. Thelen, M., S.G. Brearley, and C. Walshe, *A grounded theory of interdependence between specialist and generalist palliative care teams across healthcare settings.* Palliat Med, 2023. **37**(10): p. 1474-1483.

133. Thomas, T.H., et al., *Communication Differences between Oncologists and Palliative Care Clinicians: A Qualitative Analysis of Early, Integrated Palliative Care in Patients with Advanced Cancer.* J Palliat Med, 2019. **22**(1): p. 41-49.

134. Toguri, J.T., L. Grant-Nunn, and R. Urquhart, *Views of advanced cancer patients, families, and oncologists on initiating and engaging in advance care planning: a qualitative study.* BMC Palliat Care, 2020. **19**(1): p. 150.

135. Traeger, L., et al., *Nature of Discussions about Systemic Therapy Discontinuation or Hospice among Patients, Families, and Palliative Care Clinicians during Care for Incurable Cancer: A Qualitative Study.* Journal of Palliative Medicine, 2020. **23**(4): p. 542-547.

136. Ullgren, H., L. Sharp, P. Fransson, and K. Bergkvist, *Exploring Health Care Professionals' Perceptions Regarding Shared Clinical Decision-Making in Both Acute and Palliative Cancer Care.* Int J Environ Res Public Health, 2022. **19**(23).

137. Ullrich, A., et al., *Acceptance and Benefits of Two Different Strategies to Timely Integrate Specialist Palliative Care into Routine Cancer Care: A Randomized Pilot Study.* Oncol Res Treat, 2022. **45**(3): p. 118-129.

138. van Dusseldorp, L., et al., *What does the nurse practitioner mean to you? A patient-oriented qualitative study in oncological/palliative care.* J Clin Nurs, 2019. **28**(3-4): p. 589-602.

139. van Gurp, J., et al., *Teleconsultation for integrated palliative care at home: A qualitative study.* Palliat Med, 2016. **30**(3): p. 257-69.

140. van Gurp, J., et al., *How outpatient palliative care teleconsultation facilitates empathic patient-professional relationships: a qualitative study.* PLoS One, 2015. **10**(4): p. e0124387.

141. van Gurp, J., et al., *Integrating Palliative Care by Virtue of Diplomacy; A Cross-sectional Group Interview Study of the Roles and Attitudes of Palliative Care Professionals to Further Integrate Palliative Care in Europe.* Int J Health Policy Manag, 2022. **11**(6): p. 786-794.

142. Vater, L.B., et al., *Palliative care content on cancer center websites.* Support Care Cancer, 2018. **26**(3): p. 1005-1011.

143. Walshe, C., et al., *'Thank goodness you're here'. Exploring the impact on patients, family carers and staff of enhanced 7-day specialist palliative care services: A mixed methods study.* Palliat Med, 2023. **37**(10): p. 1484-1497.

144. Walton, L.M., J. Reeve, P.M. Brown, and C.M. Farquhar, *Gynaecologic cancer patients' needs and experiences of supportive health services in New Zealand.* Psychooncology, 2010. **19**(2): p. 201-8.

145. Weinstein, E., et al., *Quality and cost outcomes of an integrated supportive care program.* Support Care Cancer, 2022. **30**(1): p. 535-542.

146. Wind, J., et al., *Who should provide care for patients receiving palliative chemotherapy? A qualitative study among Dutch general practitioners and oncologists.* Scand J Prim Health Care, 2018. **36**(4): p. 437-445.

147. Wright, D.K., et al., *"We're Cancer Care Nurses": Perceptions About Providing Palliative Care in a Community Hospital.* J Hosp Palliat Nurs, 2023. **25**(2): p. 82-89.

148. Yang, G.M., et al., *Comparing the effect of a consult model versus an integrated palliative care and medical oncology co-rounding model on health care utilization in an acute hospital - an open-label stepped-wedge cluster-randomized trial.* Palliat Med, 2021. **35**(8): p. 1578-1589.

149. Yang, G.M., S. Yoon, Y.Y. Tan, and K. Liaw, *Experience and Views of Oncology and Palliative Care Professionals on a Corounding Model of Care for Inpatients With Advanced Cancer.* Am J Hosp Palliat Care, 2018. **35**(11): p. 1433-1438.

150. Yoong, J., et al., *Early palliative care in advanced lung cancer: a qualitative study.* JAMA Intern Med, 2013. **173**(4): p. 283-90.

151. Zemplenyi, A.T., et al., *Early palliative care associated with lower costs for adults with advanced cancer: evidence from Hungary.* Eur J Cancer Care (Engl), 2021. **30**(6): p. e13473.

152. Zimmermann, C., et al., *Perceptions of palliative care among patients with advanced cancer and their caregivers.* CMAJ, 2016. **188**(10): p. E217-E227.

153. Zimmermann, C., et al., *Symptom screening with Targeted Early Palliative care (STEP) versus usual care for patients with advanced cancer: a mixed methods study.* Support Care Cancer, 2023. **31**(7): p. 404.
