# Supplementary material for: Integrated palliative care and oncology: a realist synthesis
Source: BMC Med. 2025 May 9;23:272. doi: 10.1186/s12916-025-04083-1 (PMC12065255; doi:10.1186/s12916-025-04083-1)
Supplement: Supplementary file 3 — Additional file 3. Detailed context-mechanism-outcome configurations [file 12916_2025_4083_MOESM3_ESM.docx]

## Additional file 3: Context-Mechanism-Outcome Configurations

### Section 1: Sense of coherence (understanding what needs to be done)

This section contains four CMOCs, shown in Box 1.

Box 1: CMOCs for section 1, sense of coherence (understanding what needs to be done)

CMOC1a: Conceptualising cancer care

When healthcare professionals experience palliative care as an important part of comprehensive cancer care (C), then because they are motivated to do what’s best for patients (M), these professionals will be more likely to accept or involve palliative care (O).

CMOC1b: Leadership support

When organisation, system, or professional leadership demonstrate consistent support for the integration of palliative care (C), staff believe that palliative care is important to their institution/profession (M), which motivates them to prioritise integration of palliative care (O).

CMOC1c: Professional role clarification

When roles and responsibilities for delivering integrated palliative care are clearly defined (C), then professionals can understand what is expected of them and what they can expect from others (M), which facilitates communication and collaboration (O).

CMOC1d: Societal perceptions of palliative care

When media or public health campaigns explain the benefits of integrating palliative care (C), then people with cancer and their families can better understand how they/their family could benefit (M), and so are more likely to accept or ask for palliative care (O).

| Conceptualising cancer care | |
| --- | --- |
| **CMOC1a** | When healthcare professionals experience palliative care as an important part of comprehensive cancer care (C), then because they are motivated to do what’s best for patients (M), these professionals will be more likely to accept or involve palliative care (O). |
| There can be widespread misconceptions that palliative care is end-of-life care only, and therefore not applicable alongside active cancer treatment [1, 2] – in some cases even perceived as a ‘no care, no choice’ route [3]. Misunderstandings of palliative care therefore remain a substantial barrier to integration with oncology due to the proliferation of treatment options available and related prognostic challenges [4-6].  Under the right circumstances, palliative care can be viewed as an essential component of comprehensive treatment or cancer care [7, 8]. Exposure to palliative care during training leads to greater motivation towards integration [9]. Academic curricula could encourage a combined vision where both treatment and care are important in meeting the holistic needs of people with cancer [10-12]. Strategies include mandatory and continued education extended to a wider range of clinical disciplines [13, 14]. | |
| Illustrative quotes | |
| Wright, 2023 [8] | *“Conceptualizing palliative care as a discrete service from acute care maintains and reinforces the conceptual divide between the two, with acute care clinicians at risk of failing to identify and respond to palliative care needs because it is “too soon’.”* |
| Wright, 2023 [8] | *“Nurses described a hybrid professional identity that integrates both oncology and palliative care. To them, this integration constituted the meaning of “cancer care.” … … The idea that cancer care nurses seem to integrate palliative care into their nursing identity holds great promise for cancer patients.”* |
| Hahne, 2022 [7] | *“While doctors generally agreed palliative care could help relieve suffering… most doctors conceptualized palliative care as a last resort, equating it with end-of-life care and considering it only when they could not provide active treatment. Only a few doctors believed palliative care could be complementary and integrated with active treatment.”* |
| Stewart, 2022 [15] | *“Currently 110 000 people in England living with ‘treatable but not curable’ cancer… The National Institute for Health and Care Excellence acknowledges the role of palliative care throughout the disease course alongside active anticancer treatment; however, barriers to referral and the association with ‘end of life care’ can restrict access.”* |
| Van Gurp, 2022 [12] | *“Policymakers could further integration by ensuring that palliative care teaching is widely available to new and junior healthcare professionals, so that they are instilled with a broader perspective on, and a different language about, care for patients with advanced and incurable diseases.”* |
| Lundeby, 2020 [11] | *“Everyone agrees on the main goal: to provide best possible care for the patients. … Interventions contributing to improved collaboration to reach this overall goal may counteract culture-biased attitudes and behaviour.… Resistance among HCPs should be anticipated but could be counteracted by facilitation of ﬁnding common ground and shared goals.”* |
| Abbreviations | C: Context  HCPs: Healthcare providers  M: Mechanism  O: Outcome |

| Leadership support | |
| --- | --- |
| **CMOC1b** | When organisation, system, or professional leadership demonstrate consistent support for the integration of palliative care (C), staff believe that palliative care is important to their institution/ profession (M), which motivates them to prioritise integration of palliative care (O). |
| Consistent support for the integration of palliative care is demonstrated to personnel where there is alignment in strategy, leadership actions, and investment [2]. Seeing their leadership as supportive encourages professionals to feel secure and motivated to carry out the work of integration, which is necessary for them to prioritise the potentially time-consuming tasks associated with integration within a busy healthcare setting [16, 17]. Deliberate alignment between system and clinical leadership and administration enables smooth implementation [18] and this becomes more likely where the mounting evidence base can be mobilised [19].  Leadership at different levels could take steps towards ‘promoting culture change’ [20] and creating ‘an institutional culture of acceptance’ [21]. Strategies include attending to funding, incentives, personnel, and physical space [9, 20, 21]; aligning informatics [18]; providing resources for training and continued professional development [22, 23]; and instigating collaborations between organisations and professionals across settings [6]. | |
| Illustrative quotes | |
| Garcia, 2023 [20] | *Higher-level leadership was primarily responsible for promoting culture change with messaging, funding, requirements, incentives, and stafﬁng… Higher-level leaders developed requirements and incentives tying metrics of serious illness communication to billing or bonuses for frontline team members, thereby signalling that it was of high institutional importance.”* |
| Garcia, 2023 [20] | *“Team members felt more inclined to participate fully when leaders modeled expected behavior (e.g., documenting conversations using appropriate forms, following prediscussed workﬂow protocol, etc.). Leaders who admitted to the team when they were wrong enabled others to feel safe doing the same, potentially fostering a learning health system environment.”* |
| Sadang, 2023 [18] | *“One respondent reﬂected on “the lack of leadership at the highest levels of the cancer center to support the delivery of telehealth” in which the clinician called for a “greater alignment between cancer center leadership, informatics department, administrative support, and clinic leadership before telehealth palliative care is implemented smoothly”.* |
| Schifferdecker, 2023 [9] | *“A strong organizational base for specialty palliative care, which includes personnel, systems, physical space, and rules or policies supporting or promoting palliative care, combined with social norms is necessary to support more integration… We posit that the presence of both produces a reinforcing cycle.”* |
| Bennardi, 2023 [24] | *“The influence of leadership motivates engagement with initiatives towards integration such as multidisciplinary rounds. Organisation culture and resource constraints might discourage cooperation between professionals – steps taken by the senior and middle management can be influential in creating the (practical and attitudinal) settings in which collaboration is more likely to occur.”* |
| Evans, 2019 [17] | *“The integrated care model was most successful under the following conditions: the model was embedded into existing workflows, electronic systems, and initiatives; time, funds, and human resources were dedicated to implementation; senior managers provided visible support and clear expectations for the model; and previous relationships/partnerships… were leveraged.”* |
| Abbreviations | C: Context  M: Mechanism  O: Outcome |

| Professional role clarification | |
| --- | --- |
| **CMOC1c** | When roles and responsibilities for delivering integrated palliative care are clearly defined (C), then professionals can understand what is expected of them and what they can expect from others (M), which facilitates communication and collaboration (O). |
| Working with uncertainty regarding professional responsibilities can lead to inefficiencies and confusion [25], in which crucial tasks become postponed [16]. For example, GPs might have a longstanding relationship with patients but become unclear on the scope or boundaries of their palliative care practice when oncology is also involved [26-29]. Healthcare professionals may be more willing and confident in engaging with ‘generalist’ palliative care tasks such as conversations about advanced care planning if they are clear on their role [29, 30].  Multidisciplinary collaboration requires an understanding of other professionals, such as their competencies, vernacular, schedules, and institutional processes [31]. When palliative care responsibilities are more clearly defined, then confusion is reduced because the professionals involved better understand their roles and can communicate these to others [32]. Providing role clarity thus enables collaboration and teamwork between disciplines towards improved patient care [33-35]. Some interdisciplinary teams could function better when there are flexible role definitions and shared problem-solving [6, 36], which requires communication to negotiate professional roles [20, 24]. | |
| Illustrative quotes | |
| Bennardi, 2022 [24] | *“‘Territorial boundaries" between primary care/oncology and PC are occasionally unclear or disputed. Previous evidence has shown that this can lead to conflicts… establishing strong professional guidelines, and clarifying competencies, represents a strategy to promote new professions (e.g., those related to PC), as all professions strive for "jurisdiction" over their field.”* |
| Gardiner, 2022 [37] | *“Our evidence supports the importance of defining roles… priority should be given to addressing how these challenges can be overcome in clinical practice to facilitate improved collaborative working between specialists and generalists… recent health policy… places generalist healthcare staff, and in particular nurses, at the centre of palliative care provision.”* |
| Hoek, 2022 [27] | *“Interprofessional boundaries: who is responsible for and should be involved in which part of the collaborative SDM process… explain the discomfort experienced with ‘meddling’ in decision making, as GPs need to negotiate the tension between ensuring the quality of decisions while at the same time preventing unnecessary doubts and confusion.”* |
| Bennardi, 2021 [32] | *“A lack of clarity around the tasks of the PC specialist and*  *those of the oncologist—and sometime, those of the GP. Some family members also experienced this lack of understanding…. A definition of roles and tasks from the start of the collaborative process avoids redundant services and multiplying roles.”* |
| Ferrell, 2021 [22] | *“To prepare oncology APRNs to perform an expanded role in the oncology workforce for integration of palliative care…. These APRNs will be pioneers in this palliative care generalist role, so that advanced practitioners in oncology care are fully able to address palliative care needs and oncology patients benefit from this.”* |
| Sommer, 2021 [28] | *“PCPs became more conscious of their role and responsibility during oncologic treatments... Overall, they discovered the role to help patients to make decisions for a better end-of-life… PCPs felt empowered and legitimated to take a more active role, and to keep contact with their patients during oncologic treatments.”* |
| Abbreviations | APRN: Advanced practice registered nurses  C: Context GP: General practitioner  M: Mechanism  O: Outcome  PC: Palliative care  PCP: Primary care provider  SDM: Shared decision-making |

| Societal perceptions of palliative care | |
| --- | --- |
| **CMOC1d** | When media or public health campaigns explain the benefits of integrating palliative care (C), then people with cancer and their families can better understand how they/their families could benefit (M), and so are more likely to accept or ask for palliative care (O). |
| Integration of palliative care is made more difficult by inaccurate understandings of palliative care in the wider community [38], indicating the need for efforts to destigmatise palliative care more broadly [39]. Addressing societal understandings of palliative care could enable openness to early integration, alleviating fears through more accurate knowledge of what it does and does not involve [7, 33, 40-42].  Since perceptions of palliative care are influenced by ‘a triad of culture, socioeconomic position, and health literacy’ [37], campaigns could be tailored to respond to variation in social and cultural meanings of health, illness and dying; for example, regarding sanctity of life [40], filial duty of care [7], or attribution of cancer [14]. | |
| Illustrative quotes | |
| Cerni, 2023 [43] | *“Patient awareness and understanding of palliative care was mentioned by the participants as being a key facilitator… “Education, education, education. People have this anonymity about palliative care. People think, oh they're just going to give you morphine and send you off to happy days. That's what we have to change.”* |
| Alizadeh, 2022 [40] | *“One of the challenges, is insufficient knowledge of the public and cultural barriers... Low level of knowledge in the public…can bring a negative impact on the acceptance of these services in the society. Therefore, increasing families’ awareness and changing their attitudes is recommended… radio and television can be very helpful.”* |
| Bennardi, 2022 [24] | *“Having a more precise image of palliative care is essential, not having an image related only to end-of-life, it’s true the fact that these services often arrive at that stage… this is related to the "stereotype" [of PC services, that they hasten death] which is perpetuated and the poor understanding.”* |
| Beiranvand, 2022 [33] | *“Participants pointed out the necessity of designing and implementing community awareness programmes with the aim of increasing knowledge, eliminating some irrational behaviours, changing attitudes, correcting misconceptions and gradually promoting the culture in this regard.”* |
| Gardiner, 2022 [37] | *“Misperceptions of the term ‘palliative’, alongside fear and stigma, contributed to a reluctance to engage with palliative care services, which could hinder the provision of effective support. Public perceptions of palliative care, and long held societal taboos around discussions of death and dying have undoubtedly contributed to this reluctance.”* |
| Perry, 2021 [44] | *“Emotionally, patients equate palliative care utilization with dying, giving up, welcoming the grim reaper, or being on the wrong side of “death panels”. These cognitive and emotional barriers govern patient reluctance to utilize early integrated palliative care.”* |
| Abbreviations | C: Context  M: Mechanism  O: Outcome  PC: Palliative care |

### Section 2: Cognitive participation (involving people in the work)

This section contains four CMOCs, shown in Box 2.

Box 2: CMOCs for section 2, cognitive participation (involving people in the work).

CMOC2a: Integration champions

When respected oncology professionals work in an integrated way (C), then their colleagues feel inspired to see integration as a positive thing to do (M), and so they are more likely to support or accept it (O).

CMOC2b: Professional buy-in

When oncology professionals are informed and listened to in the process of integration with palliative care (C), then they can feel reassured about the risks and potential benefits (M), and so become more likely to engage with integration (O).

CMOC2c: Identifying palliative needs

Where systematic ‘triggers’ for holistic needs assessment or referral to specialist palliative care are feasible and implemented within oncology practice (C), then professionals have clear and objective criteria with which to identify palliative care need (M), and so timely palliative care is more likely to occur (O).

CMOC2d: Introduction to palliative care

When people with cancer and their families have had their fears about palliative care addressed by a well-informed and trusted individual, (C), then they feel reassured (M), and so are more likely to engage with palliative care (O).

| Integration champions | |
| --- | --- |
| **CMOC2a** | When respected oncology professionals work in an integrated way (C), then their colleagues feel inspired to see integration as a positive thing to do (M), and so they are more likely to support or accept it (O). |
| Clinical culture is influenced by established hierarchy - the views and actions of a small number of people could set the tone for others [45]. Attitudes towards integration of palliative care within teams and disciplines could shift if there are trusted and respected individuals acting as ‘change champions’ [46]. For example, oncology nurses could champion integration within their interactions with both patients and physicians [8, 21].  Those who endorse a programme engage in efforts to ‘sustain and spread’ it within an organisation or system [2]. This is more likely to inspire action if it comes from a trusted source who understands the complexities of current practice [20, 21, 47]; although mentorship between institutions and countries could also contribute [47]. However, relying solely on a small number of individuals acting as change champions may lead to inconsistent implementation [46]. | |
| Illustrative quotes | |
| Krause, 2024b [13] | *“The passionate advocacy based on personal experience by the Emergency unit manager, staff and neurosurgery facilitated necessary resource allocation. Furthermore, this advocacy has contributed to the valuing of a PC approach and identifying the need for a PC service… PC integration is being driven by a small group of people.”* |
| Garcia, 2023 [20] | *“Discipline champions at the middle management level were selected to represent their discipline in team-based SICP implementation meetings, usually due to their passion for the program, how well respected they were among their peers, and/or their involvement in similar institutional efforts.”* |
| Rao, 2022 [46] | *“Local change champions are crucial in supporting transformative change efforts within an organization…. when changes are planned and executed by those within the organization then capacity building is sustainable and successful… To initiate, develop and implement any change you need change champions, both individual and organizational.”* |
| Satija, 2022 [47] | *“[PC-PAICE] remotely linked six US and Australian mentor sites with seven geographically diverse Indian mentee sites, four of which were tertiary cancer care hospitals while three were PC centers treating mainly patients with cancer. PC-PAICE aimed to improve Indian PC services through a mentored, stepwise, project-based QI learning program.”* |
| Bennardi, 2021 [32] | *“Providers describe promotion of their services as an essential aspect to foster cooperation—and identify a lack of time to devote to such promotion as a barrier to collaboration… Promotion of their own services, in particular PC services, and the relevant benefits can increase opportunities for cooperation. “* |
| Abbreviations | C: Context  M: Mechanism  O: Outcome  PC: Palliative care  PC-PAICE: Palliative Care – Promoting Assessment and Improvement of the Cancer Experience (a multisite international quality improvement initiative).  SICP: Serious Illness Conversation Program (involves nonphysicians team members in conducting serious illness conversations). |

| Professional buy-in | |
| --- | --- |
| **CMOC2b** | When oncology professionals are informed and listened to in the process of integration with palliative care (C), then they can feel reassured about the risks and potential benefits (M), and so become more likely to engage with integration (O). |
| Achieving clinician buy-in to the project could be instrumental in achieving its goals [17]. Oncologists who do not perceive the need for, or benefit from, palliative care integration are less likely to prioritise its integration [11]. Concerns around (for example) professional autonomy and clinical oversight should be understood and addressed - ideally the professionals involved in delivering change understand how integration is beneficial to them and their patients [23, 48].  Implementation strategies could prevent resistance to change by communicating iteratively and sensitively with clinicians [10, 23]. This could involve providing specific information on the collaborative service model [11, 48, 49]; supporting scientific evidence [21]; and/or reassurance regarding patient safety and capacity [50, 51]. | |
| Illustrative quotes | |
| Krause, 2024a [10] | *“Unwritten values and rituals must be tackled with sensitivity and understanding. For this reason, disruption of ‘how we always do things’ in an organization is not always welcomed.…. Stakeholders will not engage in PC interventions deemed inappropriate and unresponsive to their and their patients’ needs.”* |
| Lundeby, 2023 [23] | *“Project management prioritized thorough and iterative project information as an important part of the implementation process … The local PIs and members of the faculty presented the project and its elements to the staﬀ several times. Project communication also involved information about the ethical and practical conduction of the program.”* |
| Weinstein, 2022 [2] | *The project team members participated in work to sustain and spread the program within the organization. This included multiple educational sessions for staff, a midpoint survey of oncologists to assess their satisfaction with the program and solicit input on how to improve it.”* |
| Raunkiaer, 2020 [52] | *“If care model interventions are to be implemented without a deep understanding of what is already happening, they may be seen as being misguided and too resource intensive. … the users’ perspectives should be sought before the establishment of new and resource demanding palliative care services.”* |
| Adelson, 2017 [48] | *“Division leadership expressed their support for the study and oncologists unanimously agreed… Achieving buy-in for the intervention from oncologists, PC physicians, and hospital leadership was critical to both the success and sustainability of the intervention. Oncologists were shown the rationale for the intervention and given the forum to voice concerns.”* |
| Abbreviations | C: Context  M: Mechanism  O: Outcome  PC: Palliative care  PIs: Project investigators |

| Identifying palliative needs | |
| --- | --- |
| **CMOC2c** | Where systematic ‘triggers’ for holistic needs assessment or referral to specialist palliative care are feasible and implemented within oncology practice (C), then professionals have clear and objective criteria with which to identify palliative care need (M), and so timely palliative care is more likely to occur (O). |
| Uncertainty regarding the appropriateness of palliative care leads to ambivalence and delays [14, 53, 54] which prevent the full benefits of integration [55]. Hospital settings using automatic triggers have more consistent referral patterns [13, 56], because consistent and objective criteria facilitate decisions in the minds of professionals [26].  ‘Triggers’ for holistic needs assessment or specialist palliative care consult could serve to focus attention at key times such as advanced diagnosis, care transitions, emergency department attendance, or unplanned hospitalisation [4, 15, 18, 48, 57, 58]. To be feasible, triggers should be tailored to the local situation, for example being easy-to-use within existing patient record systems and identifiable without additional screening [17, 59]. Although capacity could be constrained by human resources [60, 61], systematic triggers have been used internationally in both outpatient [42, 61, 62] and inpatient settings [26, 48]. | |
| Illustrative quotes | |
| Krause, 2024 [10] | *“The provision of collaborative care is conceptualized as the appliance of automatic triggers… VNLDS service has initiated automatic triggers in patients with pancreatic adenocarcinoma and end-stage renal patients, which are reflected in the referral patterns. …Automatic triggers specific to the context are thus core to further integration and universal PC.”* |
| Raunkiaer, 2023 [55] | *“Lack of screening for palliative needs early in disease trajectories and lack of clear referral criteria for SPC delayed referral… ‘It may well be that they do not need much PC at that time, but if they are inside the healthcare system when the need comes, the support is there.’”* |
| Collins, 2022 [59] | *“Electronic prompt to clinicians could serve as a reminder, reducing clinical uncertainty and reinforcing the service expectations… whilst not reducing physician agency. An effective trigger-prompt system would be one where clinicians are reminded of palliative care beneﬁts and retain the decision making about how and when those are best enacted.”* |
| Brenne, 2021 [50] | *“A standardized care pathway is a method to improve quality and reduce variation in healthcare. It can promote integrated healthcare services in palliative care, e.g. by specifying action points when the patient’s situation is changing … An educational program and an information strategy were developed to ensure implementation.”* |
| Yang, 2018 [63] | *“Sometimes you wonder if we [oncologists] are referring too early and if we are over-burdening our colleagues [in palliative medicine] ... The last thing you want to do is to tax the system.” This led to widely varying referral practices in the usual inpatient consult service.* |
| Abbreviations | C: Context  M: Mechanism  O: Outcome  PC: Palliative care  SPC: Specialist palliative care  VNLDS: Vertical nurse-led doctor-supported service (model for integration) |

| Introduction to palliative care | |
| --- | --- |
| **CMOC2d** | When people with cancer and their families have had their fears about palliative care addressed by a well-informed and trusted individual, (C), then they feel reassured (M), and so are more likely to engage with palliative care (O). |
| People with cancer or families might resist the involvement of palliative care because they do not understand how it can be of relevance to them or if they perceive it could have negative ramifications for their care [21, 27]. The way in which clinicians communicate about palliative care is highly consequential to how it is perceived [42, 64].  Trusted individuals have an important role in explaining the benefits of involving palliative care [37, 56]; e.g., emphasizing its merit for symptom management and improved quality of life [12, 21, 65]. If (all) healthcare professionals were well-informed about the (modern) meaning of palliative care, then they would be equipped to reassure patients and caregivers by addressing their fears and misunderstandings [24, 41, 42, 65, 66]. | |
| Illustrative quotes | |
| Montiel, 2023 [67] | *“The usefulness of services must be explained to them and avoid medical jargon; sitting with them and presenting, multiple times, the benefits of the different services could be helpful…. Help-seeking behaviors can be affected by cultural, social, and structural barriers such as trust in healthcare professionals, language, or racism.”* |
| Okyere, 2023 [14] | *“Misconceptions about PC led to non-compliance when patients are referred for PC…. Patients with poor knowledge and wrong perceptions about PC would want to assume a state of denial and explore all other options available to them before considering PC. This could significantly delay the initiation of PC.”* |
| Sullivan, 2023 [21] | *“Overall acceptance by patients and families was thought to be a minor barrier to integration, as most clinicians felt the majority of patients appreciated the services when the goals were explained. Alternatively, poor framing by the referring clinician of the role of PC was reported to signiﬁcantly reduce patient acceptance.”* |
| Zimmermann, 2023 [56] | *“Education about palliative care by the nurse conducting the phone call was perceived to be helpful to alleviate patients’ concerns: ‘since the nurse had explained to me that [palliative care] is more than just (…) end of life, I decided that okay, maybe I’ll go.’ (Patient)”* |
| Collins, 2022 [65] | *“Health professionals may lack the conﬁdence or skill to describe the role and beneﬁts of EPC…. compounded by variable levels of community understanding about palliative care, with perceptions of relevance only for those imminently dying…. Upskilling clinicians around communication of this topic will be important to ensure successful implementation.”* |
| Van Gurp, 2022 [12] | *“Seriously ill patients are not necessarily convinced of the value of palliative care…. To find an entrance with patients and keep their trust, the tactful timing of communicating difficult truths together with introducing a palliative care perspective is key.”* |
| Abbreviations | C: Context  EPC: Early palliative care  M: Mechanism  O: Outcome  PC: Palliative care |

### Section 3: Collective action (working together and becoming a team)

This section contains six CMOCs, shown in Box 3.

Box 3: CMOCs for section 3, collaborative action (working together).

CMOC3a: Regular communication

When there is regular and reciprocal communication between multidisciplinary professionals within and between different care settings (C), then trust, respect, and mutual understanding can develop over time (M), which facilitates effective collaboration (O).

CMOC3b: Collaborative decisions

Where norms and routines encourage safe and respectful sharing of different perspectives between and within multidisciplinary teams (C), then team members feel encouraged to contribute their perspectives and knowledge (M), and decision-making becomes more holistic (O).

CMOC3c: Coordination of care

When the responsibility for coordinating and planning care is assigned to a capable and credible professional (C), then the different professionals and patients and caregivers have a shared understanding of what is needed (M), and so consistency and continuity is more likely to occur (O).

CMOC3d: Information needs

When individualised needs for information can be met over time in a person-centred manner (C), then people with cancer can better understand their illness, treatment, and care options (M), and so become more able to participate in shared decision-making and planning ahead (O).

CMOC3e: Caregiver support

When integrated palliative care includes honest and sensitive communication and education for caregivers (C), then caregivers can feel supported and less anxious (M), and more able to cope in their role (O).

CMOC3f: Safety at home

When integrated palliative care involves planning for the home environment and providing access to responsive support (C), then people with cancer and their families can trust that help is available if needed (M), so they feel safer and more able to manage at home (O).

| Regular communication | |
| --- | --- |
| **CMOC3a** | When there is regular and reciprocal communication between multidisciplinary professionals within and between different care settings (C), then trust, respect, and mutual understanding can develop over time (M), which facilitates effective collaboration (O). |
| Frequent, proactive and reciprocal communication allows professionals to feel informed and understanding of other team members, which encourages further collaboration via mutual knowledge and the integration of different perspectives [16, 24]. Face-to-face discussion enables consensus during decisions, care planning and coordination [55, 68, 69]. One-way sharing of written information is not sufficient for professionals in different settings/disciplines to develop mutual understanding and trust [6, 31, 32]. Formalised arenas for in-depth discussion might be necessary for professionals to integrate different perspectives and build trust in collaboration [11, 32, 70].  Strategies that provide structure and routine for collaboration (such as co-location, co-rounding, daily huddles, multidisciplinary clinics, virtual transmural meetings [63, 70-73] have been described as providing the ‘currency’ for developing collaborative relationships between clinicians[21]. Logistical challenges impede communication between specialists organised in separate spaces [9]. Although technology might contribute [18, 74] it is not a panacea [31]. Palliative care being seen as a ‘true member of the multidisciplinary team’ is aided by a physical presence in the oncology environment, at least partly because this allows additional ad-hoc communication [21, 31, 32, 51]. | |
| Illustrative quotes | |
| Bakken, 2023 [16] | *“Nurses asked for debrief meetings with doctors to learn from different situations, and to gain a better understanding of each other’s work… “An interdisciplinary meeting where we could have talked together. But it is difficult to achieve, it is a very busy unit, it is difficult to find the time.””* |
| Cushen-Brewster, 2023 [71] | *“Some participants stated that communication with the wider team or community was effective and that all parts of the team worked well together. This was perceived as being, in part, a result of regular team meetings: ‘We have a daily huddle where complex palliative patients are discussed.”* |
| Sullivan, 2023 [21] | *“Clinicians reported that the most meaningful facilitators of integrated PC across patient, clinician and hospital levels were the importance of a physical presence and colocation of PC services to develop trusting relationships, particularly with oncologists, enhancing collaboration in outpatient settings…Interpersonal relationships and frequent communication were important for success.”* |
| Ervik, 2023 [75] | *“Oncology nurses and GPs organized small teams around palliative patients and their families, which allowed them to use their complementary expertise in discussing expected or potential changes in the patient’s situation…. where local HCPs, patients and families could have a mutual understanding of feasibility with available resources.”* |
| Dhollander, 2022 [70] | *“We included one additional component compared with previous interventions, that is, structured interprofessional collaboration. Transmural and interprofessional collaboration was added as an additional component. Previous interventions have mentioned that communication between oncologists and palliative care professionals is important but did not explicitly include it as a component of the intervention.”* |
| Johansen, 2022 [31] | *“Frequent informal communication was regarded as the most important factor in effective collaboration. These brief exchanges were necessary for creating shared knowledge, shared goals, and shared clinical decision-making. ‘Favourable physical space configuration’ and ‘having frequent brief time in common’ were key facilitators.”* |
| Johansen, 2022 [31] | *““A lack of meetings could mean that clinicians did not have common goals for their palliative care, and thus no team approach. They perceived a need to physically meet to routinely share information, assessments, and evaluations of patients’ situations and discuss how this was related to their own work. “* |
| Lundeby, 2020 [11] | *“Both nurses and residents requested speciﬁc arenas for collaboration. They argued that increased collaboration would beneﬁt the patients by, for example, improved information ﬂow regarding symptoms and patient preferences… Communication is a key element of collaboration to obtain trust, balance power and clarify professional roles between healthcare professionals.”* |
| Abbreviations | C: Context  HCPs: Healthcare professionals  GPs: General practitioners  M: Mechanism  O: Outcome  PC: Palliative care |

| Collaborative decisions | |
| --- | --- |
| **CMOC3b** | Where norms and routines encourage safe and respectful sharing of different perspectives between and within multidisciplinary teams (C), then team members feel encouraged to contribute their perspectives and knowledge (M), and decision-making becomes more holistic (O). |
| Professional hierarchies can mean that multidisciplinary team members lack agency to contribute their views [11, 16, 20]. Restrictive mandates or presumptions for clinical decision-making within oncology can reduce discussion and reflection [32, 76] and may cause unnecessary delays [20, 25]. In contrast, a more collaborative dynamic could be empowering to the team [8, 10] and beneficial to decisions made, such as on treatment appropriateness [77, 78]. Seeking and valuing the input of other team members facilitates interdisciplinary work and for decisions that are better informed about the person with cancer’s needs and preferences [64, 79].  Social norms towards collaborative decision-making relate to how palliative care is perceived in relation to oncology, which suggests initiatives towards familiarity and networking [9]. Educational strategies include interdisciplinary training and professional development opportunities that develop collaboration and negotiation skills [24, 80] (e.g. Schwartz rounds [21]). | |
| Illustrative quotes | |
| Krause, 2024 [10] | *“It’s more the doctors that are giving the orders. There’s no multidisciplinary approach. There’s no, like a combined view on the patient… The nurse will accompany the doctor and take the orders. (Pallnurse2) “Yes, so… I don’t think the nursing staff feel empowered enough to actually make suggestions.” (Wardstaff1)* |
| Cushen-Brewster, 2023 [71] | *“More meetings were implemented to improve collaborative working… ‘The SPC team are very much wanting us to be involved and they ask us what we think as well as just telling us what they want done.’ (Community service-provider).* |
| Schifferdecker, 2023 [9] | *“We encourage sites interested in promoting palliative care integration to assess strategies and communication which acknowledge social norm influences and potentially address them, such as assessing who is “allowed” to initiate goals of care conversations or feedback from patients on whether their goals were ascertained, when and by whom.””* |
| Thelen, 2023 [64] | *“Teams need to explicitly consider and agree their mode of functioning, and enact changes to enhance knowledge of the team, intentional communication and valuing other teams’ contributions…. ‘Communicating intentionally’ was enhanced when disciplinary hierarchies were not entrenched and communication from any discipline between teams was welcomed.”* |
| Bennardi, 2022 [24] | *“Training should be used to improve interdisciplinary collaboration. This training should include formal education (e.g., technical and professional trainings), informal education (e.g., learning from peers/colleagues or mass media) and non-formal education (learning from environment or experience), as these methods strongly influence not only skills, but also attitudes towards incorporating PC.”* |
| Ribi, 2022 [80] | *“A conscious, consensually agreed and lived culture of interprofessional collaboration including transprofessional education that is institutionally supported and ﬁnanced, may substantially improve the quality of decision processes. This includes strengthening the role of oncology nurses in decision-making processes.”* |
| Ullgren, 2022 [25] | *“SPC professionals stated that they were working by the “order” of the acute oncology team, without a mandate to change, question, or not knowing the goals of care… Absence or delay of clinical decisions was frequently reported... and seemingly led to unnecessary ER visits and unplanned hospital admissions.”* |
| Ellen, 2021 [77] | *“As members of [MDTs], nurses can provide a vital contribution to effective and efficient teamwork but can lack the opportunity to articulate their clinical opinions in a physician-centric environment. Understanding and addressing these dynamics can lead to development and implementation of interventions encouraging nurses to express their professional opinions.”* |
| Abbreviations | C: Context  ER: Emergency room  M: Mechanism  MDTs: Multidisciplinary teams O: Outcome  SPC: Specialist palliative care |

| Coordination of care | |
| --- | --- |
| **CMOC3c** | When the responsibility for coordinating and planning care is assigned to a capable and credible professional (C), then the different professionals and patients and caregivers have a shared understanding of what is needed (M), and so consistency and continuity is more likely to occur (O). |
| Coordination is important due to the number of professionals and settings potentially involved in integrated palliative care and oncology [6, 10, 81]. Uncoordinated care leads to gaps, such as medication or equipment delays, which increases burden on people with cancer and their families/caregivers, who then struggle to navigate the system to address these gaps [27, 31, 82]. Coordination during care transitions is particularly important [31, 52, 83]. Clarity over who is in charge and the guidance received is reassuring for patients and families [84] and could help to avoid negative outcomes [2, 85].  Where integration palliative care increases the number of appointments, these require planning to prevent clashes of clinical opinion or scheduling [18, 73, 81, 86, 87]. Visibility of the coordinator with the relevant clinicians helps them to establish credibility [2].  Collaboration requires labour towards coordination, to maintain communication and ensure consistent information sharing [12]. Research conducted within a trial may have additional resources such as study coordinators that act to facilitate the coordination of care [18]. | |
| Illustrative quotes | |
| Krause, 2024 [10] | *“The service also follows up… 1 week after discharge… “I do a follow-up with the patient to see if they got the community key services; then if not, I will follow-up with the coordinator working in that specific area where the patient stays to find out the delay.” (Admin PC)”* |
| Cushen-Brewster, 2023 [71] | *“Participants reflected on how coordinated they found the service… Patients and carers interviewed all felt that the care they received was very well coordinated: ‘Everything was in place, everything was done very efficiently. We did not have to wait for anything. Everything kicked-in very efficiently, it was just very good.’”* |
| Mashiro, 2023 [6] | *“The function of coordination among facilities is unclear, and some patients do not have a primary care physician or care manager to take on the coordination role… it is unclear who is responsible for this coordination role… A profession or institution must be established as the center of coordination.”* |
| Sadang, 2023 [18] | *“A sustainable, patient-centered EIPC delivery system must strive to minimize patient trips and consolidate appointments… Use of study coordinators were also highly agreed upon facilitators… [as] part of a larger research trial… It is unknown if clinic support staff in the real-world setting would have the same capacity or effectiveness.”* |
| Thelen, 2023 [64] | *“Patients received more coordinated care, meaning that care plans developed in one setting carried over to the other and were mutually understood and agreed upon by clinicians in each setting… Some teams identified one member as the ‘bridge’ between teams to facilitate communication.”* |
| Weinstein, 2022 [2] | *“Fully integrating the care coordinators within the primary oncology team had multiple advantages. This approach required a significant investment of staff time, but… there were numerous occasions in which the care coordinator was able to intervene early in a patient problem, preventing avoidable ER visits or improving symptom management.”* |
| Abbreviations | C: Context  EIPC: Early integrated palliative care  ER: Emergency room  M: Mechanism  O: Outcome  PC: Palliative care |

| Information needs | |
| --- | --- |
| **CMOC3d** | When individual needs for information can be met over time in a person-centred manner (C), then people with cancer can better understand their illness, treatment, and care options (M), and so become more able to participate in shared decision-making and planning ahead (O). |
| People with cancer who do not understand their illness might continue to receive treatment until late in their disease trajectory [11, 79] or struggle to acquire reliable information about their options [88]. Anticipation of realistic outcomes can go awry if goals of care are not understood [80]. When patients receive information about their illness and dialogue on realistic outcomes, this equips them to consider their treatment and care preferences [10, 20, 77, 89].  Conversations held gradually but more often could be helpful in meeting informational needs, recognising fluctuations in readiness and being able to take on-board the information [1, 56, 90]. Individualised information provision appears more likely in situations where there is a relationship with a known professional [79, 91], who can tailor how and when to share information [12, 65] and empower patients to make their own informed decisions [8, 77]. Well-designed informational resources, such as booklets or videos, could aid the work of stimulating reflection and discussion [44]; but might act as an adjunct rather than a replacement to the contribution of (e.g.) nurses in leading discussions [92]. | |
| Illustrative quotes | |
| Maessen, 2024 [58] | *“As a result of the SENS intervention, patients and their families understand and prepare for controlling symptoms and make relevant treatment and care decisions for redirecting care towards quality of life and function, which may eventually reduce emergency hospitalisations.”* |
| Mensah, 2023 [87] | *“[Patients] expected that receiving palliative care services would offer them a deeper understanding of their situation and prognosis to assist them to make informed decisions and plans about treatment choices…. A recurring pattern was the expectation to receive meaningful information and communicating about the disease condition and the prognosis.”* |
| Ervik, 2023 [75] | *‘There’s a big difference between patients you’ve followed over time and had contact with throughout… you talk to them about the end and how they want it and what they think about… Then it’s much easier the day there’s a crisis, because then you’ve made a kind of plan...’ (GP)* |
| Roberson 2023 [88] | *“Participants frequently felt confused about, and frustrated because of, the lack of information available about their diagnosis, prognosis, and treatment options... Participants felt that physicians did not help them make connections to reliable and easily accessible information, leaving participants feeling solely responsible for their own empowerment through information acquisition.”* |
| Zimmermann, 2023 [56] | *““I’m really like fortunate that [palliative care physician] help me understand about my symptoms and about my cancer…” [Patient]. “It reminds me that despite having cancer, I can still have a good opportunity to live my life to the fullest, not experience too much pain, and have additional information.” [Patient].”* |
| Traeger, 2020 [90] | *“Clinicians encouraged patient and family contemplation about quality-of-life implications of continuing treatment or the possibility that treatment might harm more than help, in anticipation of a change in health status…. longitudinal palliative care relationships may function through multiple pathways to support patients and families in making complex EOL care decisions.”* |
| Abbreviations | C: Context  EOL: End of life  GP: General practitioner  M: Mechanism  O: Outcome  PC: Palliative care SENS: Structured multiprofessional conversation with the patient about symptoms, end-of-life decisions, network building and support for carers. |

| Caregiver support | |
| --- | --- |
| **CMOC3e** | When integrated palliative care includes honest and sensitive communication and education for caregivers (C), then caregivers can feel supported and less anxious (M), and more able to sustain the level of caregiving they wish to fulfil in their role (O). |
| Early integration of palliative care that prepares caregivers could enable them to continue in carrying out their role [93]. Family education could include discussions over time addressing capacity to cope at home [10, 90]. Including caregivers in communication, where appropriate, helps to establish a collaborative team around the patient [94]. In contrast, caregivers who are not prepared by the health system experience more anxiety, especially out-of-hours [79] and are more likely to seek help from hospital or emergency services [62, 93].  Strategies include family meetings [55, 58, 90], ad-hoc ‘training moments’ from nurses [94], and courses on home care skills [36]. | |
| Illustrative quotes | |
| Krause, 2024 [10] | *“Patient and family education… is both clinically and organizationally important... Family education [is] a positive contribution to care and empowers the family to continue on their own…. Family meetings are thus core to assessing family structure, support and educating patients and families on the required care.”* |
| Ervik, 2023 [75] | *“Family carers were essential to allow patients to die at home, nurses spent a great deal of time and energy informing and advising family carers to help them to feel secure in their caring role, and… to observe changes in the patient’s situation and know when to call for help.”* |
| Mensah, 2023 [87] | *“Family caregivers are often confused about what to do when [the patients] are experiencing symptoms at home. Therefore, it was expected that palliative care would provide an opportunity for the family caregivers… to be provided with adequate information about the patients’ condition and the caring role that must be delivered.”* |
| Spelten, 2021 [85] | *“Safety at home with HPC often require support from family and friends… HPC can be hard on the family, as it substantially limits privacy and time to rest…Easier access to guidance, counselling, and assessments may explain the reduced need of emergency care and hospital admissions during HPC in our data.”* |
| McCaughan, 2019 [79] | *“Relatives, often anxious due to lack of overnight support, were described as likely to contact out-of-hours’ services, or the haematology ward if the patient's condition deteriorated, resulting in patients discharged home for end-of-life care ‘bouncing’ back into hospital.”* |
| Collins, 2013 [93] | *“The limited preparation by the health system to assist carers to effectively undertake their role was emphasised. They felt unsure of how to enlist support afterhours, often defaulting to emergency departments for simple advice…. ‘every time I hit that bump I just didn’t have that ‘go to’ person to ask.””* |
| Abbreviations | C: Context  HPC: Home palliative care  M: Mechanism  O: Outcome |

| Safety at home | |
| --- | --- |
| **CMOC3f** | When integrated palliative care involves planning for the home environment and providing access to responsive support (C), then people with cancer and their families can trust that help is available if needed (M), so they feel safer and more able to manage at home (O). |
| Anxiety about cancer and its symptoms, coupled with a lack of available alternatives for help, seems to push people towards hospital-based care [95]. This could be avoided if further attention was paid to wellbeing and perceived safety at home, including pain and symptom management, and access to appropriate escalation routes [6, 85]. Improved forward planning and advice on interpreting symptoms is reassuring [20, 95, 96]. Plans can anticipate likely changes and act to prevent crises [75] but responsiveness to fluctuating need remains important [89].  Straightforward access to a known person or team encourages the trust of patients and families [34, 55, 71], especially if contact is proactive and established prior to being in crisis [56, 67]. This could allow questions to be answered or support needs identified without having to default to A&E [93]. Therefore, making it easier to access and re-access support in the home environment is valuable in preventing A&E attendance and hospitalisation in the last year of life [40, 97]. | |
| Illustrative quotes | |
| Cushen-Brewster, 2023 [71] | *“Total of 19712 telephone calls to the OneCall 24-hour advice line were recorded. The highest proportion of these calls were from carers or relatives (46%), followed by patients (22%) … Patients and carers reported that they received high-quality care and valued having easy access to members of the team when needed.”* |
| Ervik, 2023 [75] | *“Although most patients prefer to die at home, this requires a certain level of competence and resources, and the patient, family carers and HCPs must have a feeling of safety and security… HCPs described trying to anticipate a potential crisis to avoid the necessity of e.g. calling a helicopter.”* |
| Raunkiaer, 2023 [55] | *“The third suggestion was A direct phone line, established with palliative resource persons/ teams, that the patients and relatives could call when they arrive home, even if they did not have immediate PC needs: ‘It is a huge security for the patients to have someone to call.”* |
| Walshe, 2023 [89] | *Walshe = ‘Responsiveness’ was evident in… the ability to identify and respond to individual patient needs; and the ability to respond in a timely manner to these changing needs. Patients and family carers were satisfied with the services which appeared to increase their confidence and help them to be less fearful.”* |
| Zimmermann, 2023 [56] | *“I was just wondering if this is how things are and they’re going to get even worse than this, how will I manage and stuff. So, yeah, getting in touch with them and talking and finding out about different resources to handle the situation was very helpful, yeah.” (Patient)* |
| Beiranvand, 2022 [33] | *“The lack of a formulated system for addressing these primary needs leads to frequent revisits to specialty and super-specialty centres, hospitalisation in emergency rooms or intensive care units and the imposition of treatment costs and unnecessary measures on patients and the health system.”* |
| Spelten, 2021 [85] | “*Symptom management was seen as the main reason for hospital admission in both [FGs]. It was felt that appropriate and adequate interventions could prevent this, but the timely provision of medication was perceived as a barrier… Pain relief, medication and symptom control are seen as a major cause for hospitalisation*.” |
| Abbreviations | C: Context  FGs: Focus groups HCPs: Healthcare professionals  M: Mechanism  O: Outcome  PC: Palliative care |

### Section 4: Reflexive monitoring (considering how well it is working)

This section contains four CMOCs, shown in Box 4.

Box 4: CMOCs for section 4, reflexive monitoring (considering how well it is working)

CMOC4a: Evaluation and feedback

Where initiatives towards integrating palliative care within oncology are monitored, meaningfully evaluated, and reported on (C), then stakeholders can understand the value of working in this way (M), and their support for integration is more likely to be sustained (O).

CMOC4b: Sharing expertise

When ‘generalist’ palliative care professionals work collaboratively and constructively with ‘specialist’ palliative care professionals (C), then these ‘generalists’ feel more confident (M), leading to increased capacity for palliative care (O).

CMOC4c: Workforce wellbeing

When healthcare professionals receive support through their collegiate relationships in facing the ethical and emotional challenges of their role (C), then they feel less isolation and moral distress (M), leading to improved workplace satisfaction (O).

CMOC4d: Improved decision-making

When professionals, patients and caregivers have a realistic and shared understanding of the patient’s needs, goals and values (C), then the team are empowered to make informed decisions, (M), which means timely palliative care provision is more likely (O), and unnecessary or avoidable resource use is minimised (O).

| Evaluation and feedback | |
| --- | --- |
| **CMOC4a** | Where initiatives towards integrating palliative care within oncology are monitored, meaningfully evaluated, and reported on (C), then stakeholders can understand the value of working in this way (M), and their support for integration is more likely to be sustained (O). |
| Integration is impeded when performance indicators are not aligned with the goals of integration [2, 21]. Without knowledge of the outcomes, it could be more challenging to see the value of striving towards integration [10]. A commitment to system learning helps to mobilise and inform efforts towards continued improvement [2]. This could involve embedding evaluation and consistently providing feedback to teams, for example as end-of-life metrics tracked to quality improvement initiatives [9]. However, identifying appropriate quality indicators requires a shared vision of ‘how to measure successful integration’ [98].  Data can inform planning, evaluation and refinement of initiatives [99] and indicate priority areas where socioeconomic conditions present additional barriers [100]. Tracking of milestones, enablers, and barriers provides insight that informs the spread of good practice [17]. Positive patient feedback also helps demonstrate improved care to stakeholders [71]. | |
| Illustrative quotes | |
| Krause, 2024 [10] | *“One of the aspects of organizational integration is that there are monitoring and evaluation tools available to evaluate the PC… integrating an intervention into the broader health system governance structure will require alignment with existing regulatory mechanisms, creating unified accountability frameworks, integrating reporting and establishing a joint performance management system.”* |
| Krause, 2024 [13] | *“In a hospital striving for excellence, poor outcomes are not welcomed. Therefore, if PC outcomes are not measured as an indicator of good care, the need to strive for good PC outcomes may seem unnecessary and counterproductive. Including and measuring PC outcomes as measures of excellence may strengthen accountability.”* |
| Lundeby, 2023 [23] | *“Weekly meetings to discuss and evaluate progress, implementation strategies, share experiences … close contact with the local PIs about their experiences and feedback they had received. Some PIs sent written evaluation reports to the faculty. The project management, faculty, and all local PIs met once a year to discuss practices”* |
| Rao, 2022 [46] | *“The first cycle was followed by group reflective sessions…. to better understand the barriers and enablers and modify the action plan for the second cycle… This reflective cycle enabled identification of components that facilitated and impeded palliative care capacity building in the cancer treatment institutes and facilitated corrective actions.”* |
| Weinstein, 2022 [2] | *Implementing a system-wide program such as LINCC requires deliberative alignment with existing system strategic planning… and a commitment to system learning… This can position organizations for success… if the organization has learning systems in place to understand the information and subsequently mobilize the organization through quality and process improvement initiatives.”* |
| Hoverman, 2020 [100] | *“The change in payment structure and the continual review of quality metrics have focused our attention on end-of-life care… A promising aspect of the OCM is the interrogation of data at multiple sites both in and out of USON to uncover and share best practices.”* |
| Abbreviations | C: Context  LINCC: Learning Individual Needs and Coordinating Care (integration program)  M: Mechanism  O: Outcome  OCM: Oncology care model (Medicare palliative care program)  PC: Palliative care  PIs: Project investigators  USON: US Oncology Network |

| Sharing expertise | |
| --- | --- |
| **CMOC4b** | When ‘generalist’ palliative care professionals work collaboratively and constructively with ‘specialist’ palliative care professionals (C), then these ‘generalists’ feel more confident (M), leading to increased capacity for palliative care (O). |
| Greater generalist and specialist collaboration, achieved through closer working patterns, provides opportunities for multidisciplinary professionals to learn from each other, and build confidence in their own and each other’s skillsets [29, 43, 71, 101]. Reduced professional contact since Covid-19 may have interrupted opportunities to share expertise [10, 71] and specialist palliative care staff who are working over capacity are less able to support others [89]. Since the extension of specialist palliative care teams to meet all demand is unlikely, close attention to the responsibility and skillset for generalist palliative care is necessary.  Strategies involve a combination of formal training sessions and experiential learning (‘on-the-job’ exposure to palliative care skills) [10, 22]. ‘Train the trainer’ models foster the expertise of non-specialist staff to continue to develop the skills of others [22]. Technology could be useful for networks of expertise sharing, for example linking academic hospital specialists with community providers [6, 52, 85, 102].  Some reports recommend restructuring community roles via task-shifting to adapt to workforce shortages [46, 85, 103], corresponding to an extension of roles at the ‘specialist-generalist’ interface (e.g., advanced practice nurses, allied health professionals, care navigators, community nurses, community paramedics) [22, 43, 85]. Other professionals that could be empowered in their contributions include psychologists, nutritionists, social workers, physiotherapists, dieticians, community health workers [53, 97, 104]. | |
| Illustrative quotes | |
| Krause, 2024 [10] | *“VNLDS provided a quarterly lecture as continuing education on PC open to the hospital but this stopped during COVID-19. There is an acknowledgement that there is a need to provide further continued education in PC across the hospital: ‘There needs to be more empowerment of the current team’ (Pallnurse2)”* |
| Bakken, 2023 [16] | *“Timely recognition of EOL requires knowledge, experience, courage, and good clinical judgment…. Doctors also expressed the need for collegial support and discussions with other, more experienced doctors…. Participants also asked for time to evaluate and learn from nonoptimal situations, time for guidance through teaching and collaboration with experienced colleagues”* |
| Cerni, 2023 [43] | *“Ongoing professional development and upskilling of GPs and specialist health care professionals was considered as an effective way to improve the confidence of health professionals… Greater generalist and specialist interdisciplinary collaboration… could assist more rural-based GPs to take on an expanded role…with support from palliative and cancer care specialists.”* |
| Cushen-Brewster, 2023 [71] | *“The specialist palliative care service promoted collaborative working, leading to enhanced knowledge and skills for staff … ‘It has made a great difference; the staff have learned so much from them. They share their knowledge [and] protocols and you can see how that makes a difference to patient care.’”* |
| Van Gurp, 2022 [12] | *“Optimal integrated palliative care is still a work in progress. The interviewees, however, emerged as modest, patient, and highly motivated teachers. … In current healthcare systems, palliative care professionals have to develop a teaching role while remaining diplomats fostering palliative care being part of regular healthcare and healthcare education.”* |
| Ferrell, 2021 [22] | *“Nurses reported on the value of spending time with the palliative care service to continue to expand their knowledge and skills… The reported feeling was that this was a valuable learning experience.…APRNs benefitted from observing the clinical skills and communication expertise of the palliative care specialists.”* |
| Yang, 2021 [105] | *“The proximity of specialist palliative care resources in the co-rounding model meant that advice could be given to enhance the quality of generalist palliative care provided by the oncology team… The co-rounding model could facilitate opportunistic palliative care education for the oncology team.”* |
|  | APRNs: Advanced practice registered nurses  C: Context  EOL: End of life  GP: General practitioner  M: Mechanism  O: Outcome  PC: Palliative care  VNLDS: Vertical nurse-led doctor-supported service |

| Workforce wellbeing | |
| --- | --- |
| **CMOC4c** | When healthcare professionals receive support through their collegiate relationships in facing the ethical and emotional challenges of their role (C), then they feel less isolation and moral distress (M), leading to improved workplace satisfaction (O). |
| Healthcare professionals such as oncologists have an innately stressful role in caring for advanced cancer patient, involving ethical dilemmas in which there are no ‘good options’ to meet both principles of patient autonomy and non-maleficence [106]. Working alone with this emotional burden has an impact on their wellbeing, contributing to distress, burnout and detachment/complacency [29, 76, 79]; which is a risk for patient safety [85] and system efficiency [20]. Community settings also pose a heightened risk of emotional burden due to isolated working [102].  Collaboration within cancer care could reduce ethical dilemmas and isolation experienced by staff [20, 31], allowing professionals to share and reflect on the emotional burden [24, 54, 107]. Opportunities to debrief on nonoptimal scenarios alleviates moral distress that might arise for staff [13, 16, 85]. Coaching and emotional support is suggested to be an important contribution of specialist palliative care to the integrated team [10, 76, 107, 108], with potential to improve staff morale and possibly retention [12, 89]. | |
| Illustrative quotes | |
| Krause, 2024 [10] | *“Open engagement with the suffering and actual dying process has assisted in addressing the staff’s personal moral distress… Vulnerability around death and dying is respectfully acknowledged and managed in this ward. There is thus a realization of the benefit of PC service through attending to the staff’s needs.”* |
| Krause, 2024 [13] | *“Feelings of failure and hopelessness are common findings among HCPs… It is evident from multiple managers that there is an element of moral distress associated with working with patients requiring PC…. without the skills to care for patients with life-threatening illnesses, there is over-investigation, avoidance and even hopelessness among HCPs.”* |
| Bakken, 2023 [16] | *“Nurses explained how they felt that they had to administer futile medication and treatment that made the patient suffer unnecessarily, instead of contributing to a peaceful death… Nurses and doctors described a feeling of responsibility to advocate for the patients in a rigid healthcare system [and] a feeling of helplessness.”* |
| McPherson, 2023 [54] | *““Poor teamwork and team support, such as difficulties in maintaining open communication and a collaborative environment, may also lead to moral distress among healthcare professionals… collaborative and respectful relationships and opportunities to have confrontations and exchange of experiences may help healthcare professionals to cope with moral distress.”* |
| Bennardi, 2022 [24] | *“That would be a mutual exchange, an "unloading"… it is a burden to carry alone, and if you share it from the start, it is easier (NPC – Oncologist).”* |
| Johansen, 2022 [31] | *“When working so close to each other, no issue ever got ‘old’; if clinicians were wondering about something, they could bring it up immediately…. Contact was easy and informal, which they regarded as important. The co-located ONs talked about how they appreciated the support and opportunities for debriefing sessions.”* |
| Abbreviations | C: Context  HCPs: health care professionals  M: Mechanism  NPC: Not specialist palliative care O: Outcome  Ons: Oncology nurses  PC: Palliative care |

| Improved decision-making | |
| --- | --- |
| **CMOC4d** | When professionals, patients and caregivers have a realistic and shared understanding of the patient’s needs, goals and values(C), then the team are empowered to make informed decisions, (M), which means timely palliative care provision is more likely (O) and unnecessary or avoidable resource use is minimised (O). |
| Integrating palliative care in oncology could help to improve prognostic understanding and awareness of patient preferences [28], empowering patients and providers with the knowledge and opportunity to reflect on potential consequences of decisions [77]. More frequent discussions with patients and interdisciplinary collaboration appears to reduce the intensity of care towards the end of life [62, 109], avoiding non-beneficial tests and treatment that are consequential to patient and system [48, 60, 77].  Integration that facilitates goals-of-care or serious illness conversations to occur earlier and more frequently increases the opportunities for person-centred care, which likely leads to improved symptom management and appropriateness of treatment regimens [48, 110]. This means that healthcare resources can be used more congruently with patient goals, needs and preferences [58, 64]. | |
| Illustrative quotes | |
| Abunasser, 2023 [62] | *“Good communications between the treating oncologists and the patient and family members to outline the expectations and treatment goals, can obviously minimize aggressive interventions including the administration of chemotherapy.”* |
| Wright, 2023 [8] | *“Engagement with patients… meant that nurses were aware of the threshold beyond which previously therapeutic actions could become harmful. They were vigilant in ensuring that this threshold not be crossed recklessly. To this end, nurses took seriously their role in communicating to physicians how patients tolerated and responded to interventions.”* |
| Raunkiaer, 2023 [55] | *“The stakes have to define the moment to withdraw treatment, both for the sake of saving costs and to provide patients with better quality of life and dignified death. This indicates a need for greater awareness from oncologists in the planning of and initiating conversations with families.”* |
| Zemplenyi, 2021 [111] | *“Patients managed by the PCCS team are less likely to die at the hospital… this is even more the case if patients are involved in palliative care earlier… Early palliative care… can add value to health care by reducing unnecessary costs associated with hospital stays and useless examinations and treatments.”* |
| Traeger, 2020 [90] | *“Patients and families disclosed struggles with current regimens, alongside feelings about delaying, stopping, or continuing cancer treatment. Clinicians drew on their knowledge of the patient’s experiences both within and outside of cancer care to facilitate recommendations—even when decisions remained fraught.”* |
| Emiloju, 2019 [26] | *“A discussion of the patient’s GOC provides the basis for alignment with the patient’s goals, while reducing the likelihood of recurrent hospitalizations if these are incongruent with the patient’s wishes.”* |
| Abbreviations | C: Context  GOC: Goals of care GPs: General practitioners  M: Mechanism  O: Outcome  PCCS: Palliative care consult service  QoL: Quality of life |

**References**

1. Kitta, A., et al., The silent transition from curative to palliative treatment: a qualitative study about cancer patients' perceptions of end-of-life discussions with oncologists. Support Care Cancer, 2021. 29(5): p. 2405-2413.

2. Weinstein, E., et al., Quality and cost outcomes of an integrated supportive care program. Support Care Cancer, 2022. 30(1): p. 535-542.

3. Tate, A., Death and the treatment imperative: Decision-making in late-stage cancer. Soc Sci Med, 2022. 306: p. 115129.

4. Gonzalez, R., et al., Impact of early vs late palliative care referrals on healthcare utilization in patients with pancreatic cancer. J Cancer Res Clin Oncol, 2023. 149(16): p. 14997-15002.

5. Kremenova, Z., et al., Does a Hospital Palliative Care Team Have the Potential to Reduce the Cost of a Terminal Hospitalization? A Retrospective Case-Control Study in a Czech Tertiary University Hospital. J Palliat Med, 2022. 25(7): p. 1088-1094.

6. Mashiro, E., H. Arao, M. Aoki, and Y. Matsumoto, What are the barriers to medical collaboration in community-based integrated care supporting cancer patients? A qualitative analysis of healthcare and long-term care providers' perceptions. Jpn J Clin Oncol, 2023. 53(12): p. 1162-1169.

7. Hahne, J., et al., Chinese physicians' perceptions of palliative care integration for advanced cancer patients: a qualitative analysis at a tertiary hospital in Changsha, China. BMC Med Ethics, 2022. 23(1): p. 17.

8. Wright, D.K., et al., "We're Cancer Care Nurses": Perceptions About Providing Palliative Care in a Community Hospital. J Hosp Palliat Nurs, 2023. 25(2): p. 82-89.

9. Schifferdecker, K.E., et al., Structure and integration of specialty palliative care in three NCI-designated cancer centers: a mixed methods case study. BMC Palliat Care, 2023. 22(1): p. 59.

10. Krause, R., L. Gwyther, and J. Olivier, Evaluating a vertical nurse-led service in the integration of palliative care in a tertiary academic hospital. Palliat Care Soc Pract, 2024. 18: p. 26323524231224806.

11. Lundeby, T., et al., Challenges and Learning Needs for Providers of Advanced Cancer Care: Focus Group Interviews with Physicians and Nurses. Palliat Med Rep, 2020. 1(1): p. 208-215.

12. van Gurp, J., et al., Integrating Palliative Care by Virtue of Diplomacy; A Cross-sectional Group Interview Study of the Roles and Attitudes of Palliative Care Professionals to Further Integrate Palliative Care in Europe. Int J Health Policy Manag, 2022. 11(6): p. 786-794.

13. Krause, R., L. Gwyther, and J. Olivier, The influence of context on the implementation of integrated palliative care in an academic teaching hospital in South Africa. Palliat Care Soc Pract, 2024. 18: p. 26323524231219510.

14. Okyere, J. and K. Kissah-Korsah, Barriers to the integration of palliative care in Ghana: evidence from a tertiary health facility. Palliat Care Soc Pract, 2023. 17: p. 26323524231179980.

15. Stewart, E., et al., Cancer centre supportive oncology service: health economic evaluation. BMJ Support Palliat Care, 2022.

16. Bakken, J., et al., Organizational structures influencing timely recognition and acknowledgment of end-of-life in hospitals - A qualitative study of nurses' and doctors' experiences. Eur J Oncol Nurs, 2023. 67: p. 102420.

17. Evans, J.M., et al., Integrating early palliative care into routine practice for patients with cancer: A mixed methods evaluation of the INTEGRATE Project. Psychooncology, 2019. 28(6): p. 1261-1268.

18. Sadang, K.G., et al., Clinician Perceptions of Barriers and Facilitators for Delivering Early Integrated Palliative Care via Telehealth. Cancers (Basel), 2023. 15(22).

19. Seow, H., et al., Effect of Early Palliative Care on End-of-Life Health Care Costs: A Population-Based, Propensity Score-Matched Cohort Study. JCO Oncol Pract, 2022. 18(1): p. e183-e192.

20. Garcia, R., et al., The Team-Based Serious Illness Care Program, A Qualitative Evaluation of Implementation and Teaming. J Pain Symptom Manage, 2023. 65(6): p. 521-531.

21. Sullivan, D.R., et al., Relationships among clinicians are crucial to successful palliative care integration: a qualitative study in lung cancer. Future Oncol, 2023. 19(3): p. 245-257.

22. Ferrell, B.R., R. Virani, E. Han, and P. Mazanec, Integration of Palliative Care in the Role of the Oncology Advanced Practice Nurse. J Adv Pract Oncol, 2021. 12(2): p. 165-172.

23. Lundeby, T., et al., A complex communication skills training program for physicians providing advanced cancer care - content development and barriers and solutions for implementation. J Commun Healthc, 2023. 16(1): p. 46-57.

24. Bennardi, M., et al., A qualitative analysis of educational, professional and socio-cultural issues affecting interprofessional collaboration in oncology palliative care. Patient Educ Couns, 2022. 105(9): p. 2976-2983.

25. Ullgren, H., L. Sharp, P. Fransson, and K. Bergkvist, Exploring Health Care Professionals' Perceptions Regarding Shared Clinical Decision-Making in Both Acute and Palliative Cancer Care. Int J Environ Res Public Health, 2022. 19(23).

26. Emiloju, O.E., D.A.M. Djibo, and J.G. Ford, Association Between the Timing of Goals-of-Care Discussion and Hospitalization Outcomes in Patients With Metastatic Cancer. Am J Hosp Palliat Care, 2020. 37(6): p. 433-438.

27. Hoek, D., et al., Role of GPs in shared decision making with patients about palliative cancer treatment: a qualitative study in the Netherlands. Br J Gen Pract, 2022. 72(717): p. e276-e284.

28. Sommer, J., C. Chung, D.M. Haller, and S. Pautex, Shifting palliative care paradigm in primary care from better death to better end-of-life: a Swiss pilot study. BMC Health Serv Res, 2021. 21(1): p. 629.

29. Toguri, J.T., L. Grant-Nunn, and R. Urquhart, Views of advanced cancer patients, families, and oncologists on initiating and engaging in advance care planning: a qualitative study. BMC Palliat Care, 2020. 19(1): p. 150.

30. Gott, M., et al., 'That's part of everybody's job': the perspectives of health care staff in England and New Zealand on the meaning and remit of palliative care. Palliat Med, 2012. 26(3): p. 232-41.

31. Johansen, M.L. and B. Ervik, Talking together in rural palliative care: a qualitative study of interprofessional collaboration in Norway. BMC Health Serv Res, 2022. 22(1): p. 314.

32. Bennardi, M., et al., A qualitative exploration of interactional and organizational determinants of collaboration in cancer palliative care settings: Family members', health care professionals' and key informants' perspectives. PLoS One, 2021. 16(10): p. e0256965.

33. Beiranvand, S., et al., Hospice care delivery system requirements. Int J Palliat Nurs, 2022. 28(12): p. 562-574.

34. Mayland, C.R., et al., A Qualitative Study Exploring Patient, Family Carer and Healthcare Professionals' Direct Experiences and Barriers to Providing and Integrating Palliative Care for Advanced Head and Neck Cancer. J Palliat Care, 2021. 36(2): p. 121-129.

35. Wind, J., et al., Who should provide care for patients receiving palliative chemotherapy? A qualitative study among Dutch general practitioners and oncologists. Scand J Prim Health Care, 2018. 36(4): p. 437-445.

36. Liu, Y., et al., Application of interdisciplinary collaborative hospice care for terminal geriatric cancer patients: a prospective randomized controlled study. Support Care Cancer, 2022. 30(4): p. 3553-3561.

37. Gardiner, C., M. Harrison, S. Hargreaves, and B. Taylor, Clinical nurse specialist role in providing generalist and specialist palliative care: A qualitative study of mesothelioma clinical nurse specialists. J Adv Nurs, 2022. 78(9): p. 2973-2982.

38. Collins, A., S.A. McLachlan, and J. Philip, Initial perceptions of palliative care: An exploratory qualitative study of patients with advanced cancer and their family caregivers. Palliat Med, 2017. 31(9): p. 825-832.

39. Desai, A.V., et al., Palliative Medicine in Myelodysplastic Syndromes: Patients and Caregivers - A Qualitative Study. BMJ Support Palliat Care, 2021.

40. Alizadeh, Z., et al., Challenges of Integrated Home-Based Palliative Care Services for Cancer Patients during the COVID-19 Pandemic: A Qualitative Content Analysis. Home Health Care Management & Practice, 2022. 35(3): p. 180-189.

41. Hayden, L. and S. Dunne, "Dying With Dignity": A Qualitative Study With Caregivers on the Care of Individuals With Terminal Cancer. Omega (Westport), 2022. 84(4): p. 1122-1145.

42. Zimmermann, C., et al., Perceptions of palliative care among patients with advanced cancer and their caregivers. CMAJ, 2016. 188(10): p. E217-E227.

43. Cerni, J., J. Rhee, and H. Hosseinzadeh, Challenges and strategies to improve the provision of end-of-life cancer care in rural and regional communities: Perspectives from Australian rural health professionals. Aust J Rural Health, 2023.

44. Perry, L.M., et al., Increasing Readiness for Early Integrated Palliative Oncology Care: Development and Initial Evaluation of the EMPOWER 2 Intervention. J Pain Symptom Manage, 2021. 62(5): p. 987-996.

45. Mollica, M.A., et al., Perspectives on Palliative Care in Cancer Clinical Trials: Diverse Meanings from Multidisciplinary Cancer Care Providers. J Palliat Med, 2018. 21(5): p. 616-621.

46. Rao, S.R., N. Salins, C.R. Goh, and S. Bhatnagar, "Building palliative care capacity in cancer treatment centres: a participatory action research". BMC Palliat Care, 2022. 21(1): p. 101.

47. Satija, A., et al., Quality Improvement in Itself Changes Your Thinking: Lessons From Disseminating Quality Improvement Methods Through a Multisite International Collaborative Palliative Care Project in India. JCO Glob Oncol, 2022. 8: p. e2200147.

48. Adelson, K., et al., Standardized Criteria for Palliative Care Consultation on a Solid Tumor Oncology Service Reduces Downstream Health Care Use. J Oncol Pract, 2017. 13(5): p. e431-e440.

49. Tartaglione, E.V., E.K. Vig, and L.F. Reinke, Bridging the Cultural Divide Between Oncology and Palliative Care Subspecialties: Clinicians' Perceptions on Team Integration. Am J Hosp Palliat Care, 2018. 35(7): p. 978-984.

50. Brenne, A.T., et al., Implementing a Standardized Care Pathway Integrating Oncology, Palliative Care and Community Care in a Rural Region of Mid-Norway. Oncol Ther, 2021. 9(2): p. 671-693.

51. Le, B.H., et al., Acceptability of early integration of palliative care in patients with incurable lung cancer. J Palliat Med, 2014. 17(5): p. 553-8.

52. Raunkiaer, M., M.S. Buch, C. Holm-Petersen, and H. Timm, Professionals' experiences with palliative care and collaboration in relation to a randomised clinical trial: a qualitative interview study. Scand J Caring Sci, 2020. 34(2): p. 305-313.

53. Economos, G., et al., Palliative care from the perspective of cancer physicians: a qualitative semistructured interviews study. BMJ Support Palliat Care, 2023. 13(1): p. 95-101.

54. McPherson, S., et al., Haematological nurses' experiences about palliative care trajectories of patients with life-threatening haematological malignancies: A qualitative study. Nurs Open, 2023. 10(5): p. 3094-3103.

55. Raunkiaer, M., et al., When and how to stop palliative antineoplastic treatment and to organise palliative care for patients with incurable cancer. Int J Palliat Nurs, 2023. 29(10): p. 499-506.

56. Zimmermann, C., et al., Symptom screening with Targeted Early Palliative care (STEP) versus usual care for patients with advanced cancer: a mixed methods study. Support Care Cancer, 2023. 31(7): p. 404.

57. Roen, I., et al., Spiritual quality of life in family carers of patients with advanced cancer-a cross-sectional study. Support Care Cancer, 2021. 29(9): p. 5329-5339.

58. Maessen, M., et al., An economic evaluation of an early palliative care intervention among patients with advanced cancer. Swiss Med Wkly, 2024. 154: p. 3591.

59. Collins, A., et al., The feasibility of triggers for the integration of Standardised, Early Palliative (STEP) Care in advanced cancer: A phase II trial. Front Oncol, 2022. 12: p. 991843.

60. Blackhall, L.J., et al., CARE Track for Advanced Cancer: Impact and Timing of an Outpatient Palliative Care Clinic. J Palliat Med, 2016. 19(1): p. 57-63.

61. Costantini, M., et al., Is early integration of palliative care feasible and acceptable for advanced respiratory and gastrointestinal cancer patients? A phase 2 mixed-methods study. Palliat Med, 2018. 32(1): p. 46-58.

62. Abunasser, M., et al., Aggressiveness of Cancer Care at End of Life in Patients with Metastatic Breast Cancer in Jordan. J Multidiscip Healthc, 2023. 16: p. 2873-2881.

63. Yang, G.M., S. Yoon, Y.Y. Tan, and K. Liaw, Experience and Views of Oncology and Palliative Care Professionals on a Corounding Model of Care for Inpatients With Advanced Cancer. Am J Hosp Palliat Care, 2018. 35(11): p. 1433-1438.

64. Thelen, M., S.G. Brearley, and C. Walshe, A grounded theory of interdependence between specialist and generalist palliative care teams across healthcare settings. Palliat Med, 2023. 37(10): p. 1474-1483.

65. Collins, A., et al., Communication about early palliative care: A qualitative study of oncology providers' perspectives of navigating the artful introduction to the palliative care team. Front Oncol, 2022. 12: p. 1003357.

66. Gross, J.P., et al., Radiation Oncologists' Role in End-of-Life Care: A Perspective From Medical Oncologists. Pract Radiat Oncol, 2019. 9(5): p. 362-370.

67. Montiel, C., et al., Barriers and facilitators of supportive care access and use among men with cancer: a qualitative study. J Cancer Surviv, 2023.

68. Dhollander, N., et al., Is early integration of palliative home care in oncology treatment feasible and acceptable for advanced cancer patients and their health care providers? A phase 2 mixed-methods study. BMC Palliat Care, 2020. 19(1): p. 174.

69. Howell, D.M., et al., Predictors of home care expenditures and death at home for cancer patients in an integrated comprehensive palliative home care pilot program. Healthcare policy = Politiques de sante, 2011. 6(3): p. e73-92.

70. Dhollander, N., et al., Phase 0-1 early palliative home care cancer treatment intervention study. BMJ Support Palliat Care, 2022. 12(e1): p. e103-e111.

71. Cushen-Brewster, N., et al., Evaluating a specialist palliative care service in a community setting. British Journal of Healthcare Management, 2023. 29(10): p. 1-13.

72. Hasegawa, T., et al., Integrating home palliative care in oncology: a qualitative study to identify barriers and facilitators. Support Care Cancer, 2022. 30(6): p. 5211-5219.

73. Schenker, Y., et al., A Pilot Trial of Early Specialty Palliative Care for Patients with Advanced Pancreatic Cancer: Challenges Encountered and Lessons Learned. J Palliat Med, 2018. 21(1): p. 28-36.

74. van Gurp, J., et al., Teleconsultation for integrated palliative care at home: A qualitative study. Palliat Med, 2016. 30(3): p. 257-69.

75. Ervik, B., T. Donnem, and M.L. Johansen, Dying at "home" - a qualitative study of end-of-life care in rural Northern Norway from the perspective of health care professionals. BMC Health Serv Res, 2023. 23(1): p. 1359.

76. Horlait, M., S. Van Belle, and M. Leys, The need for adequate communication training programs for palliative care in multidisciplinary teams in oncology settings. International Journal of Integrated Care, 2016. 16(6): p. 1-2.

77. Ellen, M.E., S. Perlman, and R. Shach, Too Much Cancer Care?: Nurses' Perspectives on the Unnecessary Use of Oncology Services. Cancer Nurs, 2021. 44(4): p. E236-E243.

78. van Dusseldorp, L., et al., What does the nurse practitioner mean to you? A patient-oriented qualitative study in oncological/palliative care. J Clin Nurs, 2019. 28(3-4): p. 589-602.

79. McCaughan, D., et al., Haematology nurses' perspectives of their patients' places of care and death: A UK qualitative interview study. Eur J Oncol Nurs, 2019. 39: p. 70-80.

80. Ribi, K., N. Kalbermatten, M. Eicher, and F. Strasser, Towards a novel approach guiding the decision-making process for anticancer treatment in patients with advanced cancer: framework for systemic anticancer treatment with palliative intent. ESMO Open, 2022. 7(3): p. 100496.

81. Johansen, M.L. and B. Ervik, Teamwork in primary palliative care: general practitioners' and specialised oncology nurses' complementary competencies. BMC Health Serv Res, 2018. 18(1): p. 159.

82. Lee, J.T., et al., Dying of mesothelioma: A qualitative exploration of caregiver experiences. Eur J Cancer Care (Engl), 2022. 31(5): p. e13627.

83. Lundereng, E.D., A. Dihle, and S.A. Steindal, Nurses' experiences and perspectives on collaborative discharge planning when patients receiving palliative care for cancer are discharged home from hospitals. J Clin Nurs, 2020. 29(17-18): p. 3382-3391.

84. Frissen, A.R., et al., Experiences of healthcare professionals with support for mesothelioma patients and their relatives: Identified gaps and improvements for care. Eur J Cancer Care (Engl), 2021. 30(6): p. e13509.

85. Spelten, E.R., et al., Making community palliative and end-of-life care sustainable; investigating the adaptability of rural Australian service provision. Health Soc Care Community, 2021. 29(6): p. 1998-2007.

86. Greer, J.A., et al., Cost Analysis of a Randomized Trial of Early Palliative Care in Patients with Metastatic Nonsmall-Cell Lung Cancer. J Palliat Med, 2016. 19(8): p. 842-8.

87. Mensah, A.B.B., et al., Expectations and barriers to the utilization of specialist palliative care services among persons living with cancer in Ghana: an exploratory qualitative study. Palliat Care Soc Pract, 2023. 17: p. 26323524231193042.

88. Roberson, M.L., et al., Re-imagining metastatic breast cancer care delivery: a patient-partnered qualitative study. Support Care Cancer, 2023. 31(12): p. 735.

89. Walshe, C., et al., 'Thank goodness you're here'. Exploring the impact on patients, family carers and staff of enhanced 7-day specialist palliative care services: A mixed methods study. Palliat Med, 2023. 37(10): p. 1484-1497.

90. Traeger, L., et al., Nature of Discussions about Systemic Therapy Discontinuation or Hospice among Patients, Families, and Palliative Care Clinicians during Care for Incurable Cancer: A Qualitative Study. Journal of Palliative Medicine, 2020. 23(4): p. 542-547.

91. Prod'homme, C., et al., Barriers to end-of-life discussions among hematologists: A qualitative study. Palliat Med, 2018. 32(5): p. 1021-1029.

92. Olafsdottir, K.L., et al., Integrating nurse-facilitated advance care planning for patients newly diagnosed with advanced lung cancer. Int J Palliat Nurs, 2018. 24(4): p. 170-177.

93. Collins, A., et al., The challenges and suffering of caring for people with primary malignant glioma: qualitative perspectives on improving current supportive and palliative care practices. BMJ Support Palliat Care, 2013. 4(1): p. 68-76.

94. Capodanno, I., et al., Caregivers of Patients with Hematological Malignancies within Home Care: A Phenomenological Study. Int J Environ Res Public Health, 2020. 17(11).

95. Henson, L.A., et al., 'I'll be in a safe place': a qualitative study of the decisions taken by people with advanced cancer to seek emergency department care. BMJ Open, 2016. 6(11): p. e012134.

96. Walton, L.M., J. Reeve, P.M. Brown, and C.M. Farquhar, Gynaecologic cancer patients' needs and experiences of supportive health services in New Zealand. Psychooncology, 2010. 19(2): p. 201-8.

97. Monnery, D., et al., Delivery Models and Health Economics of Supportive Care Services in England: A Multicentre Analysis. Clin Oncol (R Coll Radiol), 2023. 35(6): p. e395-e403.

98. Bakitas, M., K.D. Lyons, M.T. Hegel, and T. Ahles, Oncologists' perspectives on concurrent palliative care in a National Cancer Institute-designated comprehensive cancer center. Palliat Support Care, 2013. 11(5): p. 415-23.

99. Bull, J.H., et al., Demonstration of a sustainable community-based model of care across the palliative care continuum. J Pain Symptom Manage, 2012. 44(6): p. 797-809.

100. Hoverman, J.R., et al., Hospice or Hospital: The Costs of Dying of Cancer in the Oncology Care Model. Palliat Med Rep, 2020. 1(1): p. 92-96.

101. Bischoff, K.E., et al., Embedded palliative care for patients with metastatic colorectal cancer: a mixed-methods pilot study. Support Care Cancer, 2020. 28(12): p. 5995-6010.

102. Ervik, B., B. Brondbo, and M.L. Johansen, Adapting and Going the Extra Mile: A Qualitative Study of Palliative Care in Rural Northern Norway From the Perspective of Healthcare Providers. Cancer Nurs, 2021. 44(4): p. E229-E235.

103. Senior, L. and G. Hubbard, Integrated malignant and non-malignant palliative care in Scotland. Br J Community Nurs, 2010. 15(6): p. 284-91.

104. Hojjat-Assari, S., M. Rassouli, V. Kaveh, and H. Heydari, Explaining health care providers' perceptions about the integration of palliative care with primary health care; a qualitative study. BMC Prim Care, 2022. 23(1): p. 226.

105. Yang, G.M., et al., Comparing the effect of a consult model versus an integrated palliative care and medical oncology co-rounding model on health care utilization in an acute hospital - an open-label stepped-wedge cluster-randomized trial. Palliat Med, 2021. 35(8): p. 1578-1589.

106. Siegle, A., et al., Communication with patients with limited prognosis-an integrative mixed-methods evaluation study. Support Care Cancer, 2022. 31(1): p. 77.

107. Hannon, B., et al., Early Palliative Care and Its Role in Oncology: A Qualitative Study. Oncologist, 2016. 21(11): p. 1387-1395.

108. Dhollander, N., et al., Differences between early and late involvement of palliative home care in oncology care: A focus group study with palliative home care teams. Palliat Med, 2018. 32(7): p. 1275-1282.

109. Fox, J.A., J. Rosenberg, S. Ekberg, and D. Langbecker, Palliative care in the context of immune and targeted therapies: A qualitative study of bereaved carers' experiences in metastatic melanoma. Palliat Med, 2020. 34(10): p. 1351-1360.

110. Chang, S., et al., A Palliative Radiation Oncology Consult Service Reduces Total Costs During Hospitalization. J Pain Symptom Manage, 2018. 55(6): p. 1452-1458.

111. Zemplenyi, A.T., et al., Early palliative care associated with lower costs for adults with advanced cancer: evidence from Hungary. Eur J Cancer Care (Engl), 2021. 30(6): p. e13473.
